# Prediction of hemoglobin levels in individual hemodialysis patients by means of a mathematical model of erythropoiesis

**DOI:** 10.1371/journal.pone.0195918

**Published:** 2018-04-18

**Authors:** Doris H. Fuertinger, Franz Kappel, Hanjie Zhang, Stephan Thijssen, Peter Kotanko

**Affiliations:** 1 Renal Research Institute, New York, New York, United States of America; 2 Institute for Mathematics and Scientific Computing, Karl-Franzens University, Graz, Austria; 3 Icahn School of Medicine at Mount Sinai, New York, New York, United States of America; Lady Davis Institute for Medical Research, CANADA

## Abstract

Anemia commonly occurs in people with chronic kidney disease (CKD) and is associated with poor clinical outcomes. The management of patients with anemia in CKD is challenging, due to its severity, frequent hypo-responsiveness to treatment with erythropoiesis stimulating agents (ESA) and common hemoglobin cycling. Nonlinear dose-response curves and long delays in the effect of treatment on red blood cell population size complicate predictions of hemoglobin (Hgb) levels in individual patients. A comprehensive physiology based mathematical model for erythropoiesis was adapted individually to 60 hemodialysis patients treated with ESAs by identifying physiologically meaningful key model parameters from temporal Hgb data. Crit-Line^®^ III monitors provided non-invasive Hgb measurements for every hemodialysis treatment. We used Hgb data during a 150-day baseline period together to estimate a patient’s individual red blood cell lifespan, effects of the ESA on proliferation of red cell progenitor cells, endogenous erythropoietin production and ESA half-life. Estimated patient specific parameters showed excellent alignment with previously conducted clinical studies in hemodialysis patients. Further, the model qualitatively and quantitatively reflected empirical hemoglobin dynamics in demographically, anthropometrically and clinically diverse patients and accurately predicted the Hgb response to ESA therapy in individual patients for up to 21 weeks. The findings suggest that estimated model parameters can be used as a proxy for parameters that are clinically very difficult to quantify. The presented method has the potential to provide new insights into the individual pathophysiology of renal anemia and its association with clinical outcomes and can potentially be used to guide personalized anemia treatment.

## Introduction

A decrease in the total amount of red blood cells (RBC) and hemoglobin (Hgb) levels impairing the blood’s ability to carry oxygen is referred to as anemia. Anemia is a common occurrence in patients suffering from chronic kidney disease (CKD). In 2014, 84% of 680,000 end-stage renal disease patients in the US were treated for anemia by administering erythropoiesis stimulating agents (ESA) [1]. The administration of ESAs exerts hematological effects similar to the endogenous hormone erythropoietin (EPO) which drives the production of new RBCs. Based on several randomized controlled trials the most current clinical practice guideline calls for a partial correction of anemia in CKD patients, urging inception of therapy at a Hgb of 9.0 to 10.0 g/dl, and treatment to levels in general not beyond 11.5 g/dl [2].

Renal anemia is often severe and difficult to treat not least due to a high inter-individual variability in response to ESA therapy. The reasons underlying anemia include insufficient RBC production, abnormally elevated RBC breakdown, and blood loss (e.g. gastro-intestinal bleeding episodes). Routine measurements of quantities, such as RBC lifespan, bone marrow response to ESA, EPO levels and individual half-life of the administered ESA compound, are either not practical or infeasible in a clinical environment. Patient-specific predictions of the response to ESA administration are extremely challenging mainly due to the long delay and non-linearity in reaction of the RBC population to ESA treatment. Hence, Hgb levels in patients frequently fail to achieve recommended Hgb targets and Hgb levels in hemodialysis (HD) patients tend to fluctuate widely and are prone to exhibit cyclic behavior [3, 4].

Erythropoiesis is a complex physiological process requiring the orchestrated activation of numerous regulatory systems on multiple spatial and temporal scales. Thus, a comprehensive mechanistic mathematical model based on physiological considerations is particularly well suited to examine the intricate dynamics of bone marrow activity in response to EPO in different individuals. Detailed computer simulations permit further insights into reasons for non-responsiveness of patients to ESA therapy and Hgb cycling. Further, a model that gives reliable estimates for physiological parameters may be used to assess correlative patterns between clinical and in-silico parameters.

The model presented in this study is not the first mathematical model to describe the dynamics of erythropoiesis. The earliest efforts to develop such a model date back to the 1990s with most approaches inspired by the work of Belair and colleagues [5]. Several authors modified, analyzed and adapted the Belair model to different experimental data. For example, an improved and extended version of this model was published by Mahaffy et al. [6] who performed model fitting based on experimental recordings of rabbits with induced auto-immune hemolytic anemia. Later on, the authors adapted the same model to phlebotomy data in humans [7]. A later study, which was also inspired by [5], focused on an application to periodic hematological diseases [8] and Crauste and colleagues [9–11] presented a different modification of the Belair model incorporating self-renewal of progenitor cells. A rigorous theoretical analysis of the Belair model and some of its modifications can be found, for example, in [12, 13] and [14]. Besides the seminal work of Belair, Loeffler and collaborators made substantial contributions to erythropoiesis modeling, see e.g. [15–17]. Later publications (see e.g. [18, 19]) focused primarily on modeling of hematopoietic stem cells.

The mathematical model used in this study is a comprehensive age-structured cell population model, which distinguishes granularly between different erythroid cell stages [20]. As distinct from previously developed models, it incorporates neocytolysis, a mechanism that has been confirmed in a variety of physiologic and pathophysiologic situations [21], including the anemia of renal failure [22], [23]. Erythropoietin suppression triggers neocytolysis, a selective eryptosis of neocytes, the youngest circulating red blood cells. The pathologic erythropoietin deficiency of renal disease precipitates neocytolysis, which contributes to dosing differences between different ESA treatment regimens [24].

Comprehensive physiology based models are commonly used to generate insights and test hypotheses about underlying mechanisms of diseases. Such models are rarely individualized using patient specific data as they are often thought to be too complex to be adaptable using clinically available data. We explored the feasibility of personalizing a comprehensive model for erythropoiesis using clinical data in a cohort of 60 anemic HD patients. Adaptation and prediction of individual Hgb levels showed excellent results. Moreover, individually estimated parameters that are of physiological key importance allow further insight into patient specific pathophysiology.

## Materials and methods

### Ethics statement

The study was approved by the Beth Israel Institutional Review Board (# 087–13) and the New England Institutional Review Board (# 15–291) and conducted in accordance with the Declaration of Helsinki. Informed consent was not obtained as this was determined not to be human subject research, and we were working with de-identified data.

### Study design

A mathematical model of erythropoiesis [20] was adapted to Hgb recordings from individual HD patients treated with epoetin alfa (EPOGEN^®^, Amgen, Thousand Oaks, CA). Epoietin alfa is a human erythropoietin produced in cell culture using recombinant DNA technology. It has a molecular weight of 18,396 dalton, whereas endogenous erythropoietin has a molecular weight of approximately 30,400 dalton; the difference in molecular weight is explained by different glycosylation patterns. Retrospective data from the Renal Research Institute (RRI) and dialysis facilities of Fresenius Medical Care across the United States between April 2012 and July 2014 were used.

In these clinics use of the CLM is part of standard care, albeit with some utilization variability. A baseline period of 5 months was defined on a patient level for parameter estimation purposes. In general, patients visit the HD center 3 times a week, i.e. about 65 treatments are expected to occur during baseline. Only patients with more than 42 eligible CLM measurements and at least 2 epoetin alfa administrations during the model adaptation period were considered. Hgb measurements supplemented with simulation results obtained from the mathematical model were used to estimate patient’s individual erythrocyte lifespan, bone marrow response to the ESA (slopes of the apoptosis and maturation velocity functions of erythroid precursor cells), endogenous EPO production, and EPOGEN^®^ half-life.

### Data acquisition & eligibility

The CLM provides quasi-continuous non-invasive measurements of hematocrit during hemodialysis. The method is based on an optical sensor technique. The sensor is attached to a blood chamber placed in the extracorporeal circuit. The measurements are based on both the absorption properties of the hemoglobin molecule and the scattering properties of red blood cells. In most patients CLM measurements are available for every dialysis treatment.

The first and last Hgb recordings of eligible treatments were used for this study, subsequently referred to as “pre-dialysis” and “post-dialysis” values. All data points with pre-dialysis Hgb readings less than 5 g/dl and larger than 20 g/dl were excluded. All 60 patients were treated with recombinant human erythropoietin also commonly referred to as epoetin alfa, which was the most commonly used drug in the US at the time of the study (96.5% of US HD patients received epoetin alfa according to USRDS report at the time of the study [1]).

### A physiologically based model for erythropoiesis

The mathematical model of erythropoiesis used in this study is based on structured population models and describes the production of red blood cells from stem cells in the bone marrow to mature erythrocytes circulating in the blood stream. Cell types are grouped into population classes according to their characteristic properties with respect to interaction with EPO. These properties include the proliferation rate, the rate of apoptosis and the maturation velocity of cells, which—depending on the cell type—may vary depending on EPO levels. Five different age-structured classes of cell populations are considered: BFU-E, CFU-E, erythroblasts, marrow reticulocytes and erythrocytes (including blood reticulocytes). Each population class can be described by the following partial differential equation

$$\begin{array}{l} (\frac{\partial }{\partial t}u\left(t,\mu \right)+\nu \left(E\left(t\right)\right)\frac{\partial }{\partial \mu }u\left(t,\mu \right)=(\beta \left(E\left(t\right)\right)-\alpha \left(E\left(t\right),\mu \right)u\left(t,\mu \right),\\ v\left(E\left(t\right)\right)u\left(t,0\right)=f\left(t\right),\\ u\left(0,x\right)=u_{0}\left(x\right). \end{array}$$

where *u*(*t*, ·) is the population density with respect to the cell maturity *μ* at time *t*. Further, the functions *β*(·) and *α*(·) describe the proliferation rate and the rate of apoptosis, respectively and the function *v*(·) denotes the maturation velocity of cells in this population class. The boundary condition is given by the function *f*(·), which describes the influx of cells with maximal maturity from the previous population class and *u*_0_(·) is the initial density of the population. The model presented in [20] further consists of two ordinary differential equations describing the dynamics of exogenous and endogenous EPO over time. The release of endogenous EPO in healthy adults involves a feedback loop that is regulated by the number of red blood cells circulating in the blood. This feedback mechanism is heavily impaired in dialysis patients and we assume a constant release of endogenous EPO, independent of the size of the red blood cell population, i.e. EPO serum levels are assumed to be constant for individual patients. Further, in case of blood loss or blood transfusions the last population class (erythrocytes) can be decreased or increased accordingly to match the data. For instance, in the US about a pint of blood (473 ml) is donated with an average cell count of 5 * 10^9^ cells per ml [25, 26]. Thus, one blood transfusion adds approximately 23.65 * 10^11^ cells to the erythrocyte population class.

### Parameter estimation

Individual Hgb and ESA administration data over a 150-day period were used to adjust the model and identify key biological characteristics of the specific patient. Gender, height and (average) post-dialytic weight of the patient was used to estimate post-dialytic blood volume using the Nadler formula [27]. The number of stem cells committing to the erythroid lineage was adjusted based on the patient’s blood volume and a steady state assumption for the model. The following model parameters were estimated for each individual: RBC Lifespan, endogenous erythropoietin levels, half-life of the administered ESA, the slope of the apoptosis of hematopoietic colony forming units and the slope of the maturation velocity of bone marrow reticulocytes.

The employed parameter identification scheme has been thoroughly presented in [28]. Briefly, a least-squares cost-functional was minimized in order to determine the parameter estimates. A fast and robust numerical app*roximation scheme based on semi*group theory was constructed and implemented in Python to solve the system of partial differential equations describing the cell populations of the erythroid lineage. Multiple parameter identification runs are initiated with different initial conditions. Initial guesses for the parameters were chosen within physiological reasonable ranges. A direct search method was used to find minima of the cost-functional and we did not restrict the parameter search to a predefined area, i.e. we used an unconstrained optimization technique. The Nelder-Mead simplex method implemented in the SciPy package for Python was used for this purpose [29]. The parameter identification was restarted around local minima with low residual values of the cost functional, by slightly perturbing the optimal parameter values (around ± 10%) to avoid being a stuck in a non-optimal point. *Further*, for some patients we iterated over the parameters separately to check for any further improvements around the local minima.

The post-dialytic CLM measurements of hemoglobin levels were chosen for parameter identification purposes. Based on previous research the estimation of the post-dialytic blood volume using the empirical Nadler formula [27] is slightly more accurate than for the pre-dialysis blood volume. However, in the medical community the pre-dialysis Hgb values are more commonly accepted. Thus, we calculate the simulated pre-dialysis Hgb and present these results in the paper. We use the following formula to determine the simulated pre-dialysis Hgb:

$$preTBV=\frac{postHgb_{CLM}*postTBV}{preHgb_{CLM}},\;preHgb_{sim}=\frac{postHgb_{sim}*postTBV}{preTBV},$$

where *preTBV* describes the calculated pre-dialysis blood volume and *postTBV* refers to the post-dialytic blood volume which is estimated using the Nadler formula. Further, *postHgb*_*CLM*_ and *preHgb*_*CLM*_ are the post-dialysis and pre-dialysis Hgb measurements and *postHgb*_*sim*_ and *preHgb*_*sim*_ denote the simulated post-dialysis and pre-dialysis Hgb levels, respectively.

The error between the model and data is reported as the mean absolute percentage error, which is a standard measure of prediction accuracy of forecasting methods and is defined as

$$MAPE=\frac{100}{N}\sum_{i=1}^{N}|\frac{y(t_{i})-g(t_{i})}{y(t_{i})}|,$$

where *N* is the number of observations, *y*(*t*_*i*_) denotes the measurements and *g*(*t*_*i*_) describes the model output at the time points *t*_*i*_, *i* = 1, …, *N*, respectively.

Comparison between the one dimensional distribution of the estimated model parameter *p*_*est*_ and literature data on the distribution of the physiological parameter *p*_*data*_ is done using the Kantorovich distance (also known as Wasserstein or earth mover’s distance). The metric quantifies the minimum amount of “work” required to transform *p*_*est*_ into *p*_*data*_, where “work” means the amount of distribution weight that must be moved times the distance it needs to be moved. The Kantorovich distance is defined as

$$l(p_{est},p_{data})=\underset{\;\pi \in \Gamma (p_{est},p_{data})}{\text{inf}}\int_{R\times R}^{}|x-y|d\pi (x,y),$$

where Γ(*p*_*est*_, *p*_*data*_) is the set of distributions on R×R whose marginals are *p*_*est*_ and *p*_*data*_. A Scipy implementation was used to calculate the first Wasserstein distance [29].

## Results

A comprehensive physiologically informed mathematical model of erythropoiesis was adapted to 60 chronic HD patients using parameter estimation techniques. Crit-Line^®^ III Monitors (CLM; Fresenius Medical Care, Concord, CA) were used to measure hemoglobin (Hgb) on a per-treatment level. The mean age of the HD patients was 59.4 ± 14.7 years, dialysis vintage was 3.63 ± 3.3 years, 48.3% were female, 51.7% were black, and 58.3% were diabetic. The body mass index in this cohort was 27.9 ± 6.7 kg/m^2^. All patients received intravenous iron (16.9 ± 6.8 mg) and their transferrin saturation and ferritin levels indicate sufficient iron availability on a population level. For more details on the patient characteristics see Table 1. Two patients experienced bleeding episodes, which we accounted for in the model simulations.

**Table 1:** Population demographics.

Parameter	Mean ± SD	Parameter	Mean ± SD
Age [years]	59.4 ± 14.7	Neutrophil-to-lymphocyte ratio	3.59 ± 2.01
Interdialytic weight gain [kg]	2.56 ± 0.88	Height [cm]	166.5 ± 9.7
Pre-dialysis weight [kg]	78.6 ± 19.7	Vintage [years]	3.63 ± 3.30
Albumin [g/dl]	4.01 ± 0.28		Percentage [%]
Epoetin alfa dose [U/kg/treatment]	17.94 ± 17.66	White [%]	48.33
Ferritin [ng/ml]	1005 ± 544	Male [%]	51.67
Transferrin saturation [%]	36.41 ± 6.94	Diabetic [%]	58.33
Intravenous iron dose [mg per dialysis session]	16.89 ± 6.79	Central venous catheter as vascular access [%]	8.33

### Model adaptation

**Fig 1:**
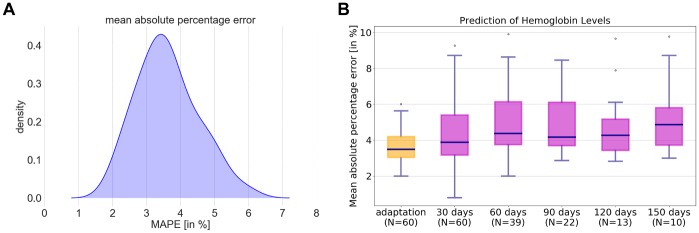
Mean absolute percentage errors (MAPE) between model simulation and individual patient data. Density of MAPE of hemoglobin levels between empirical patient data and simulated patient data during the model adaption period (Panel A). Panel B depicts Box-and-Whisker plots of the MAPE for the adaption period (yellow) and for every 30 days during the prediction period (magenta). *N* denotes the number of patients that have been followed-up.

On average, Hgb levels of patients were recorded in 53 ± 7 treatments during a 150 day period. This data was used to estimate model parameters on a per patient level. The model closely resembled the Hgb dynamics observed in the empirical recordings and showed a remarkable approximation quality of the data. The model was adjusted to 60 individuals with a consistently high quality, demonstrating the excellent model fidelity. The mean absolute percentage error (MAPE) for the model adaptation over the population cohort was 3.6 ± 0.9%. In Fig 1A the distribution of the MAPE for the 60 patients is plotted. The 75^th^ percentile of the error between measurements and model output is with 4.2% very low. Moreover, the maximum MAPE of 6% in the patient cohort underscores the excellent quality of the model adaptations.

**Fig 2:**
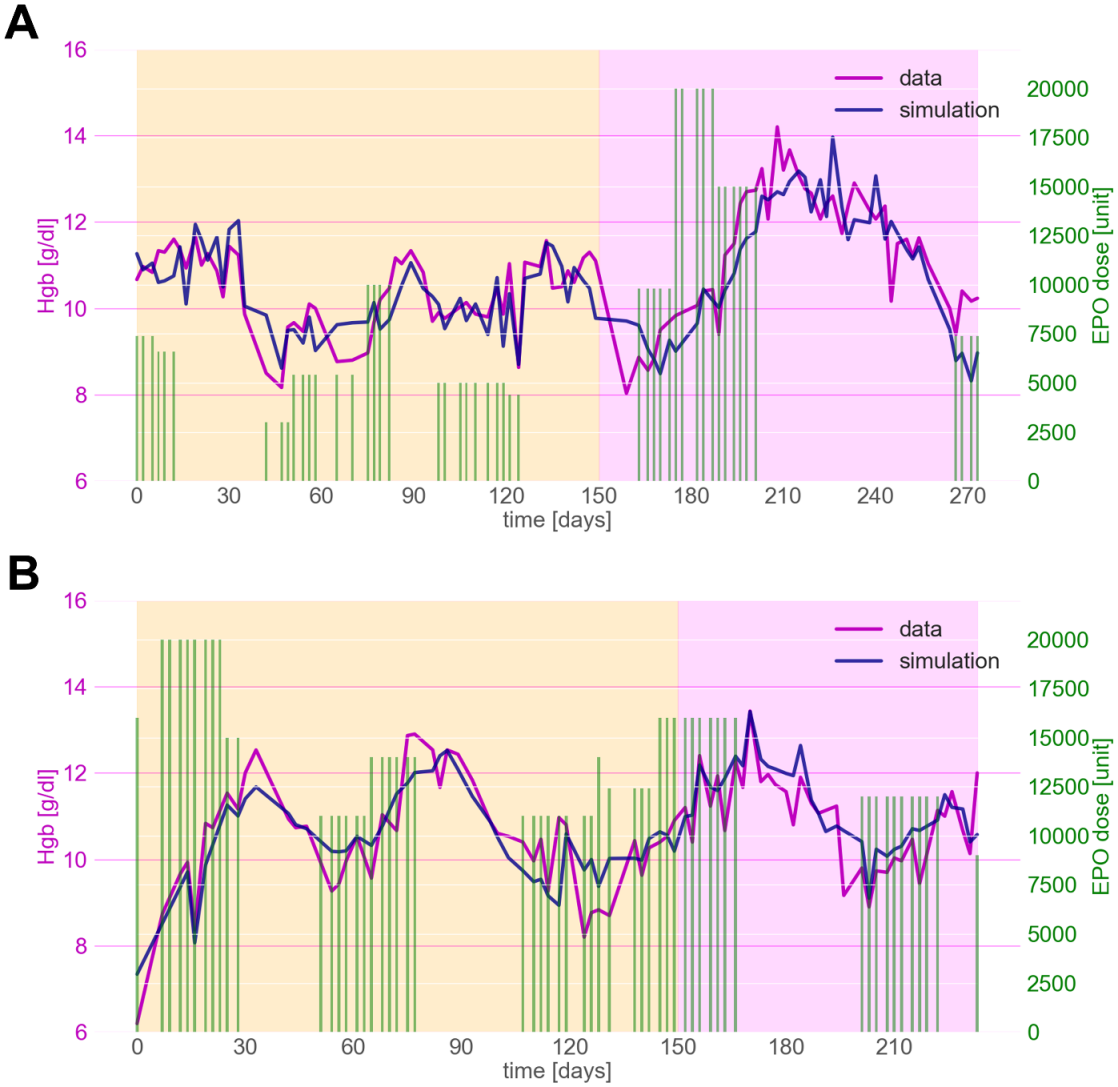
Comparison of model simulations and empirical data for two patients. Pre-dialysis Hgb measurements (magenta) and model output (blue) during the model adaptation period (yellow area) and prediction period (purple area). Green bars represent the administered ESA doses.

Fig 2 illustrates model adaptation and prediction in two exemplary dialysis patients of very different demographics, anthropometrics and comorbidities—all factors that potentially influence the severity of anemia and required ESA usage to correct the anemia. Fig 2A depicts Hgb dynamics of a young, class II obese, non-diabetic, black male, while the lower panel shows the temporal evolution of Hgb in an elderly, normal-weight, diabetic, white female. The model can be adapted to both individuals with a similar accuracy (MAPE: 5.16% and 4.9%, respectively.). Notably, the simulations resemble the observed Hgb dynamics exceptionally well under the respective ESA regimen. For further details on the patient characteristics and the individual determined model parameters see Table 2. Figures for all individual model adaptations and predictions can be found in the supplemental material S1 Figs.

**Table 2:** Characteristics of individual patients.

Patient characteristics	Patient 1	Patient 2	Patient 3
Race	Black	White	Black
Age [years]	36	84	66
Gender	Male	Female	Male
Height [cm]	187	155	170
Weight [kg]	136	62	54
Diabetes	No	Yes	No
RBC Lifespan [days]	74	92	81
Endogenous EPO level [U/l]	9.8	5.2	9.5
Epoetin alfa half-life [h]	9.6	4.8	4.4
Bone marrow reaction (apoptosis rate parameter, maturation velocity parameter)	Suppressed (0.0087, 0.0196)	Normal (0.016, 0.0488)	Slightly suppressed (0.0079,0.84)
Gastro-intestinal bleeding	No	No	Yes

Characteristics of patients presented in Figs 2A (Patient 1), 2B (Patient 2) and 3 (Patient 3).

**Fig 3:**
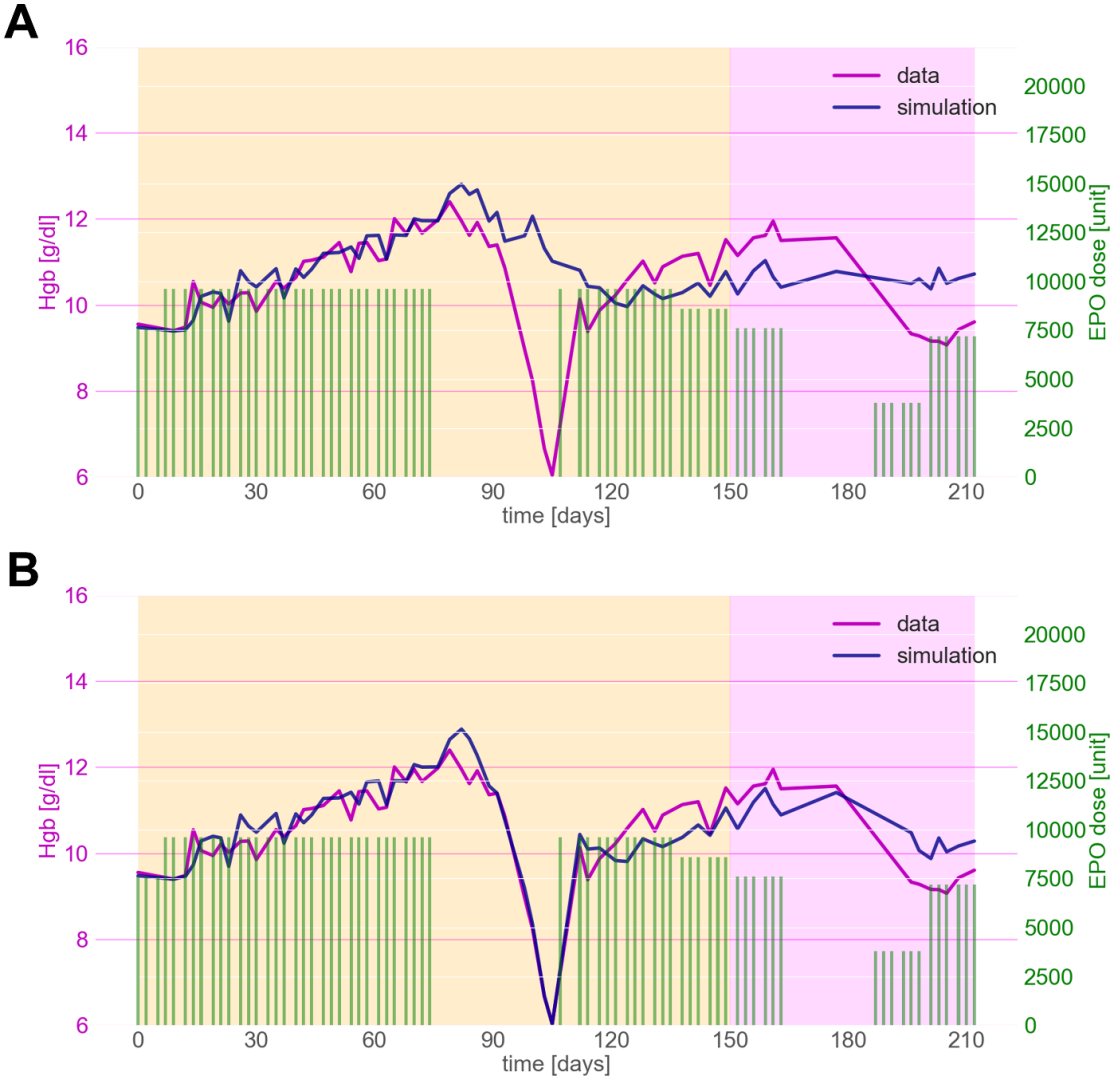
Model adaptation and prediction for a patient experiencing a severe bleeding episode. Pre-dialysis CLM Hgb levels (magenta) and model output (blue) during the model adaptation period (yellow area) and prediction period (purple area). Green bars represent the administered ESA doses. Model output without and with considering the bleeding and subsequent blood transfusion are presented in Panel A and B, respectively.

Fig 3 depicts Hgb levels of a senior, normal-weight, non-diabetic, black male. He suffered from a gastro-intestinal bleeding episode of several days starting around day 85 of the model adaptation period. Due to the severity of the resulting anemia the patient received a transfusion of packed red blood cells. Unsurprisingly, initial model adaptation attempts that did not consider this hemodynamic incident failed to account for the dynamics during and after the bleeding episode (see Fig 3A). Thus, we incorporated the patient’s gastro-intestinal bleeding episode to obtain a new simulation without changing the previously estimated set of model parameters for this individual. The resulting model output can be seen in Fig 3B. The model fully accounts for variations in Hgb dynamics during and after the bleeding episode without requiring a re-adjustment of patient-specific model parameters.

### Model prediction

Depending on the availability of follow-up data we predicted individual Hgb levels for up to 150 days. The average follow-up time was 84 ± 46 days (range: 25–172 days), with Hgb recordings available in 23 ± 15 of the treatments during this time. The adaptation period served to individualize the model. Patient specific model parameters estimated during the adaptation period were used to simulate Hgb levels in the prediction period. The individually determined model parameters were kept constant during the entire baseline and follow-up time. The model accurately predicts future hemoglobin levels and dynamics in individual patients for up to 150 days.

Fig 1B shows a Box-and-Whisker plot of the mean percentage errors for the adaptation period and for every 30 days of the prediction period. The MAPE for the adaptation period and the prediction period was calculated separately. Median, 25^th^ and 75^th^ percentiles as well as minimum and maximum relative errors remain stable over the prediction period. The available duration of per treatment Hgb recordings varies highly between individuals. We had access to eight months of CLM Hgb data in 22 patients, which was divided into 150 days of adaptation period and 90 days of prediction period. The median relative errors during the prediction period were 3.9% after 30 days (N = 60), 4.4% after 60 days (N = 39), and 4.2% after 90 days (N = 22). Further, the 90^th^ percentile for the model prediction error remained stable with 6.7%, 7.2%, and 7.2% after 30, 60, and 90 days respectively. An extended follow-up time of 120 days was available in 13 patients and 10 patients had a prediction period of 150 days. In the small cohort with prediction horizons longer than 90 days, median relative errors remained stable (4.3% after 120 days, 4.9% after 150 days) and the 90 percent of the patients had a relative mean error of less than 7.5% and 8.8% after 120 and 150 days, respectively.

The predictions of individual Hgb levels depicted in Figs 2 and 3 show the excellent capability of the model to reflect Hgb dynamics over an extended period of time. Notably, the ESA regimen of the patient depicted in Fig 2A changed considerably from the adaptation period to the prediction phase, with doses being administered that were twice as high as the highest dose during the adaptation period. Nonetheless, the model predictions are qualitatively and quantitatively excellent and show that the model can easily extrapolate beyond previously seen Hgb dynamics.

### Model parameters

**Fig 4:**
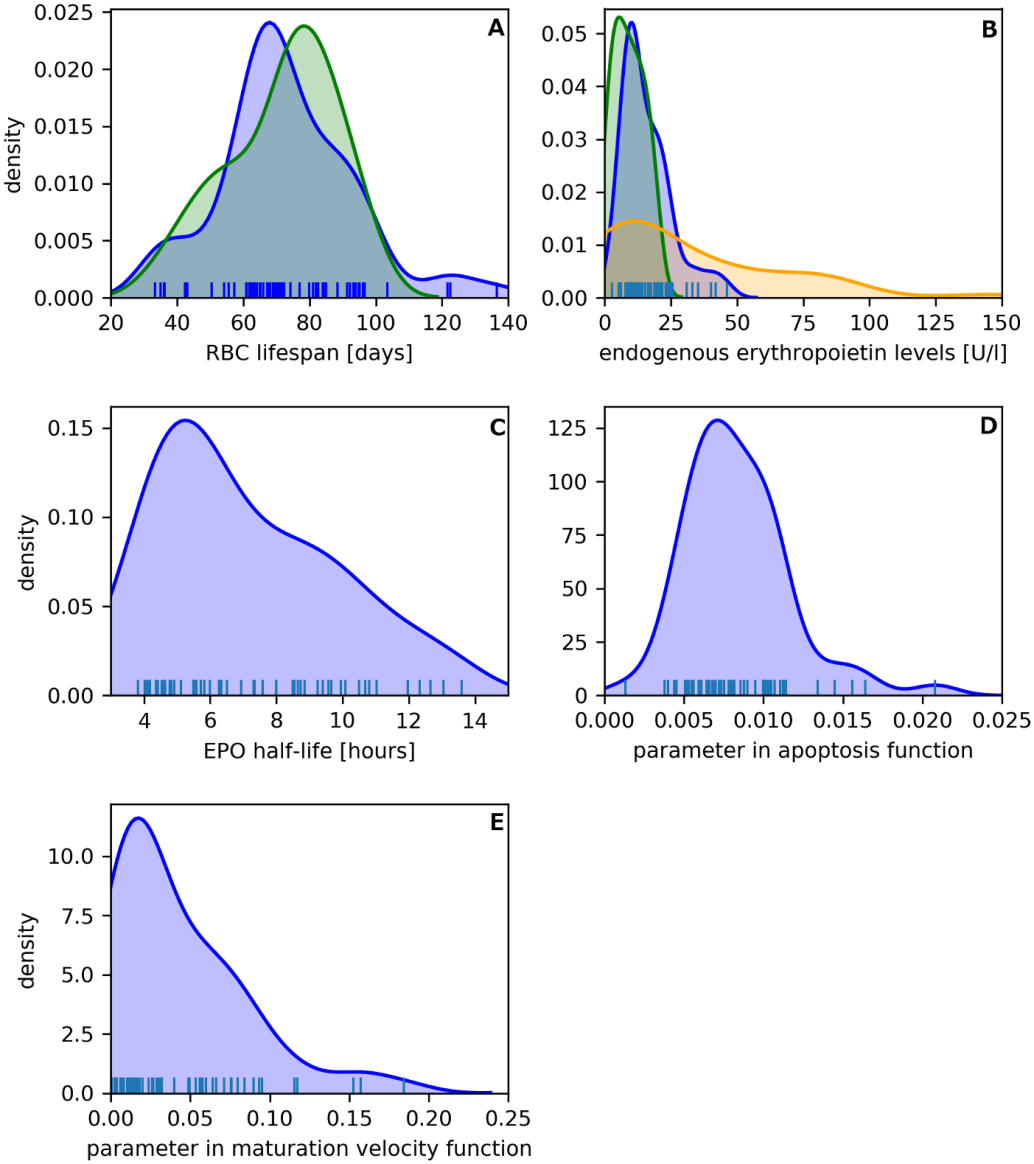
Comparison of estimated and clinically measured parameters. Panel A shows the density of estimated RBC lifespan in 60 HD patients (blue) compared to measurements in a clinical study conducted in 54 HD patients (green) (data adapted from [30]). Panel B compares estimated endogenous EPO levels in 60 HD patients (blue) and clinical study data in CKD 5 patients (N = 39, green) and CKD 1 or 2 patients (N = 45, orange) (data adapted from [31]). Panel C-E depict the densities of the estimated model parameters for EPO half-life, the slope parameter in the apoptosis function and the slope parameter in the maturation velocity function. Individual parameters are shown as sticks on the x-axis in all panels.

Model parameters were estimated on a per-subject basis during the adaptation period using each patient’s individual recordings. The adjusted model parameters, such as RBC lifespan and endogenous EPO production show a remarkable alignment with previously reported values in clinical studies in HD patients (Fig 4). Ma and colleagues [30] measured red blood cell lifespan in 54 HD patients. Their results and the estimated RBC lifespan of the 60 HD patients presented by us show very similar characteristics (see Fig 4A). The mean RBC lifespan in [30] was 73 ± 18 days (range: 38–116 days) compared to a mean estimated RBC lifespan in our study of 73 ± 20 days (range: 33–137 days). The Kantorovich (Wasserstein) distance between the estimated parameters and the measured literature values is 4.15. To provide the reader with a reference value we compared the literature data to a uniform distribution on the interval (33, 137), which resulted in a Kantorovich distance of 43.2.

Artunc and Risler measured endogenous EPO concentrations in 500 patients with varying degrees of anemia and chronic kidney disease (ranging from CKD 1 to CKD5, where CKD 5 includes end-stage renal disease) [31]. Results of the CKD 5 group (n = 39) and patients with CKD 1 or 2 (n = 45) together with the estimated model parameters of our cohort of 60 HD patients are presented in Fig 4B. The Kantorovich distance between the two distributions is 6.86. EPO levels of patients with less severe renal insufficiency were substantially higher than those with CKD 5 (Kantorovich distance: 22.73). This finding can be attributed to increasingly impaired EPO production in the kidneys with progressing CKD stages [32].

Further, half-life of Epoetin alfa is reported to be 4–12 hours in dialysis patients [33], which is very similar to the range of 3.8–13.6 hours (median: 6.3 hours) we estimated in our patient cohort (see Fig 4C). We are not aware of studies in end-stage renal disease patients that quantify changes in erythroid precursor cell apoptosis rate or maturation velocity in response to erythropoietin levels. Hemodialysis patients have, in general, an impaired bone marrow response compared to healthy subjects [34] and we observed a similar effect in the model’s parameters, when comparing bone marrow characteristics determined for healthy subjects to values estimated for individual HD patients. The model parameters for the slope of the apoptosis prevention function and the maturation velocity for a healthy population was 0.02 and 0.08, respectively, in [20]. The median parameter values for HD patients for the slope of the apoptosis prevention function and the slope of the maturation velocity function of precursor cells was 0.0079 (range: 0.0013–0.0208) and 0.029 (range: 0.0007–0.1864), respectively (see Fig 4D and 4E). Taken together, a lowered rate of apoptosis inhibition and lower maturation velocity in the model indirectly reflects a lowered efficiency of the ESA.

## Discussion

Our mathematical model of erythropoiesis demonstrated its ability to provide physiologically reasonable parameter estimates on a patient level. Further, the model accurately predicted future hemoglobin levels and patterns in individual patients for up to 21 weeks while being simultaneously capable of reflecting Hgb dynamics in anthropomorphically and clinically very different patient phenotypes. Moreover, the model can account for bleedings and blood transfusions, which frequently occur in dialysis patients, without requiring a re-adjustment of the model in its structure or patient-specific parameters.

The presented work provides means to obtain further insights into the underlying causes of renal anemia without the need of invasive measurements. To the extent that clinical data are available, the patient-specific estimated model parameters align well with previously reported values. Our findings clearly indicate that the model parameters have a physiologically meaningful interpretation, which suggests that the presented in-silico estimates are a proxy for parameters that are otherwise very difficult to quantify. The only information required to adjust the model and estimate individual patient-specific parameters is the patient’s gender, height, weight as well as ESA administration over the last five months and corresponding Hgb levels measured every hemodialysis treatment during this time period. Notably, we did not rely on Hgb levels measured in blood samples but used a non-invasive measurement method—CLM—that is standard of care in a substantial number of US dialysis facilities. The availability of many observations on each individual (53±7 hemoglobin measurements) allowed us to use continuous, deterministic optimization techniques to solve the inverse problem to adapt the individual model parameters. These techniques do not use the information known on other individuals. In situations where e.g. only bi-weekly or monthly lab measurements are available for each individual, one should resort to techniques that allow borrowing information across subjects, e.g. nonlinear mixed effects models [35–37].

A drawback of the present study is the lack of a standardized follow-up time in the data. We had access to frequent CLM Hgb measurements in a third of the patients for a period of 8 months, which we split into a five months adaptation- and 3 months prediction-period. The number of recordings permitting a longer prediction horizon was low, limiting our assessment of Hgb forecasting performance beyond this point. Further, CLM is currently not widely used in standard care outside the US. The majority of dialysis clinics still rely on less frequent lab Hgb measurements only. Thus, a future study should investigate, if a patient-specific adaptation of the model can be performed using temporally sparse laboratory Hgb data.

Lastly, although erythropoietin is the key hormone driving erythropoiesis, the availability of iron influences bone marrow reaction, which itself is severely impaired by an absolute or functional iron deficiency. While iron homeostasis has not been explicitly incorporated into the presented model, the influence of iron is indirectly accounted for in the model components reflecting the effect of erythropoietin on RBC progenitor cells (which is adapted for each individual).

In practice, clinical anemia therapy is regularly guided by clinical acumen and / or predefined treatment algorithms. However, the long delay between ESA administration and the manifestation of its treatment effects in the form of altered Hgb levels makes reliable treatment forecasts and the design of optimal personalized administration regimens infeasible in daily clinical practice. A better understanding of the genesis of anemia is essential to improve therapy recommendations and guidelines. We have shown that our model can play a crucial role in distinguishing different reasons for a low or non-responsiveness to EPO. The main driver of EPO hypo-responsiveness can be either a severely shortened RBC lifespan, impaired bone-marrow reaction, a very low endogenous EPO production, a short half-life of the drug itself or any combination thereof [25, 32, 38]. In each of these cases, patients will benefit tremendously from a personalized treatment regimen that is adjusted for the specific individual circumstances. Most importantly, numerous administration schemes can be tested for their safety and efficacy in-silico using the proposed model before a specific ESA regimen is clinically evaluated.

As a next step, we will test the predictive performance of the presented model in a larger population. By enrolling virtual and real patients in a large-scale clinical study, model parameters can be correlated to clinical quantities, such as inflammation, fluid status, and others, as well as demographic and anthropomorphic characteristics. The proposed adaptation method readily lends itself to investigate differences in the genesis of renal anemia between e.g. race, gender or age. Moreover, the progress of the underlying causes of the renal anemia in individual patients as predicted by the model will be rigorously assessed in follow-up treatments over several months and years. The model will be re-adapted accordingly so that temporal changes in its parameters represent a dynamic footprint of an individual patient’s status. This will allow us to use the proposed model as a research and diagnostic tool.

## Supporting information

S1 Figs Comparison of model simulations and empirical data. Pre-dialysis Hgb measurements (magenta) and model output (blue) during the model adaptation period (yellow area) and prediction period (purple area). Green bars represent the administered ESA doses.
(ZIP)
