# Supplementary figures and images for: Prediction of hemoglobin levels in individual hemodialysis patients by means of a mathematical model of erythropoiesis
Source: PLoS One. 2018 Apr 18;13(4):e0195918. doi: 10.1371/journal.pone.0195918 (PMC5905967; doi:10.1371/journal.pone.0195918)

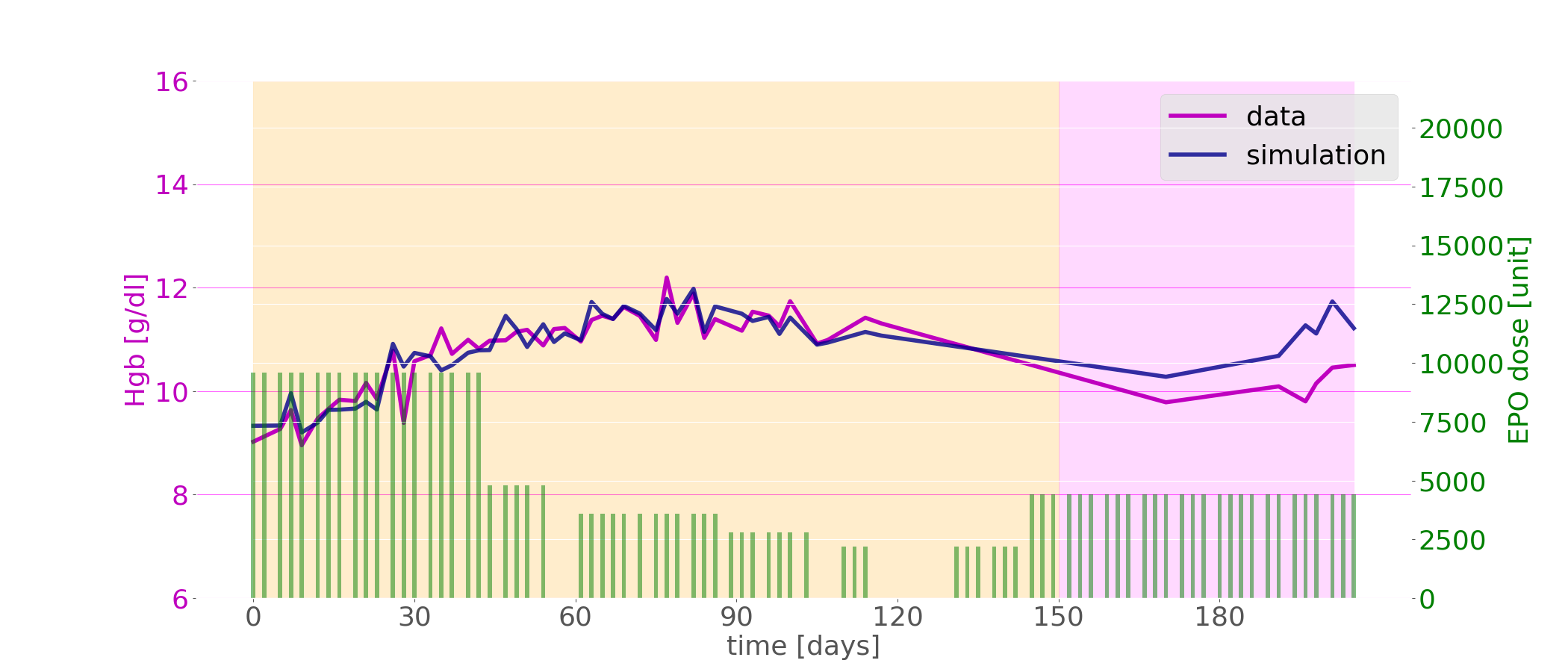

Supplement: S1 Figs — Pre-dialysis Hgb measurements (magenta) and model output (blue) during the model adaptation period (yellow area) and prediction period (purple area). Green bars represent the administered ESA doses. (ZIP) [file pone.0195918.s001.zip › patient_100001.png]

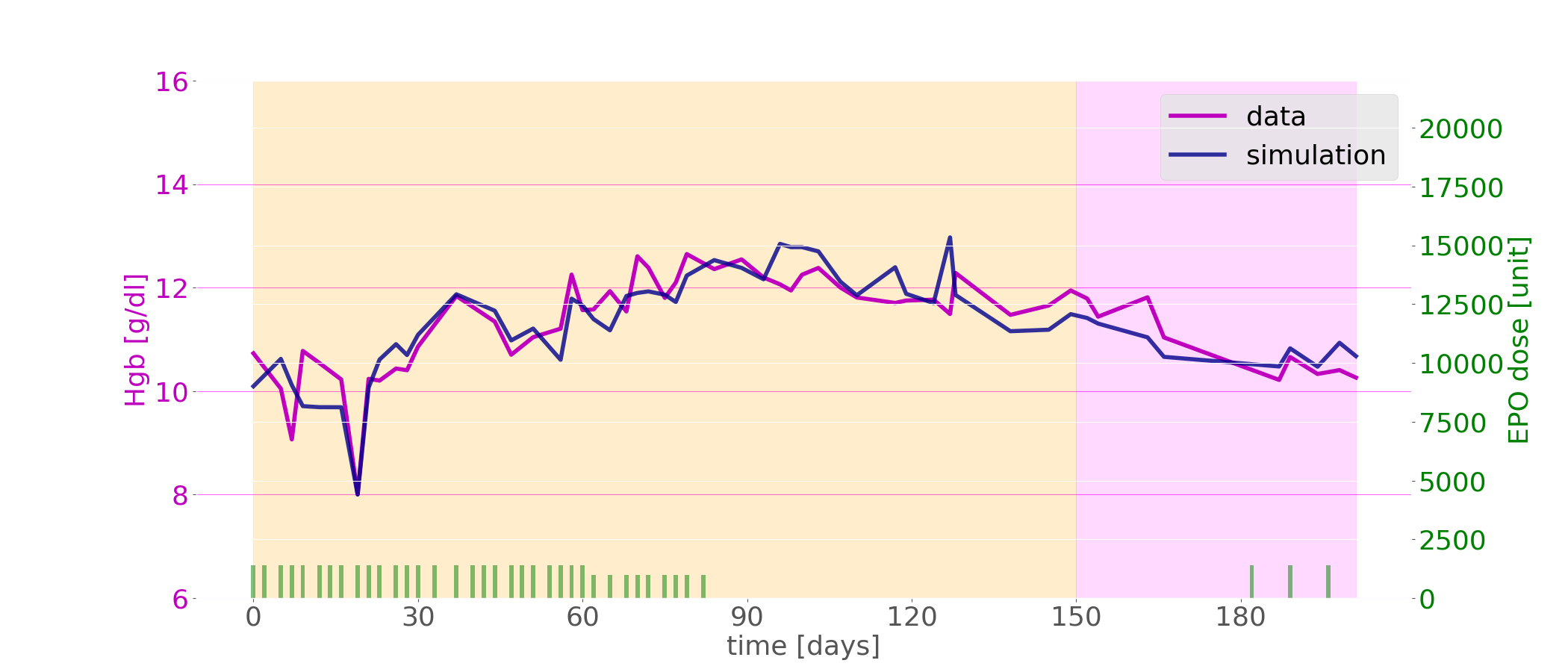

Supplement: S1 Figs — Pre-dialysis Hgb measurements (magenta) and model output (blue) during the model adaptation period (yellow area) and prediction period (purple area). Green bars represent the administered ESA doses. (ZIP) [file pone.0195918.s001.zip › patient_100002.png]

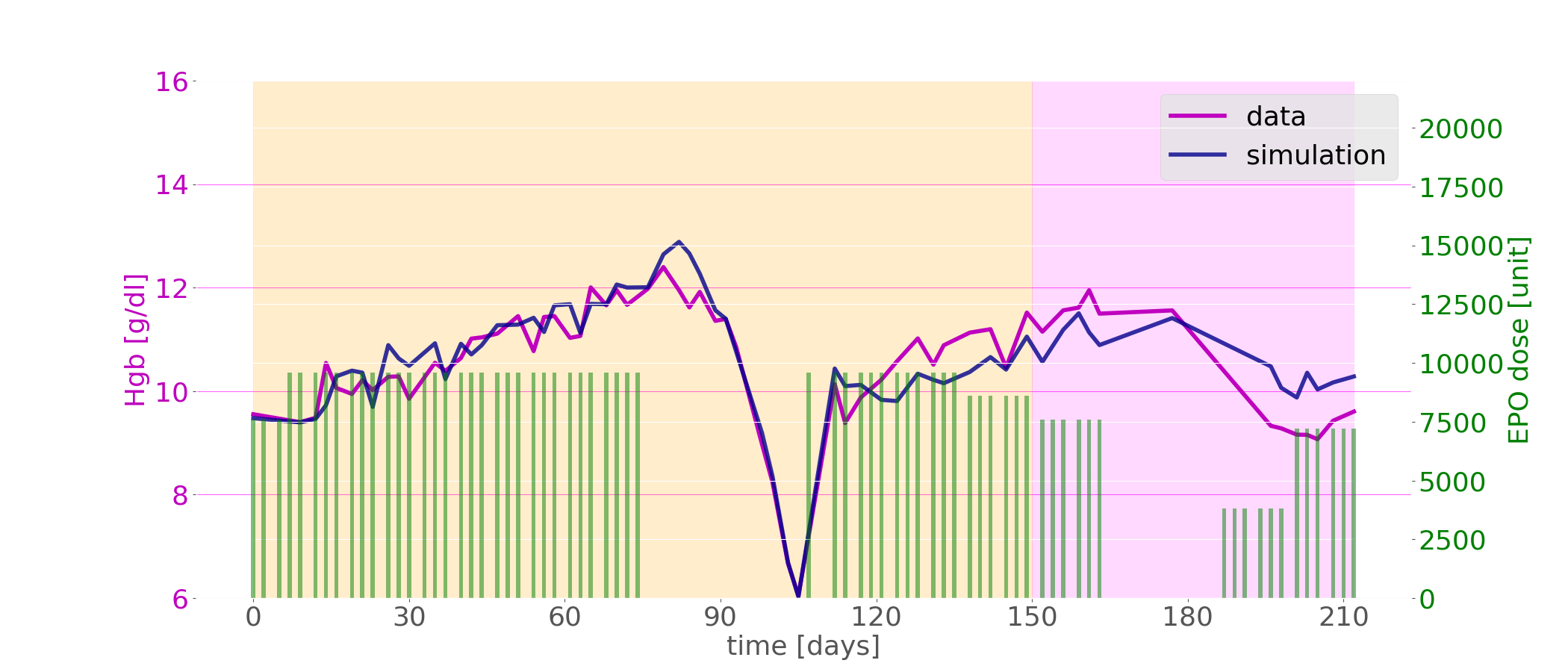

Supplement: S1 Figs — Pre-dialysis Hgb measurements (magenta) and model output (blue) during the model adaptation period (yellow area) and prediction period (purple area). Green bars represent the administered ESA doses. (ZIP) [file pone.0195918.s001.zip › patient_100003.png]

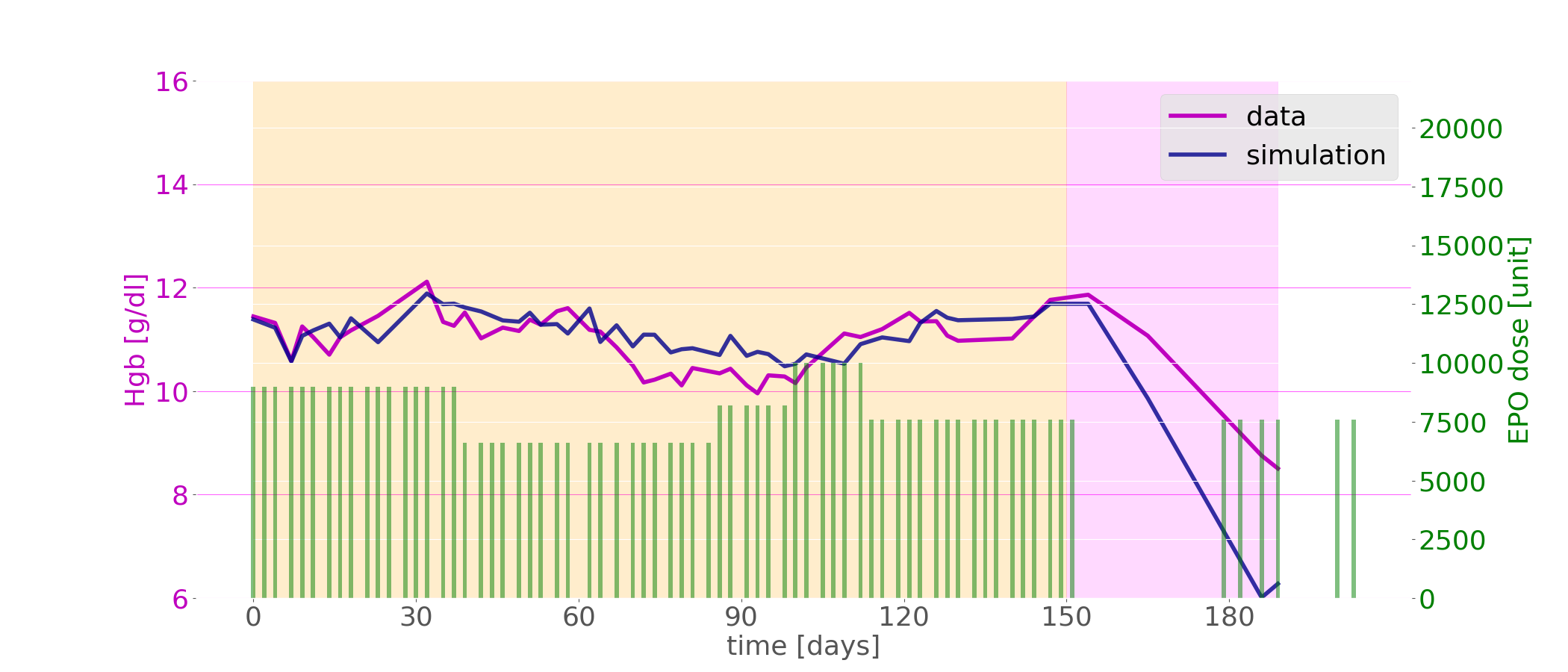

Supplement: S1 Figs — Pre-dialysis Hgb measurements (magenta) and model output (blue) during the model adaptation period (yellow area) and prediction period (purple area). Green bars represent the administered ESA doses. (ZIP) [file pone.0195918.s001.zip › patient_100004.png]

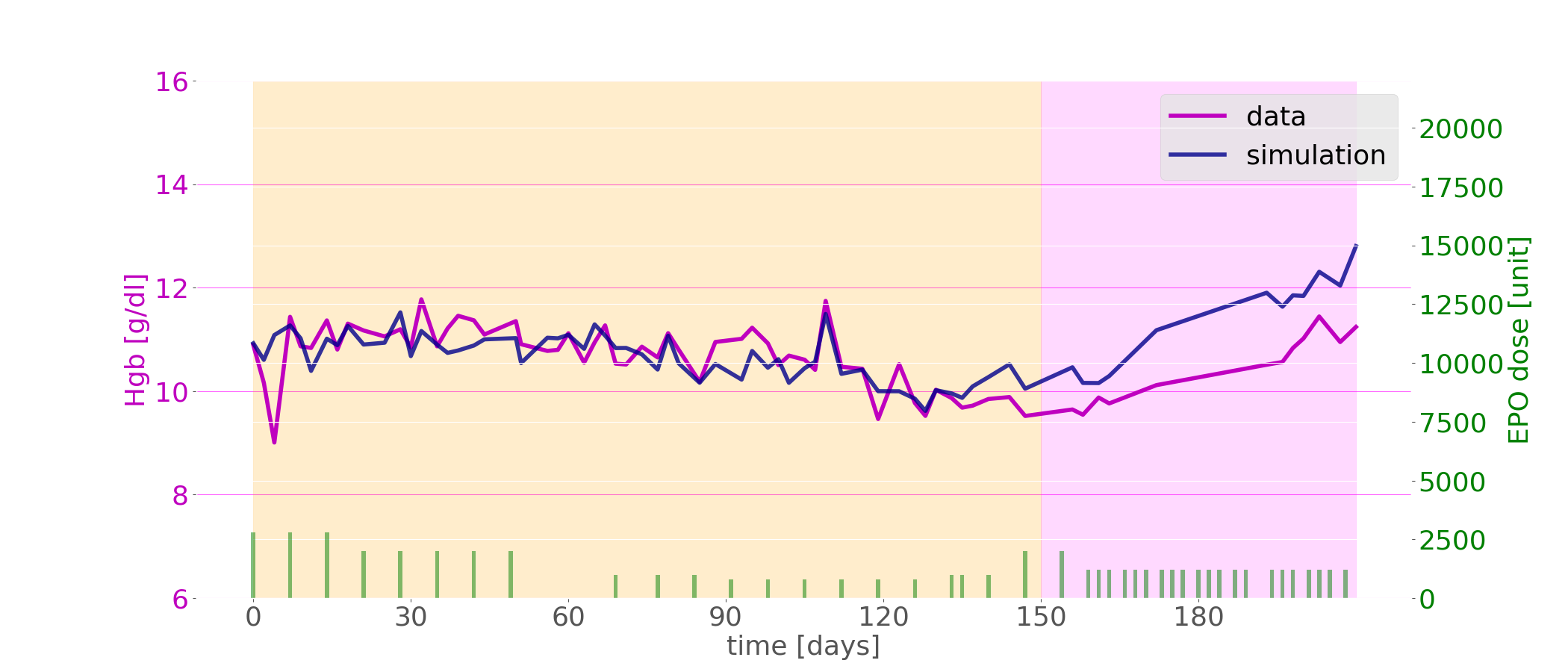

Supplement: S1 Figs — Pre-dialysis Hgb measurements (magenta) and model output (blue) during the model adaptation period (yellow area) and prediction period (purple area). Green bars represent the administered ESA doses. (ZIP) [file pone.0195918.s001.zip › patient_100005.png]

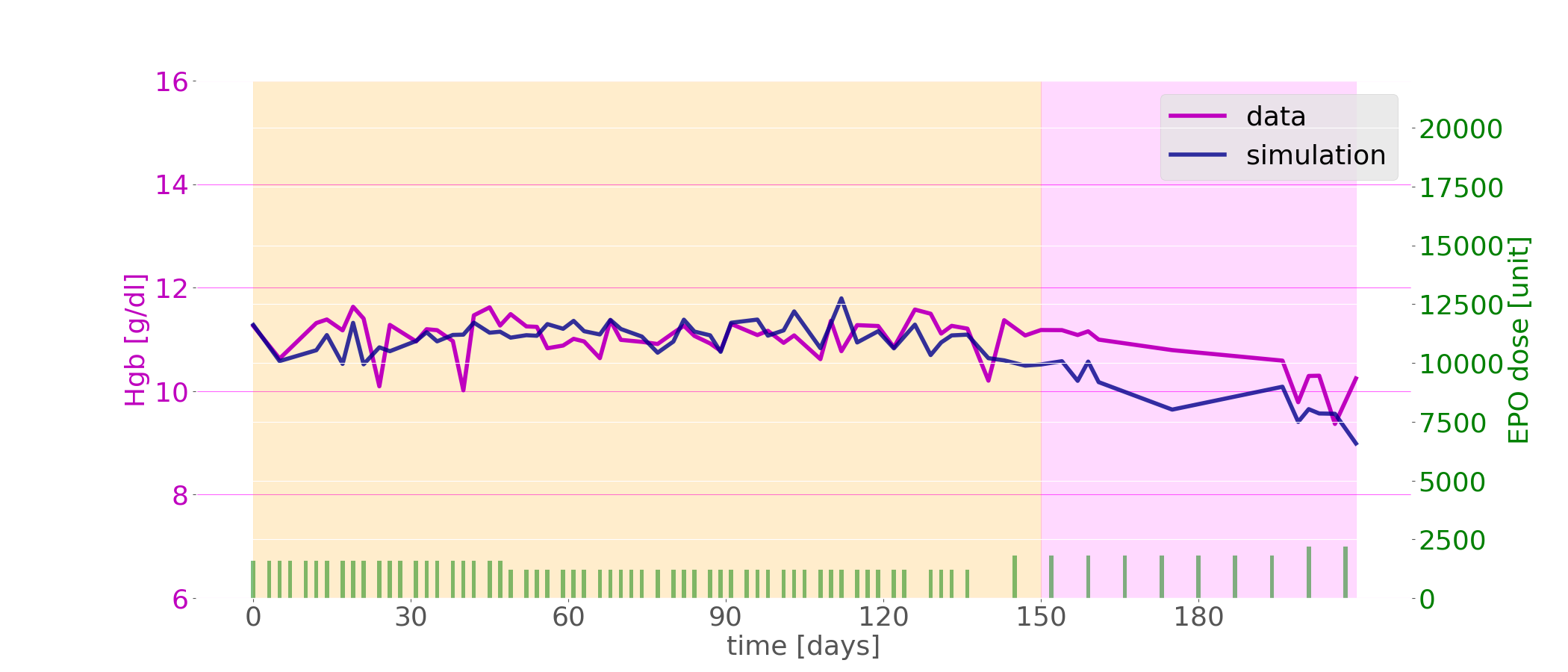

Supplement: S1 Figs — Pre-dialysis Hgb measurements (magenta) and model output (blue) during the model adaptation period (yellow area) and prediction period (purple area). Green bars represent the administered ESA doses. (ZIP) [file pone.0195918.s001.zip › patient_100006.png]

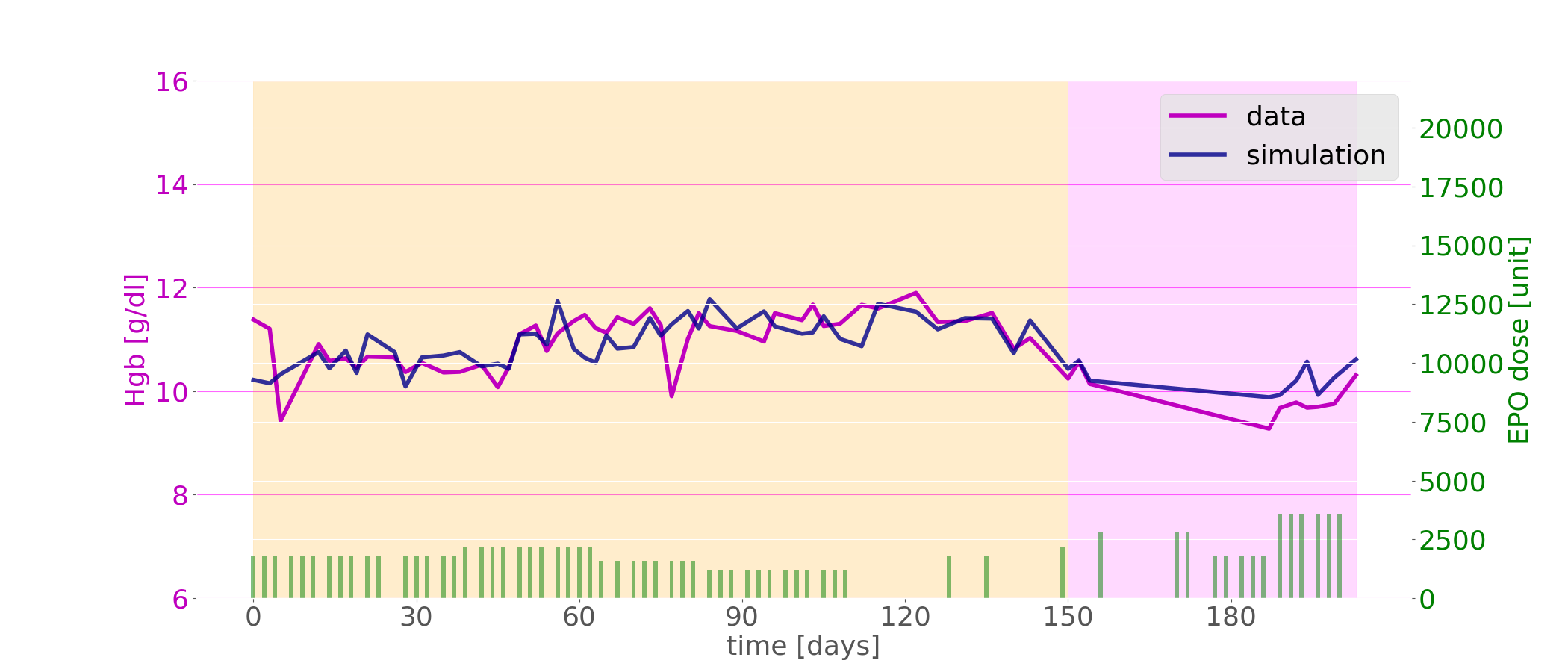

Supplement: S1 Figs — Pre-dialysis Hgb measurements (magenta) and model output (blue) during the model adaptation period (yellow area) and prediction period (purple area). Green bars represent the administered ESA doses. (ZIP) [file pone.0195918.s001.zip › patient_100007.png]

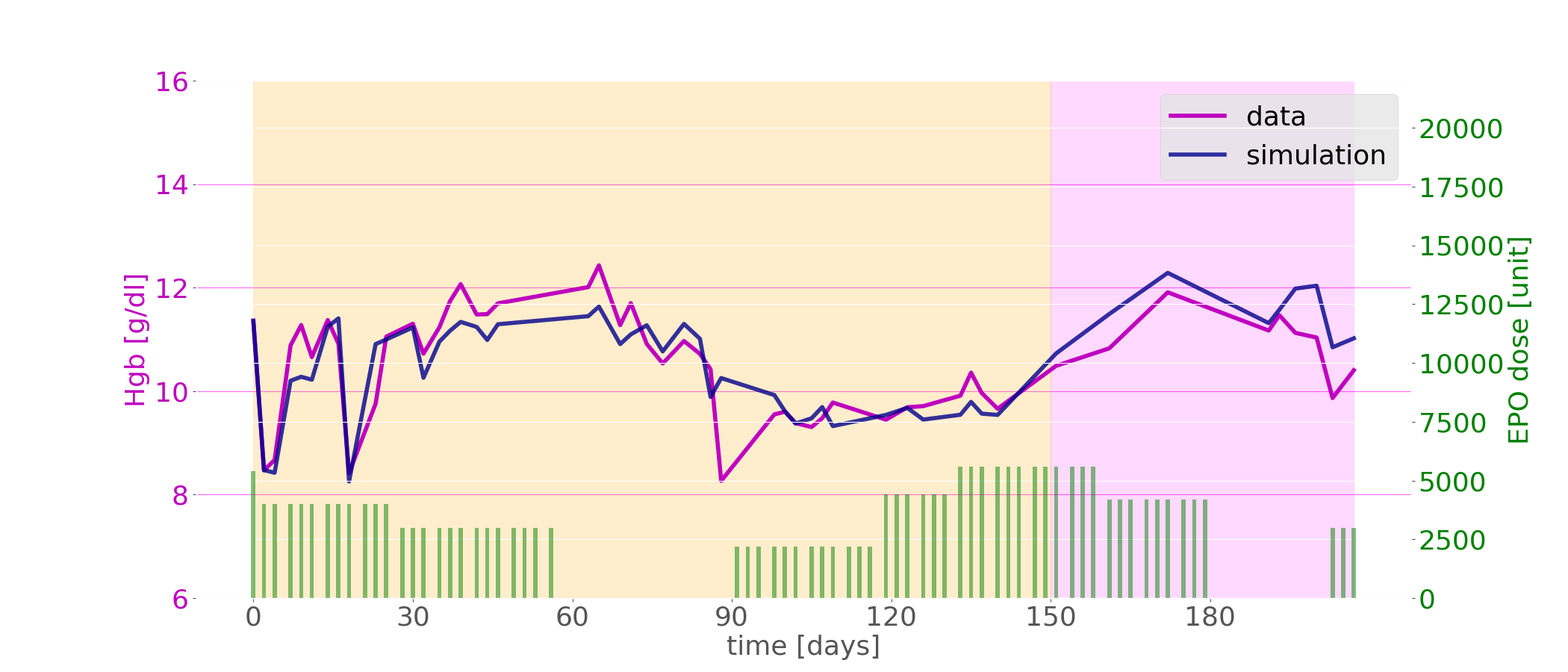

Supplement: S1 Figs — Pre-dialysis Hgb measurements (magenta) and model output (blue) during the model adaptation period (yellow area) and prediction period (purple area). Green bars represent the administered ESA doses. (ZIP) [file pone.0195918.s001.zip › patient_100008.png]

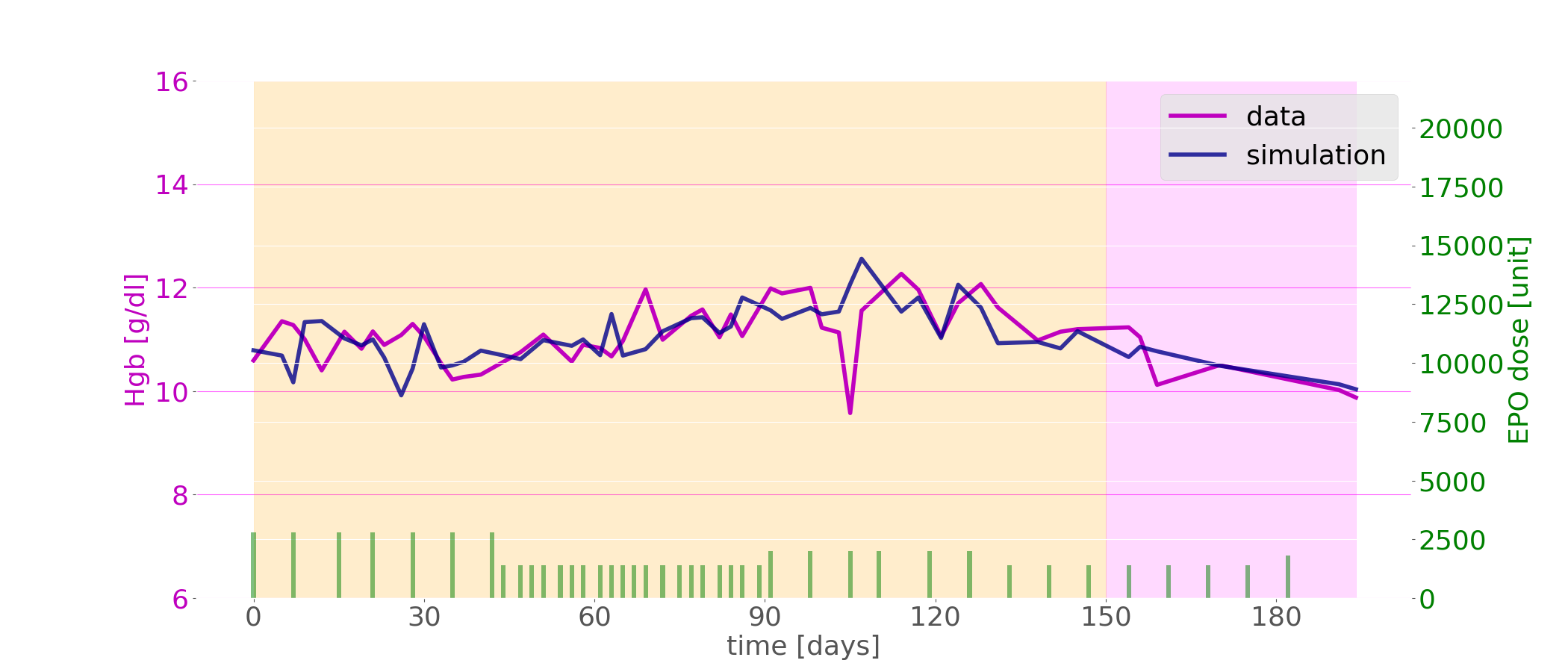

Supplement: S1 Figs — Pre-dialysis Hgb measurements (magenta) and model output (blue) during the model adaptation period (yellow area) and prediction period (purple area). Green bars represent the administered ESA doses. (ZIP) [file pone.0195918.s001.zip › patient_100009.png]

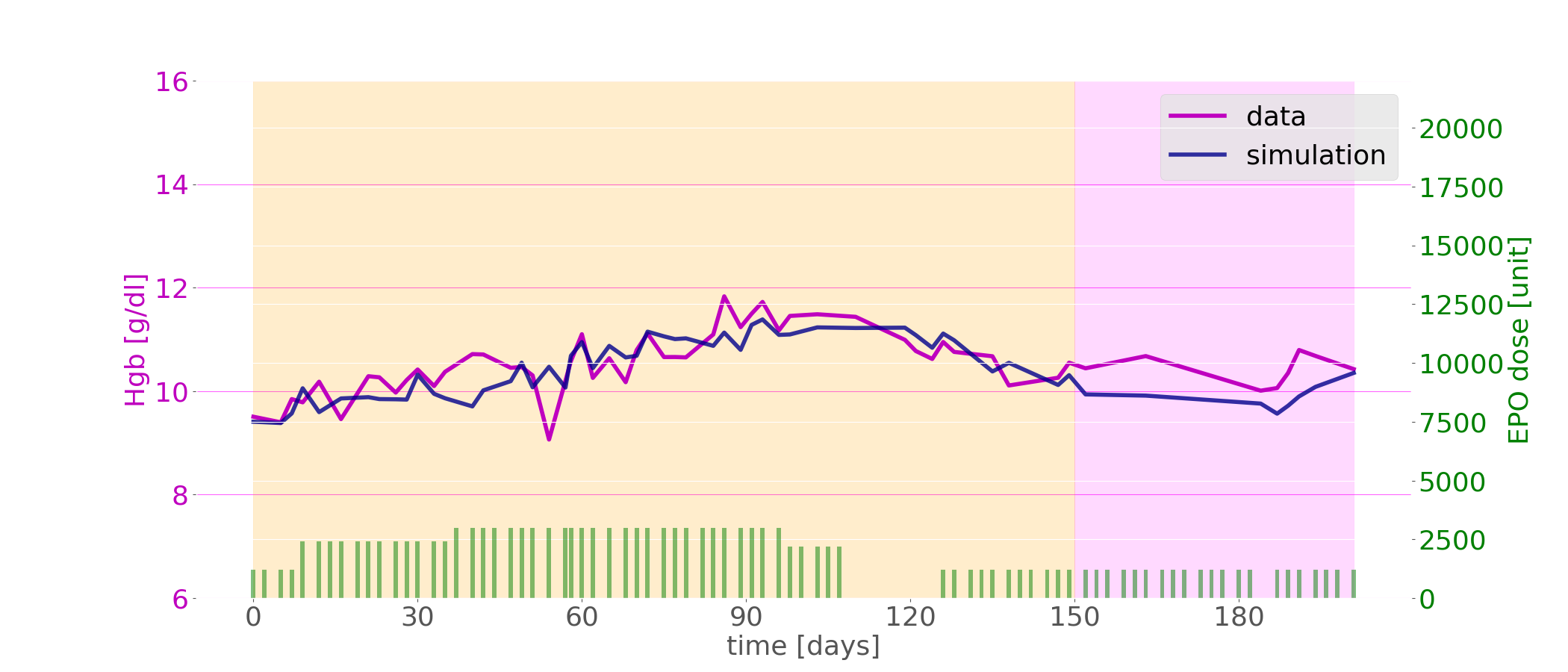

Supplement: S1 Figs — Pre-dialysis Hgb measurements (magenta) and model output (blue) during the model adaptation period (yellow area) and prediction period (purple area). Green bars represent the administered ESA doses. (ZIP) [file pone.0195918.s001.zip › patient_100010.png]

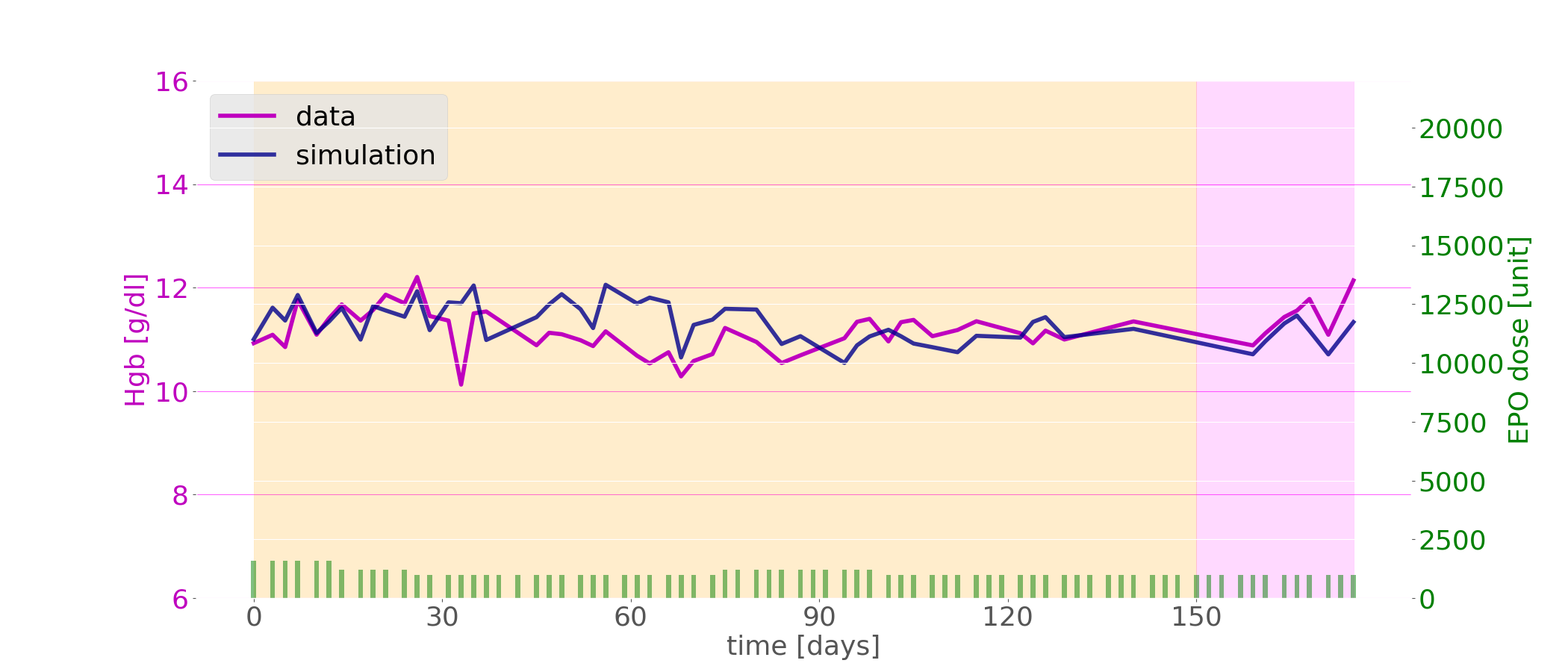

Supplement: S1 Figs — Pre-dialysis Hgb measurements (magenta) and model output (blue) during the model adaptation period (yellow area) and prediction period (purple area). Green bars represent the administered ESA doses. (ZIP) [file pone.0195918.s001.zip › patient_100011.png]

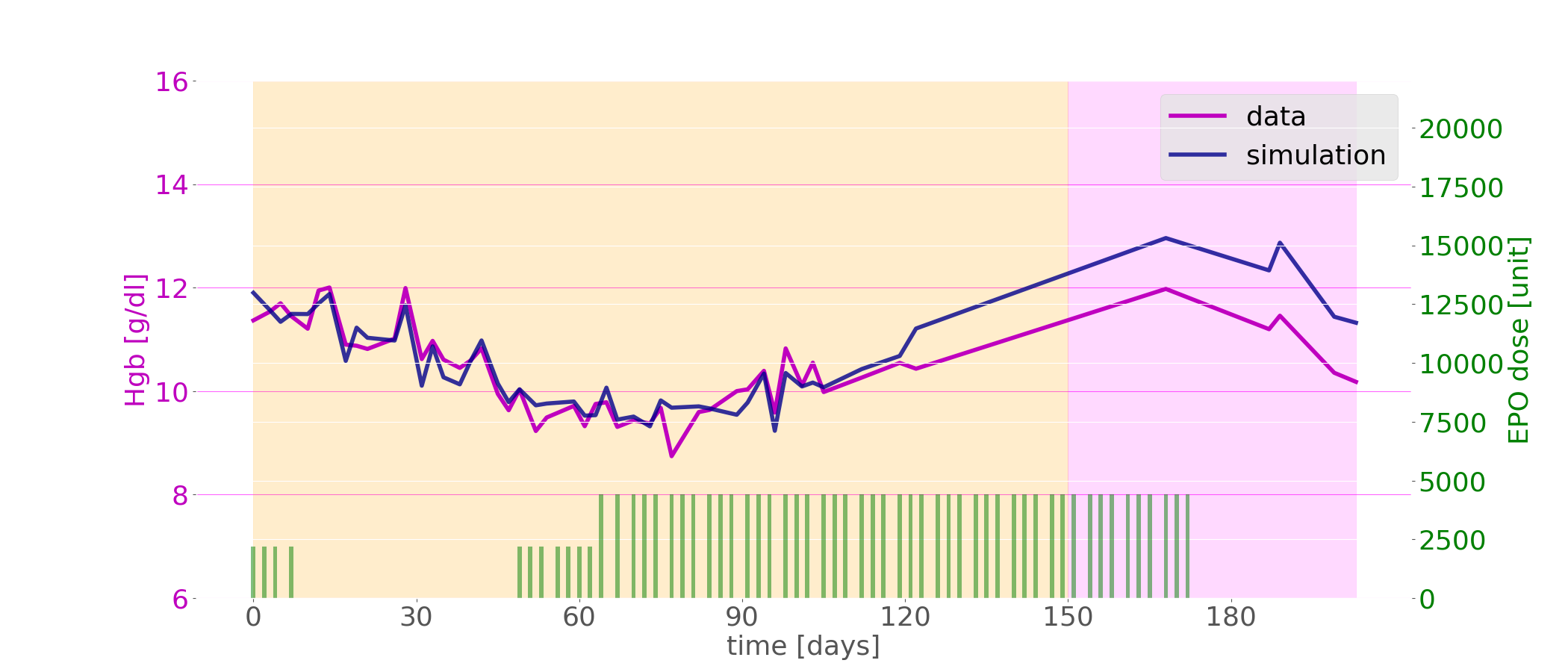

Supplement: S1 Figs — Pre-dialysis Hgb measurements (magenta) and model output (blue) during the model adaptation period (yellow area) and prediction period (purple area). Green bars represent the administered ESA doses. (ZIP) [file pone.0195918.s001.zip › patient_100012.png]

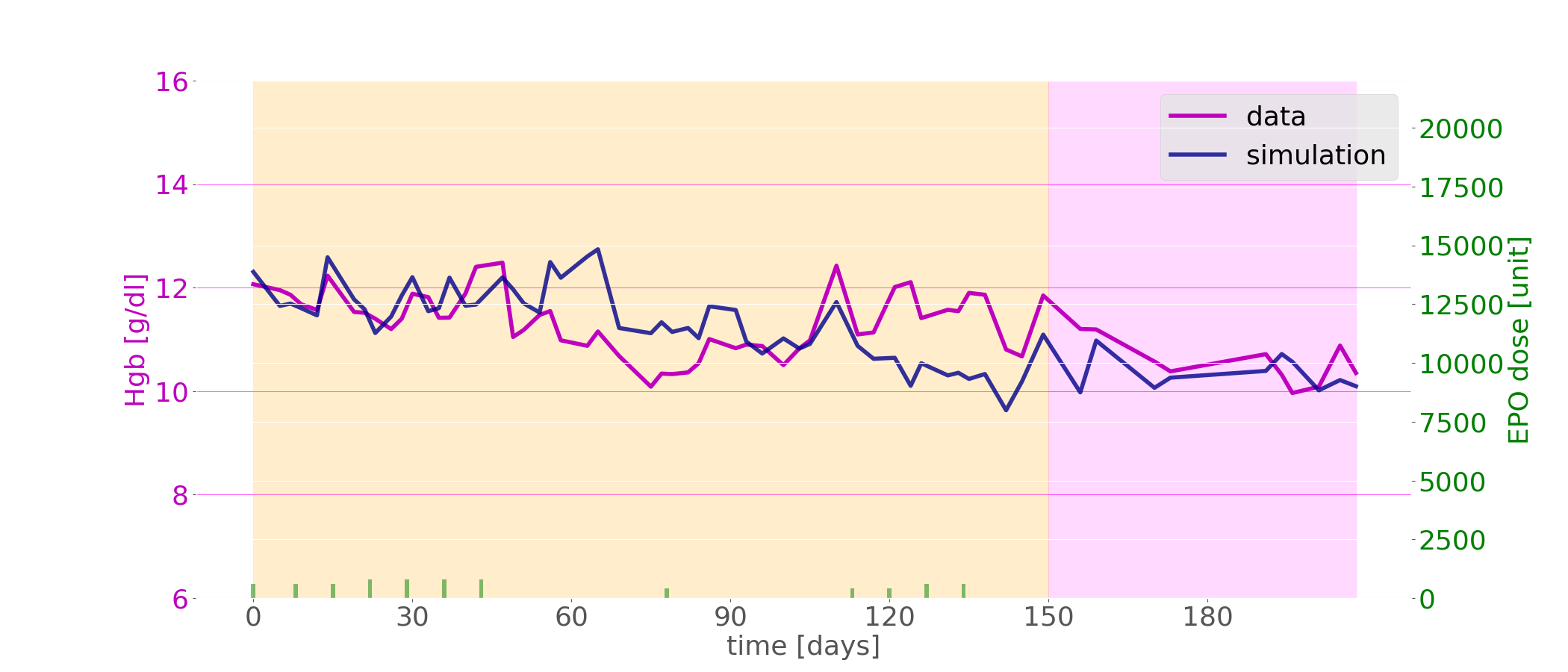

Supplement: S1 Figs — Pre-dialysis Hgb measurements (magenta) and model output (blue) during the model adaptation period (yellow area) and prediction period (purple area). Green bars represent the administered ESA doses. (ZIP) [file pone.0195918.s001.zip › patient_100013.png]

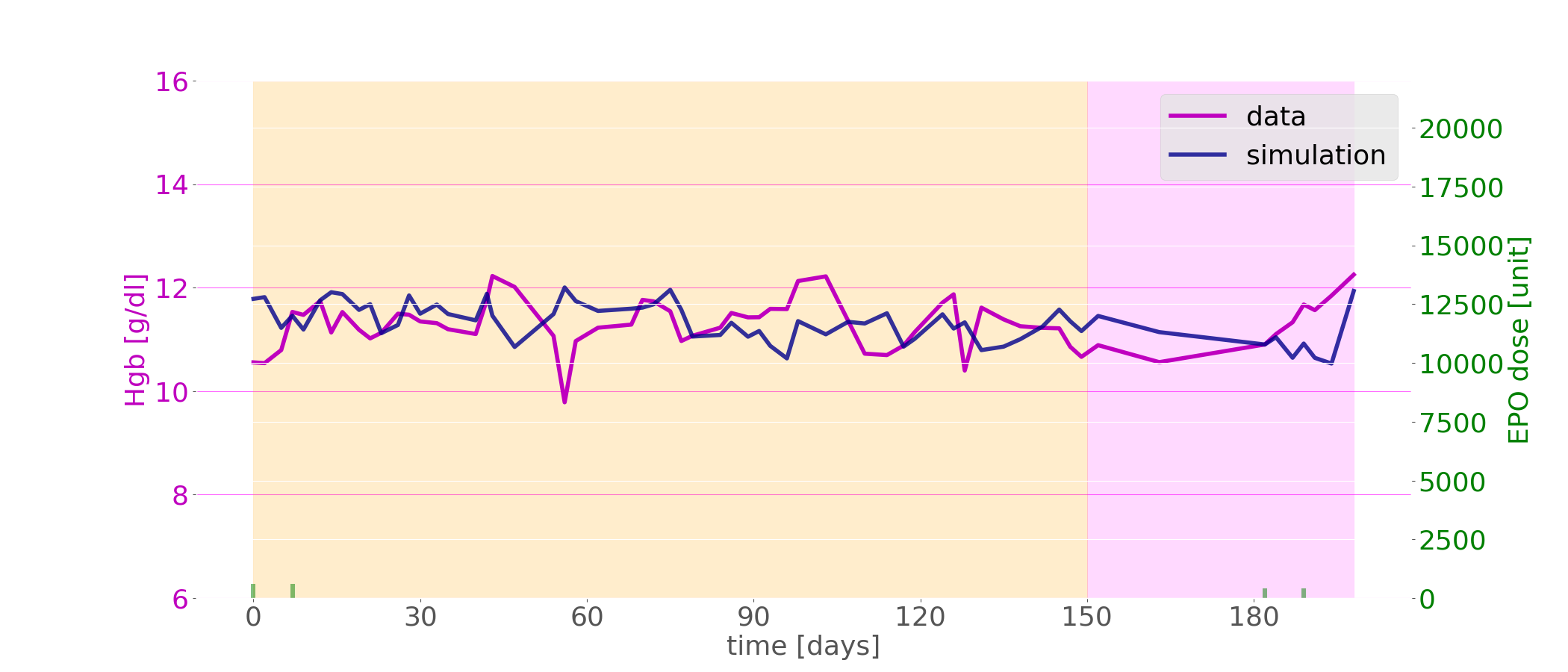

Supplement: S1 Figs — Pre-dialysis Hgb measurements (magenta) and model output (blue) during the model adaptation period (yellow area) and prediction period (purple area). Green bars represent the administered ESA doses. (ZIP) [file pone.0195918.s001.zip › patient_100014.png]

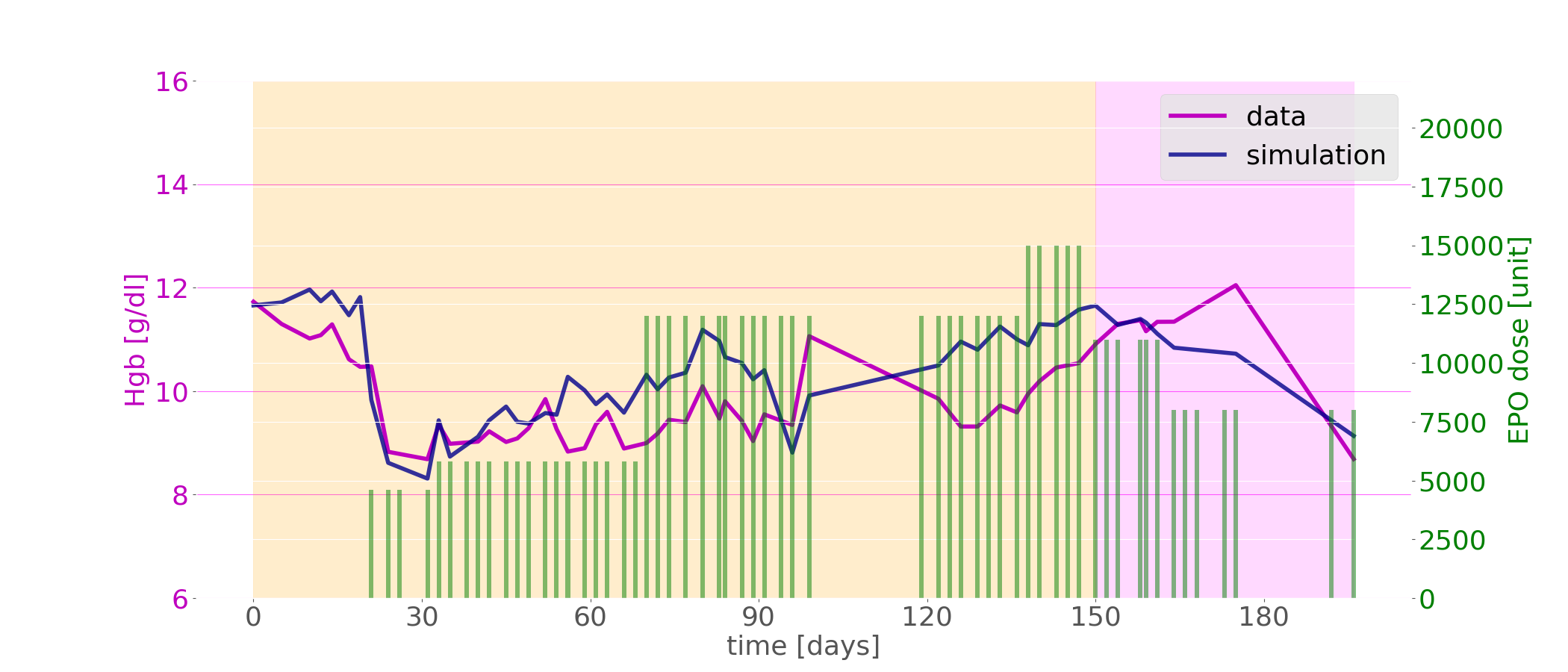

Supplement: S1 Figs — Pre-dialysis Hgb measurements (magenta) and model output (blue) during the model adaptation period (yellow area) and prediction period (purple area). Green bars represent the administered ESA doses. (ZIP) [file pone.0195918.s001.zip › patient_100015.png]

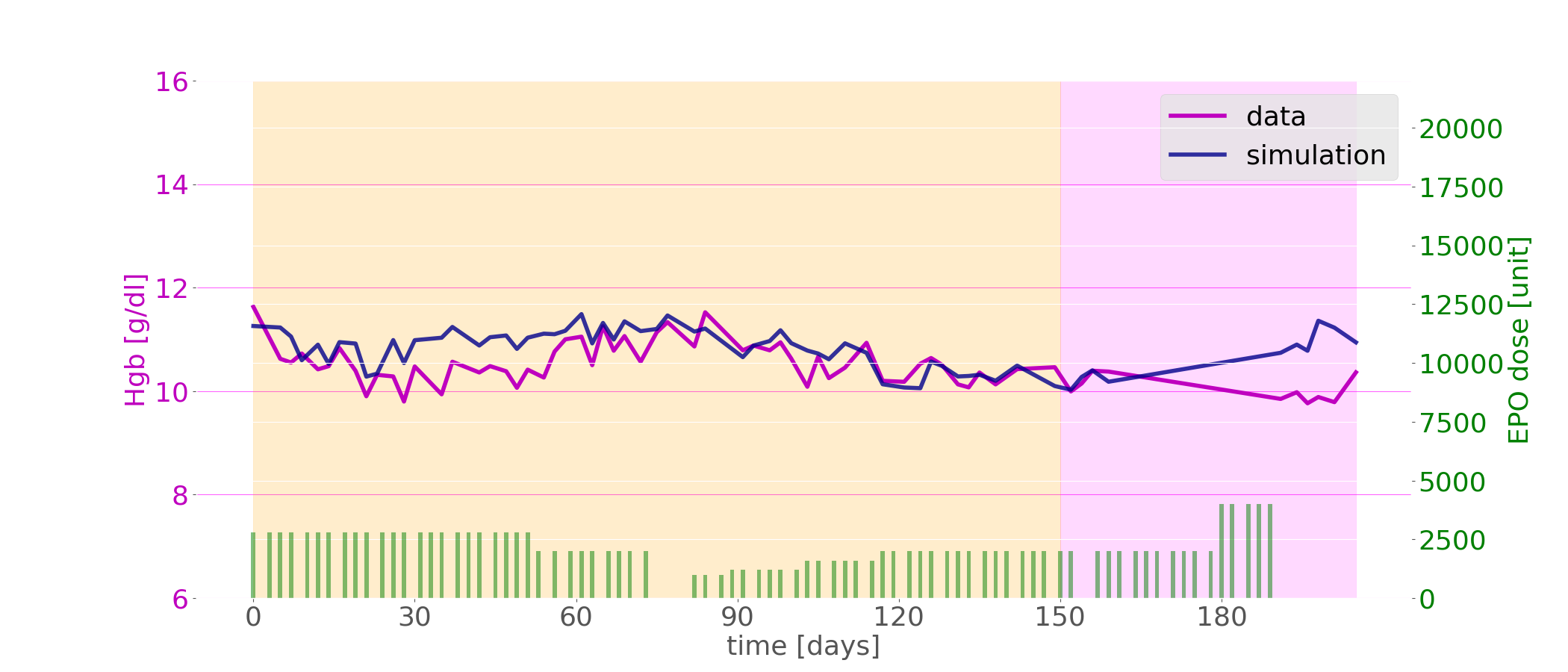

Supplement: S1 Figs — Pre-dialysis Hgb measurements (magenta) and model output (blue) during the model adaptation period (yellow area) and prediction period (purple area). Green bars represent the administered ESA doses. (ZIP) [file pone.0195918.s001.zip › patient_100016.png]

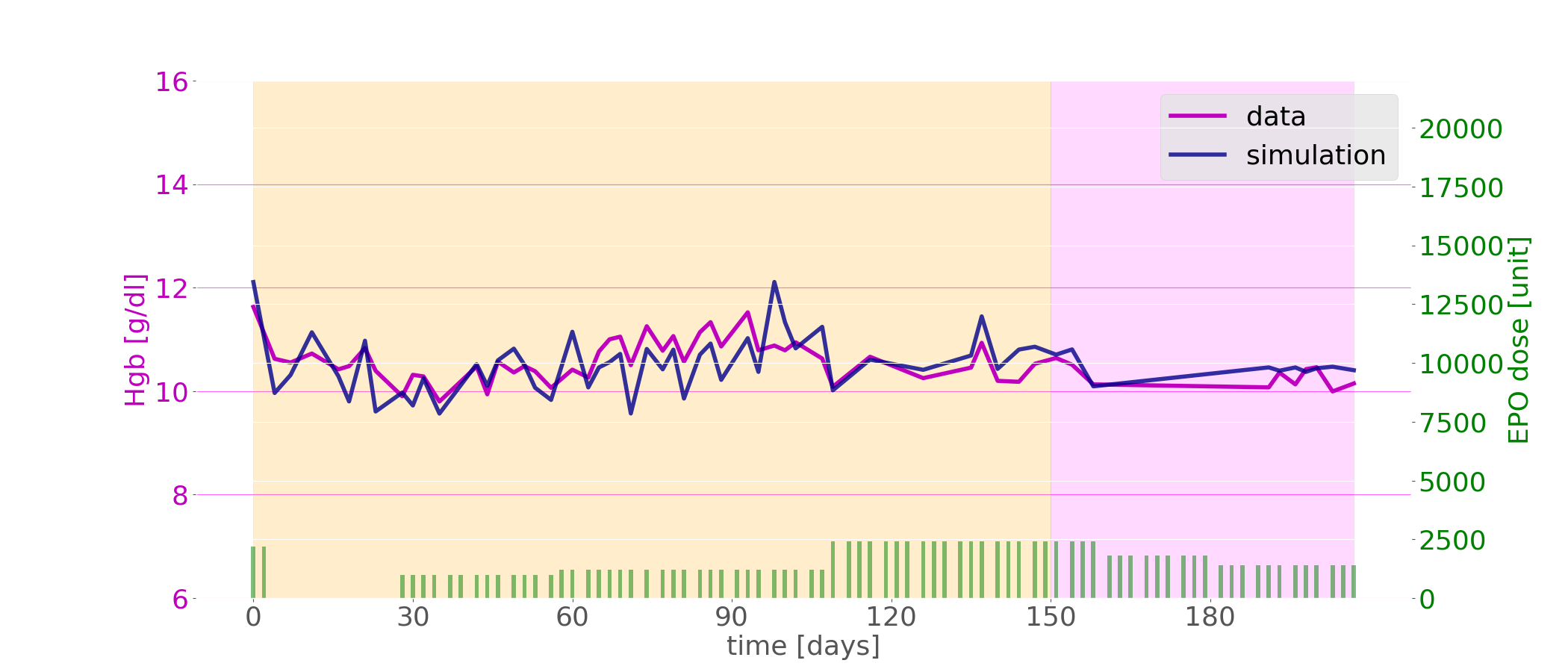

Supplement: S1 Figs — Pre-dialysis Hgb measurements (magenta) and model output (blue) during the model adaptation period (yellow area) and prediction period (purple area). Green bars represent the administered ESA doses. (ZIP) [file pone.0195918.s001.zip › patient_100017.png]

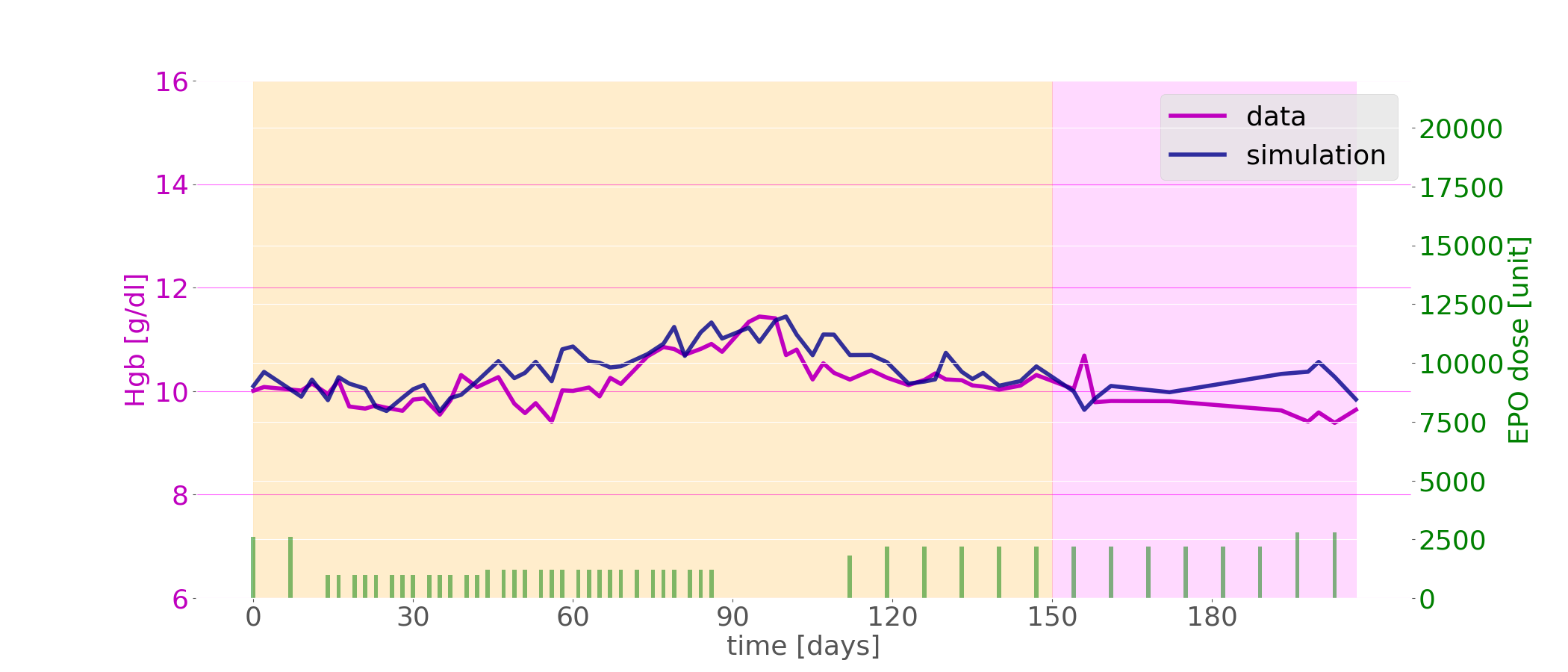

Supplement: S1 Figs — Pre-dialysis Hgb measurements (magenta) and model output (blue) during the model adaptation period (yellow area) and prediction period (purple area). Green bars represent the administered ESA doses. (ZIP) [file pone.0195918.s001.zip › patient_100018.png]

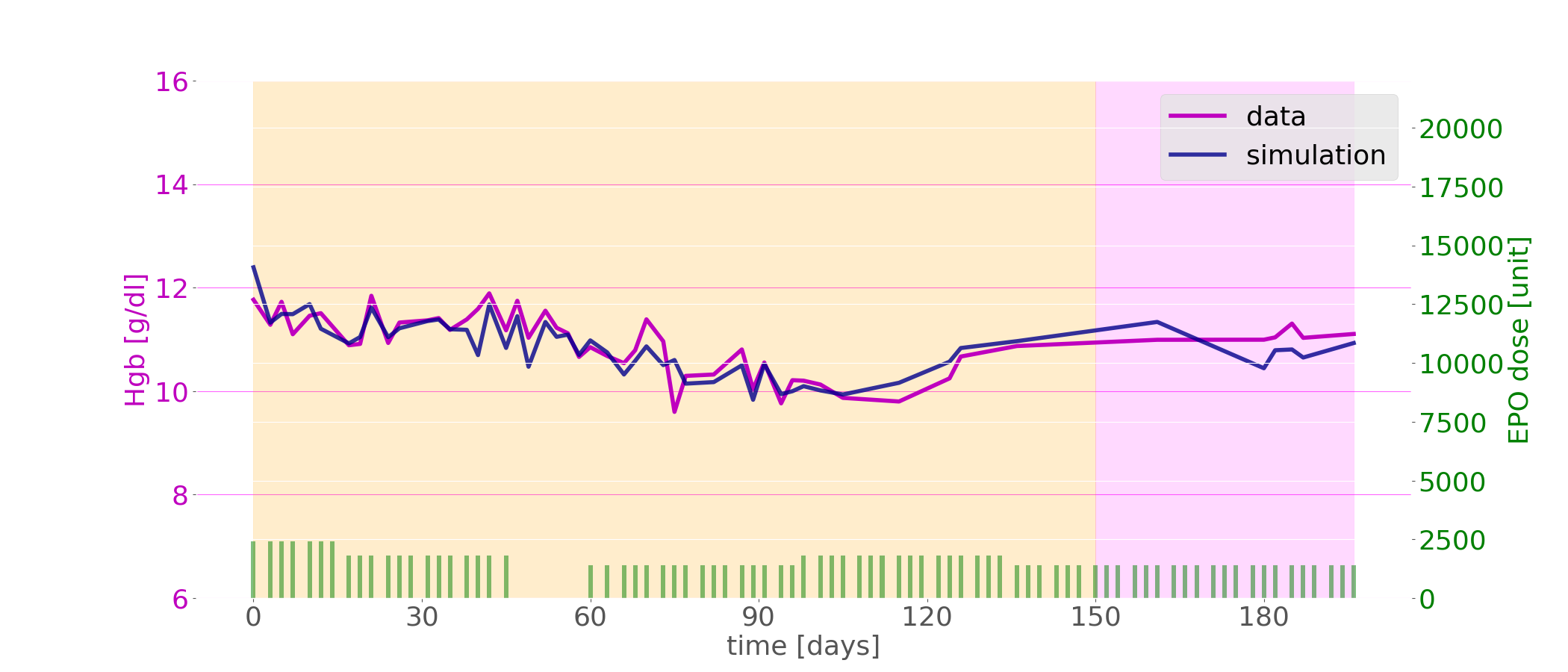

Supplement: S1 Figs — Pre-dialysis Hgb measurements (magenta) and model output (blue) during the model adaptation period (yellow area) and prediction period (purple area). Green bars represent the administered ESA doses. (ZIP) [file pone.0195918.s001.zip › patient_100019.png]

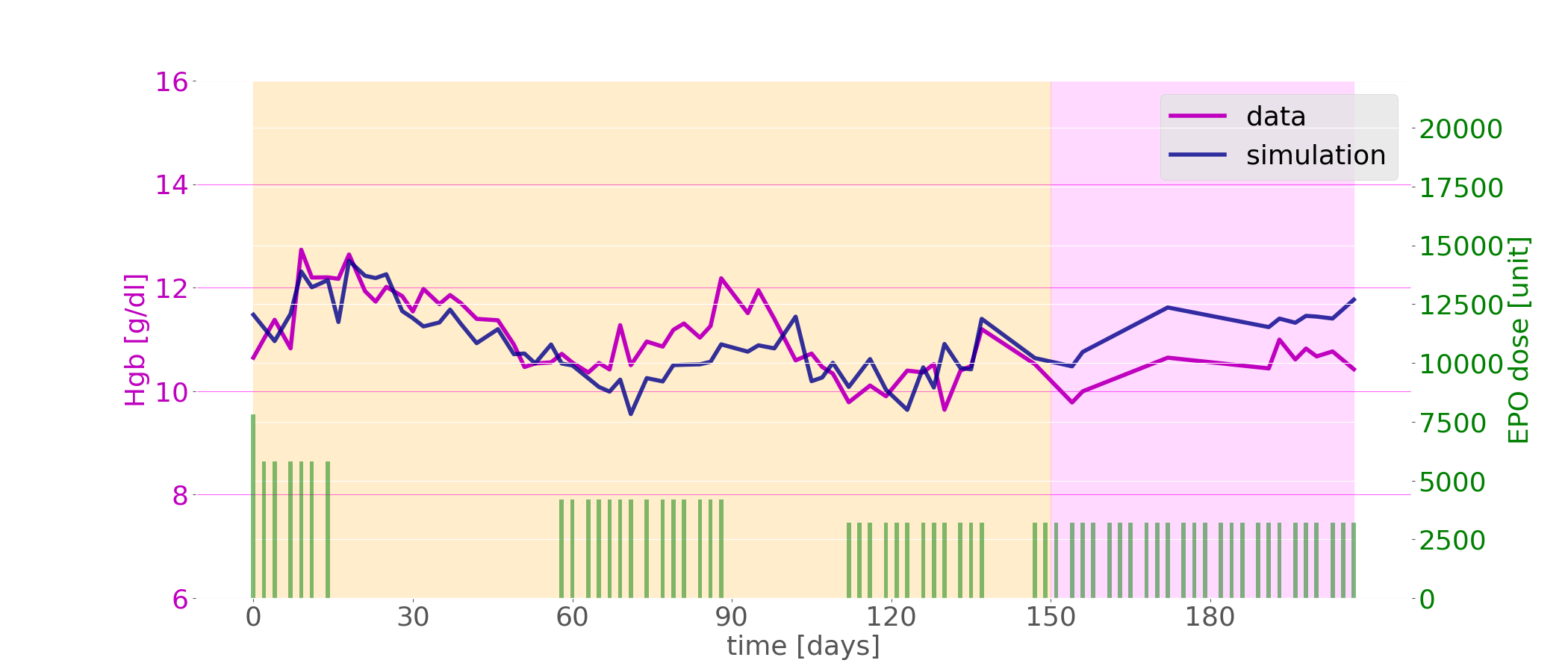

Supplement: S1 Figs — Pre-dialysis Hgb measurements (magenta) and model output (blue) during the model adaptation period (yellow area) and prediction period (purple area). Green bars represent the administered ESA doses. (ZIP) [file pone.0195918.s001.zip › patient_100020.png]

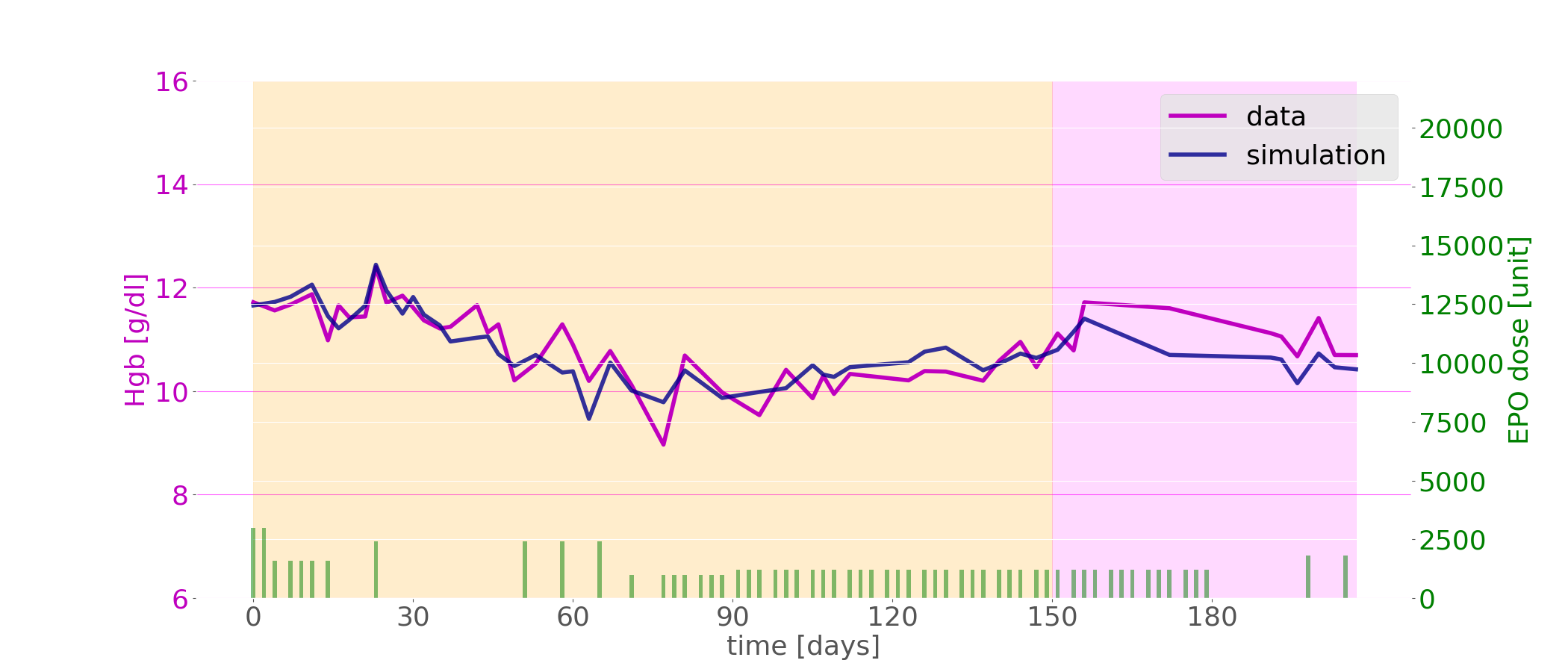

Supplement: S1 Figs — Pre-dialysis Hgb measurements (magenta) and model output (blue) during the model adaptation period (yellow area) and prediction period (purple area). Green bars represent the administered ESA doses. (ZIP) [file pone.0195918.s001.zip › patient_100021.png]

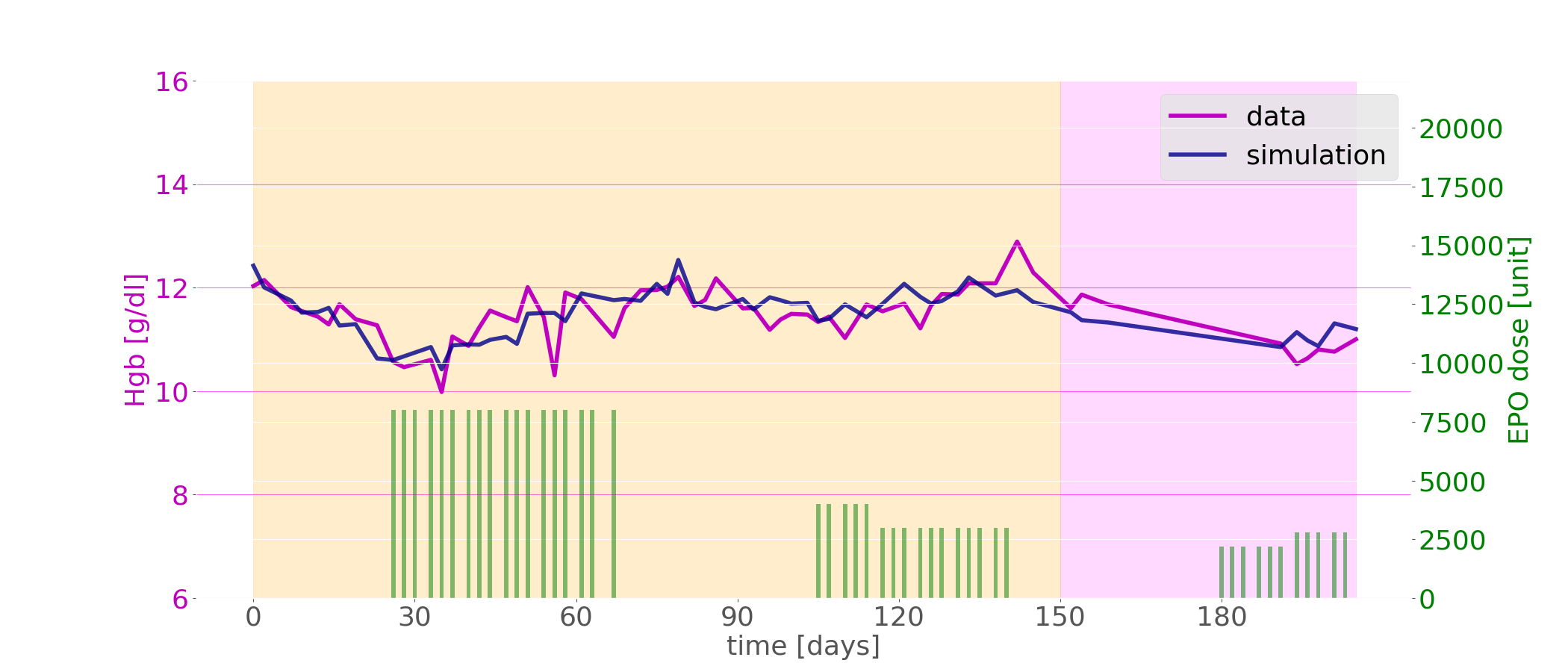

Supplement: S1 Figs — Pre-dialysis Hgb measurements (magenta) and model output (blue) during the model adaptation period (yellow area) and prediction period (purple area). Green bars represent the administered ESA doses. (ZIP) [file pone.0195918.s001.zip › patient_100022.png]

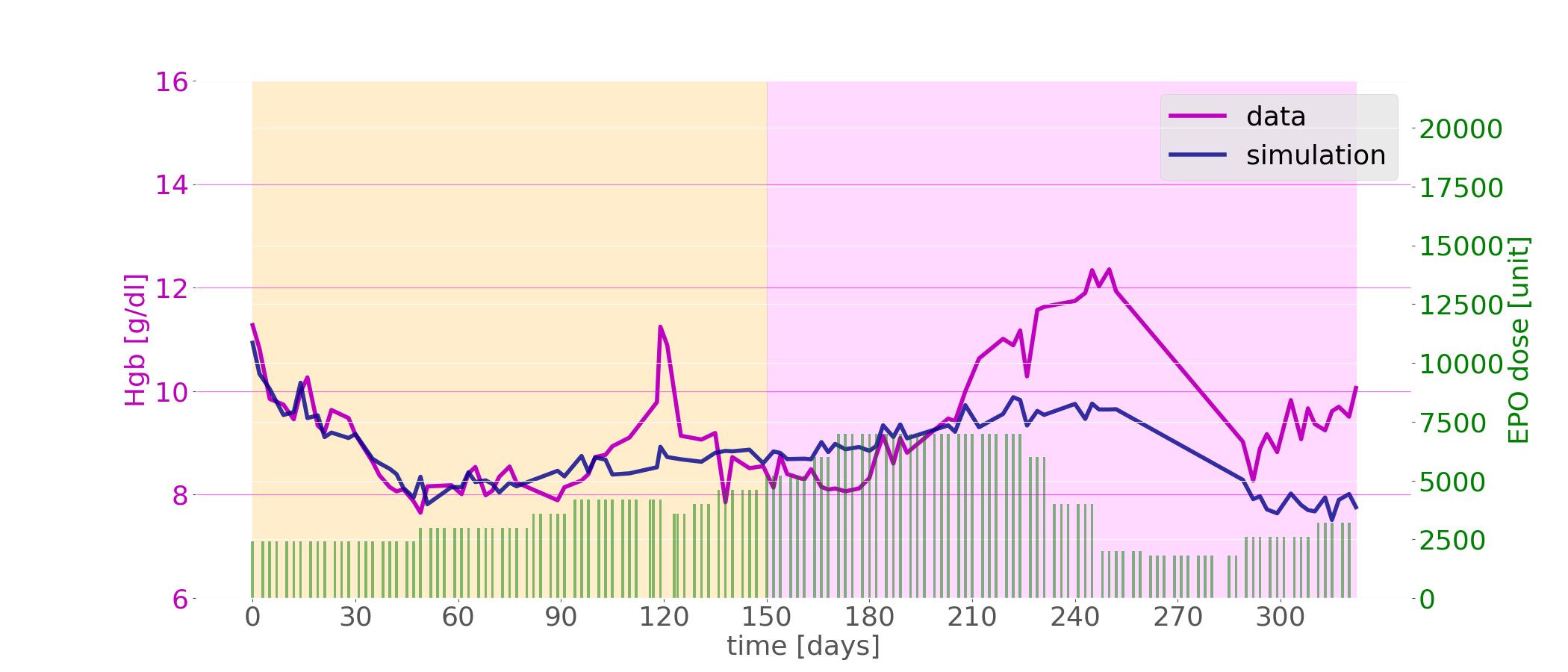

Supplement: S1 Figs — Pre-dialysis Hgb measurements (magenta) and model output (blue) during the model adaptation period (yellow area) and prediction period (purple area). Green bars represent the administered ESA doses. (ZIP) [file pone.0195918.s001.zip › patient_100023.png]

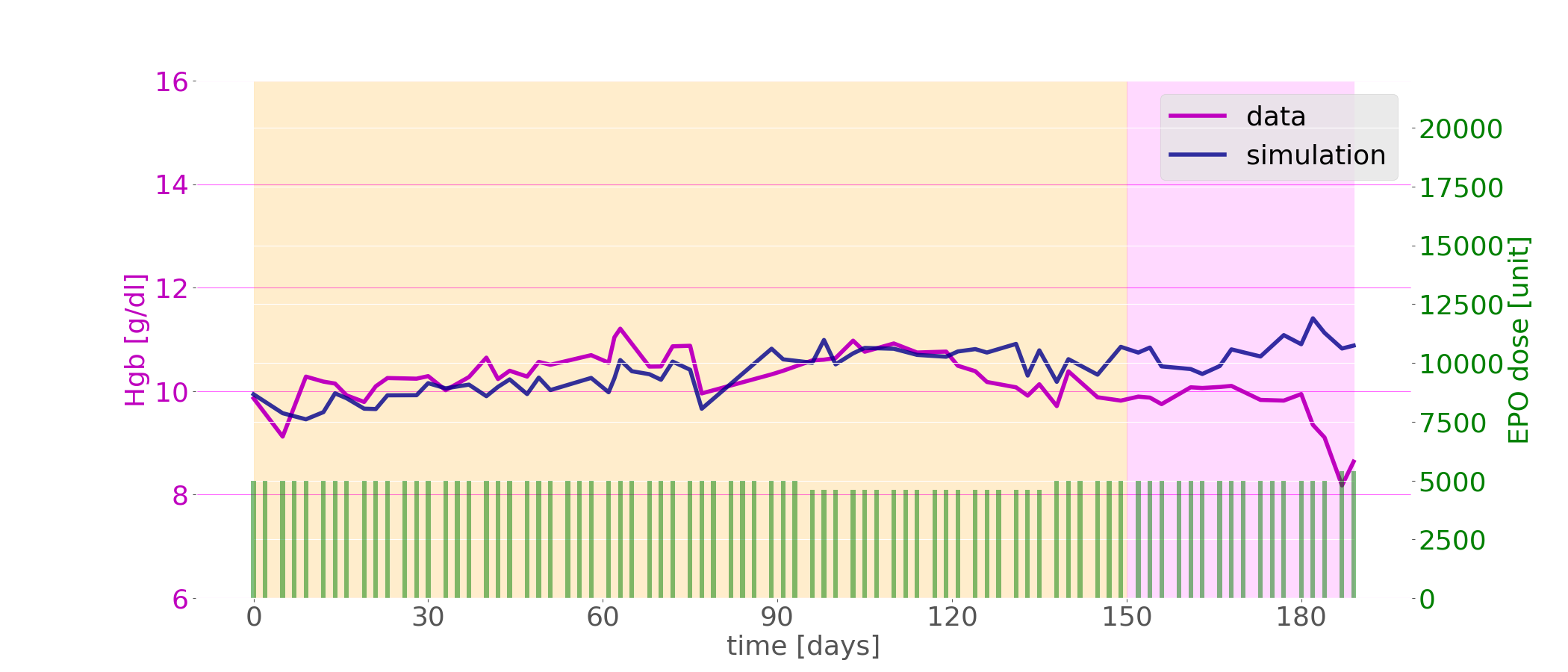

Supplement: S1 Figs — Pre-dialysis Hgb measurements (magenta) and model output (blue) during the model adaptation period (yellow area) and prediction period (purple area). Green bars represent the administered ESA doses. (ZIP) [file pone.0195918.s001.zip › patient_100024.png]

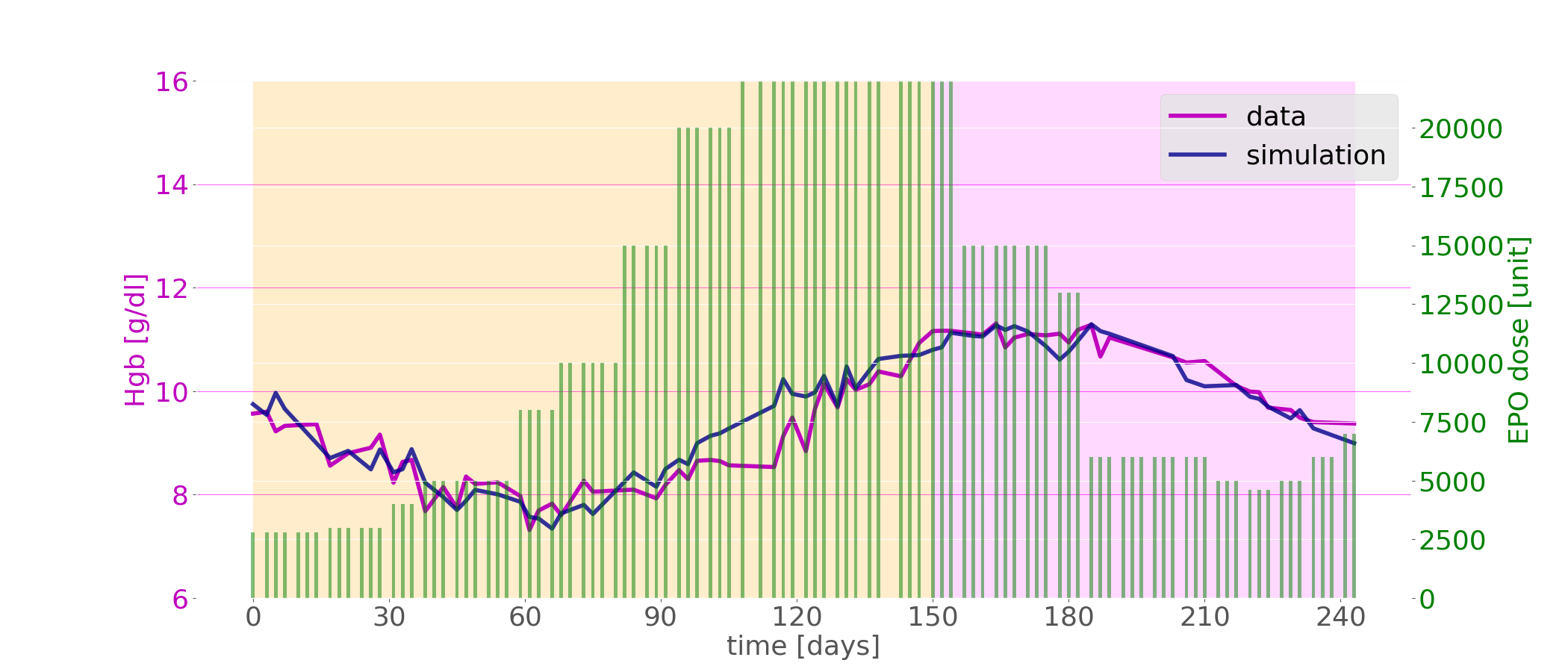

Supplement: S1 Figs — Pre-dialysis Hgb measurements (magenta) and model output (blue) during the model adaptation period (yellow area) and prediction period (purple area). Green bars represent the administered ESA doses. (ZIP) [file pone.0195918.s001.zip › patient_100025.png]

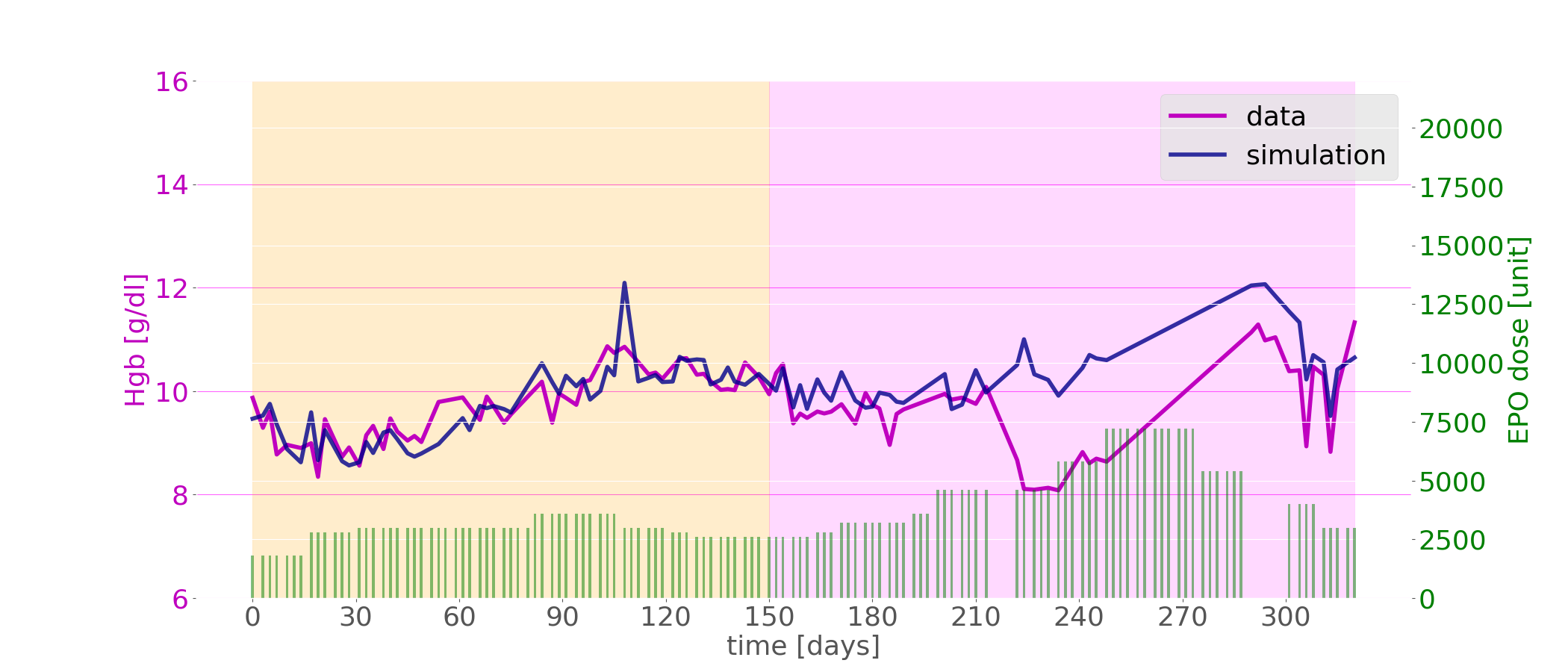

Supplement: S1 Figs — Pre-dialysis Hgb measurements (magenta) and model output (blue) during the model adaptation period (yellow area) and prediction period (purple area). Green bars represent the administered ESA doses. (ZIP) [file pone.0195918.s001.zip › patient_100026.png]

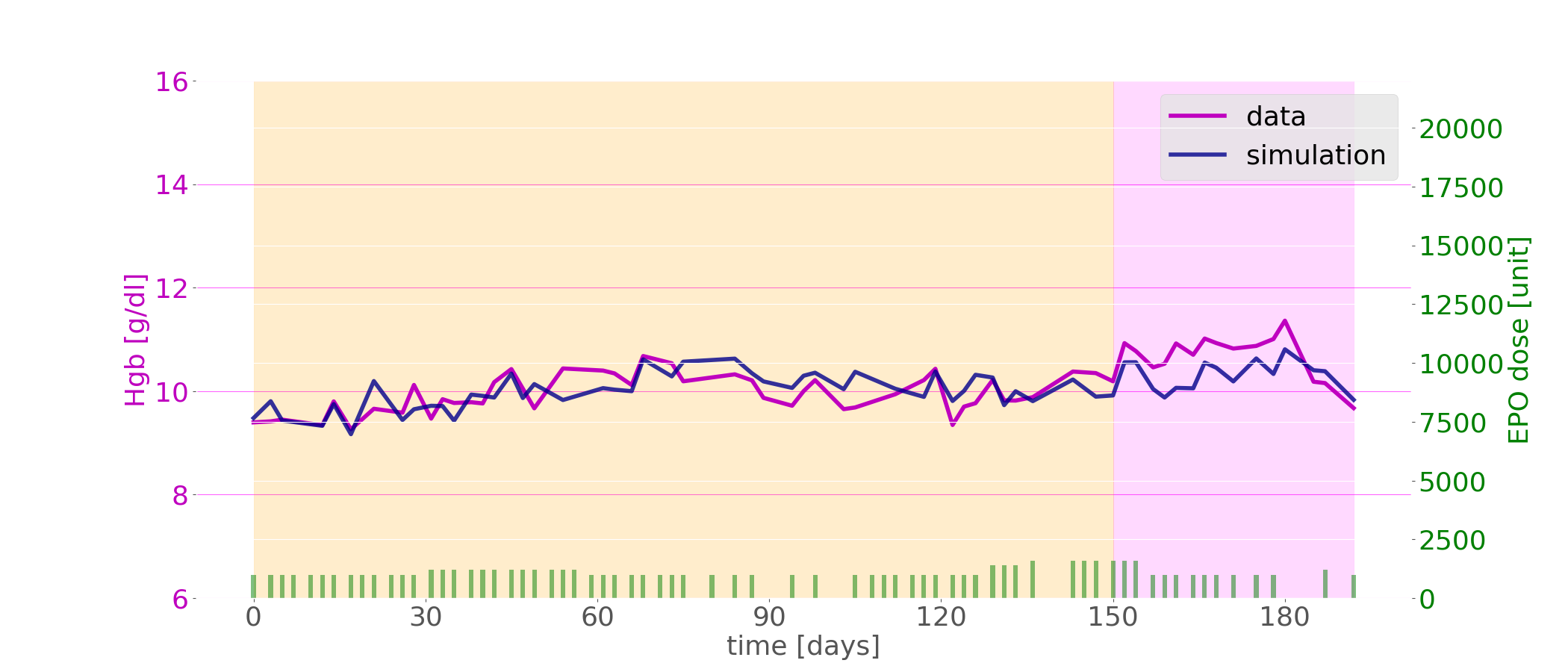

Supplement: S1 Figs — Pre-dialysis Hgb measurements (magenta) and model output (blue) during the model adaptation period (yellow area) and prediction period (purple area). Green bars represent the administered ESA doses. (ZIP) [file pone.0195918.s001.zip › patient_100027.png]

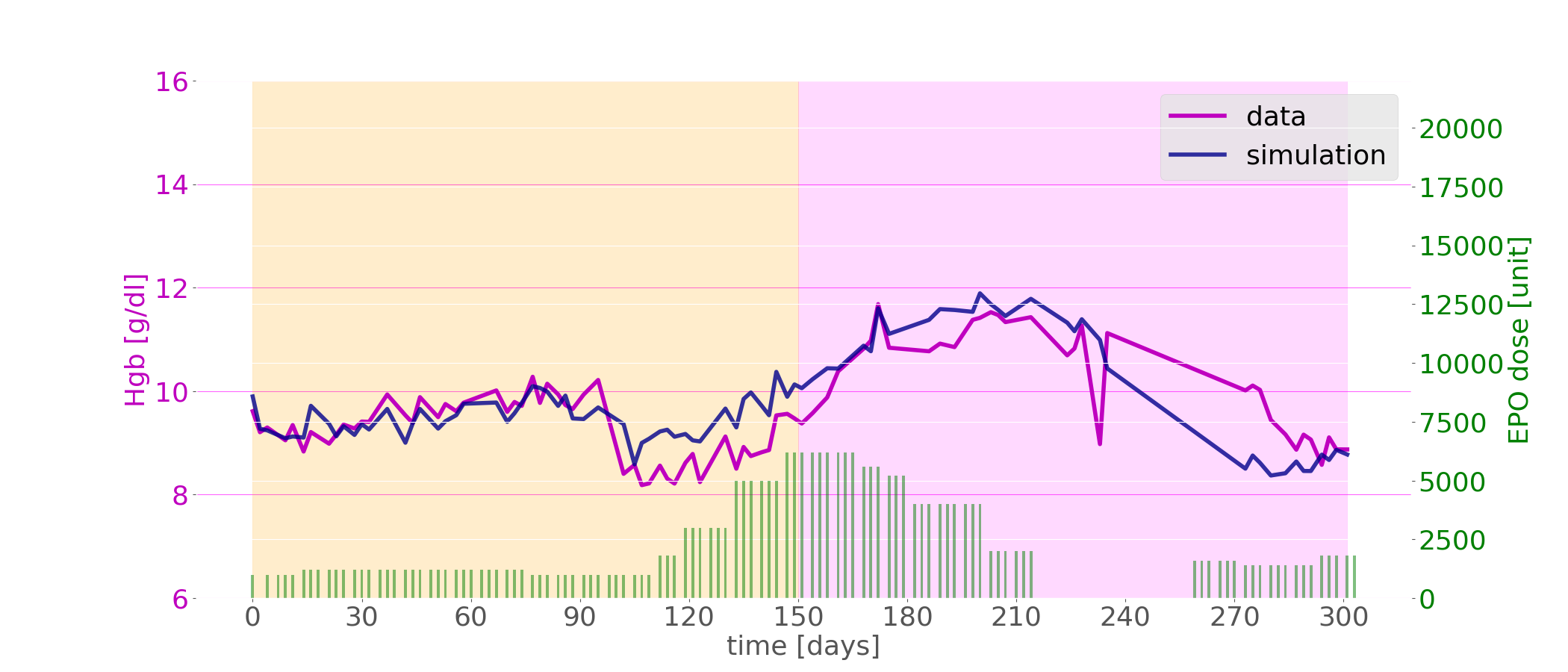

Supplement: S1 Figs — Pre-dialysis Hgb measurements (magenta) and model output (blue) during the model adaptation period (yellow area) and prediction period (purple area). Green bars represent the administered ESA doses. (ZIP) [file pone.0195918.s001.zip › patient_100028.png]

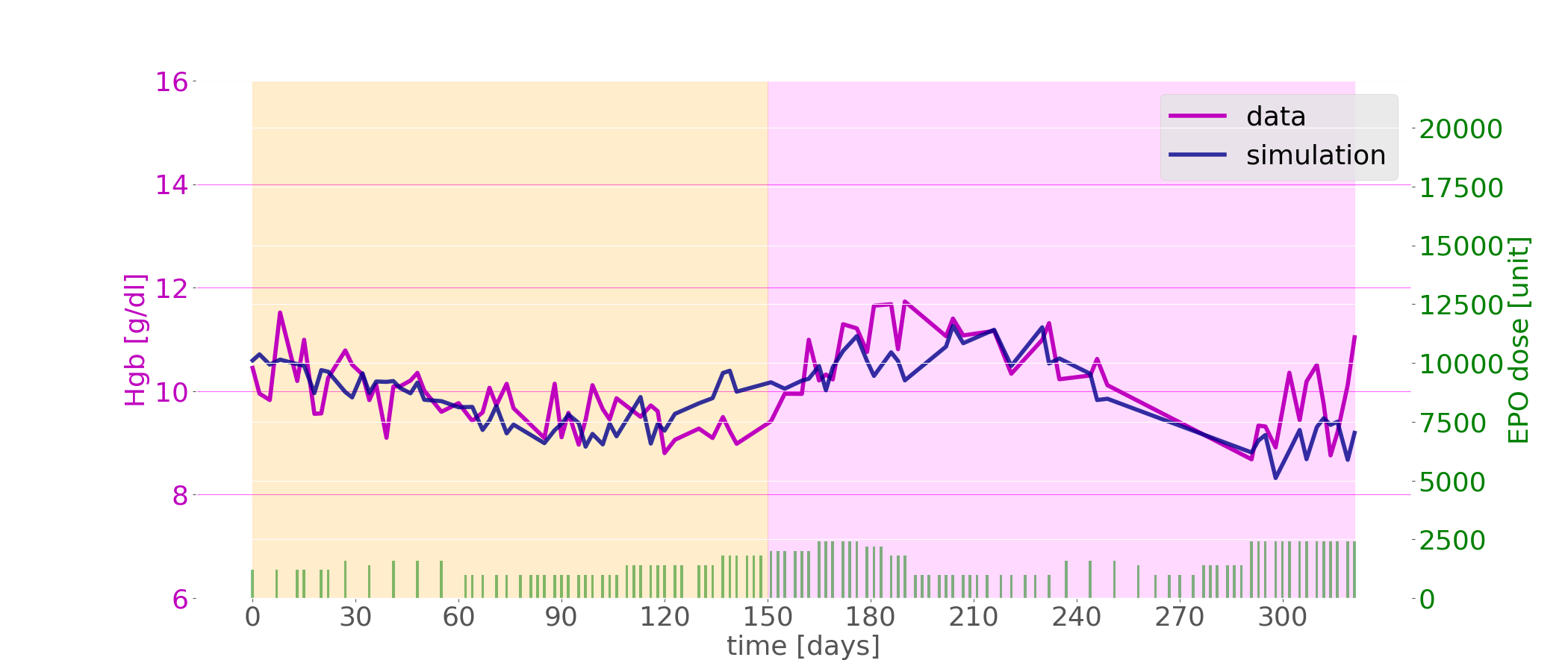

Supplement: S1 Figs — Pre-dialysis Hgb measurements (magenta) and model output (blue) during the model adaptation period (yellow area) and prediction period (purple area). Green bars represent the administered ESA doses. (ZIP) [file pone.0195918.s001.zip › patient_100029.png]

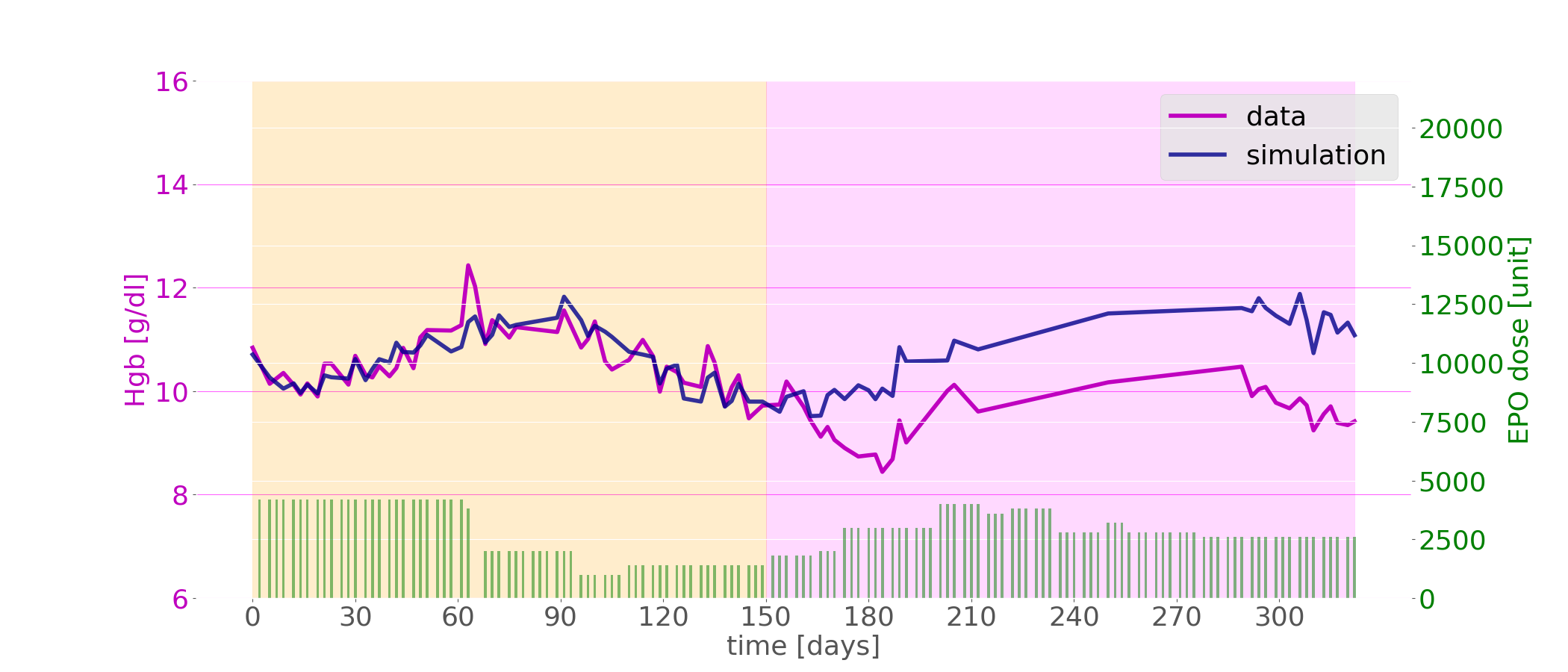

Supplement: S1 Figs — Pre-dialysis Hgb measurements (magenta) and model output (blue) during the model adaptation period (yellow area) and prediction period (purple area). Green bars represent the administered ESA doses. (ZIP) [file pone.0195918.s001.zip › patient_100030.png]

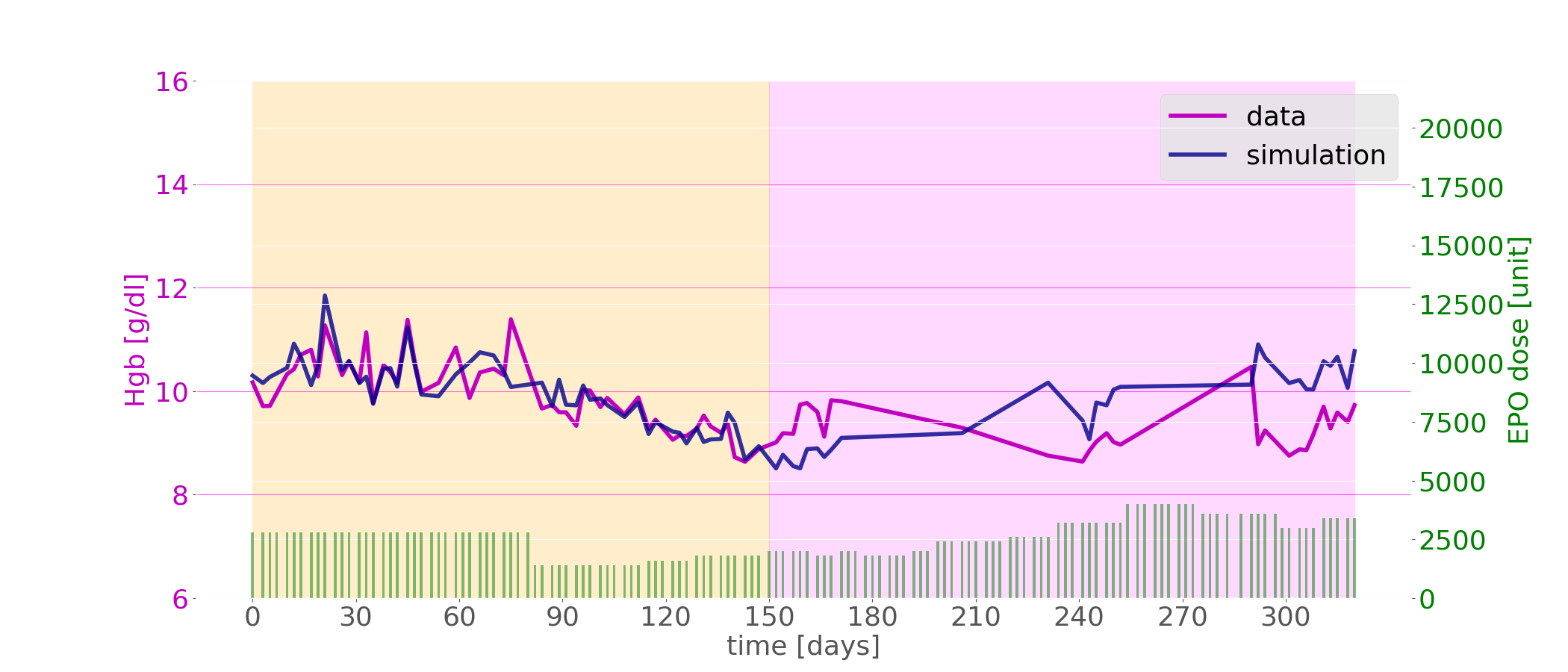

Supplement: S1 Figs — Pre-dialysis Hgb measurements (magenta) and model output (blue) during the model adaptation period (yellow area) and prediction period (purple area). Green bars represent the administered ESA doses. (ZIP) [file pone.0195918.s001.zip › patient_100031.png]

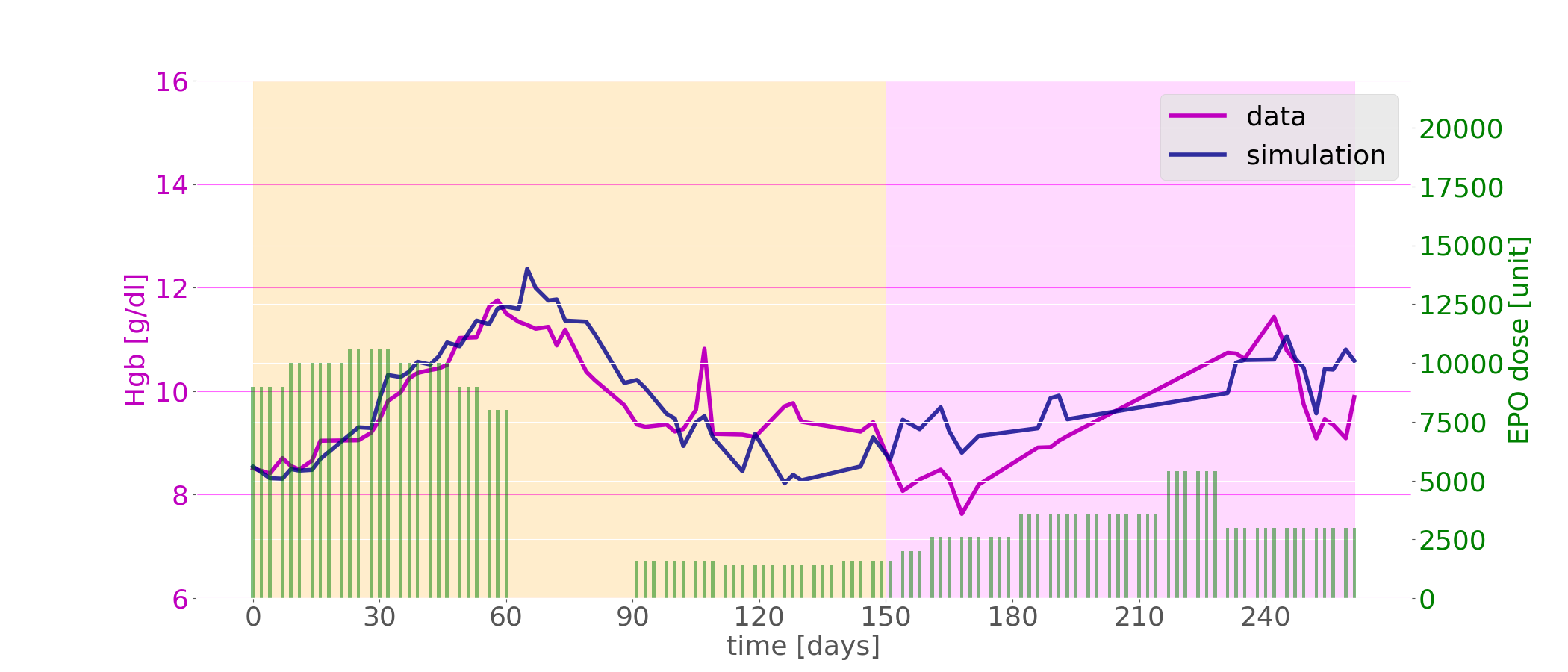

Supplement: S1 Figs — Pre-dialysis Hgb measurements (magenta) and model output (blue) during the model adaptation period (yellow area) and prediction period (purple area). Green bars represent the administered ESA doses. (ZIP) [file pone.0195918.s001.zip › patient_100032.png]

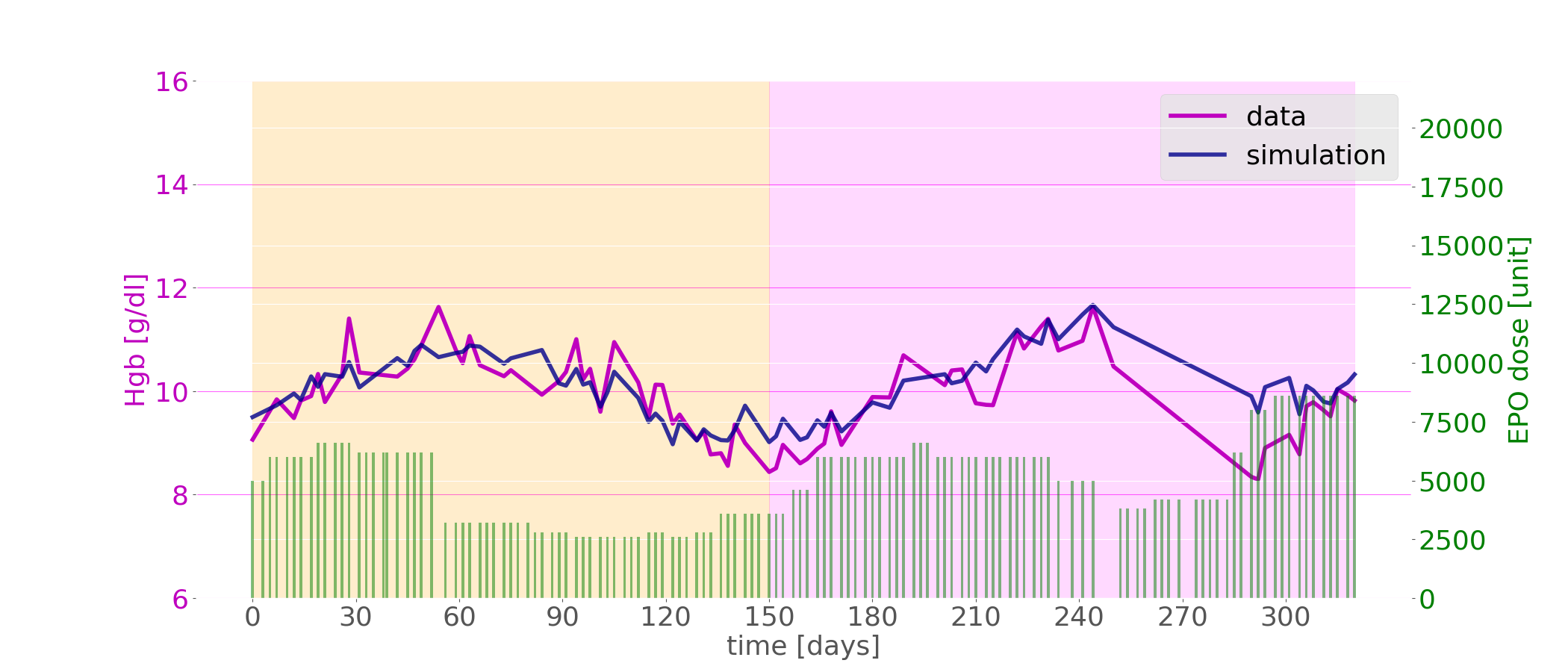

Supplement: S1 Figs — Pre-dialysis Hgb measurements (magenta) and model output (blue) during the model adaptation period (yellow area) and prediction period (purple area). Green bars represent the administered ESA doses. (ZIP) [file pone.0195918.s001.zip › patient_100033.png]

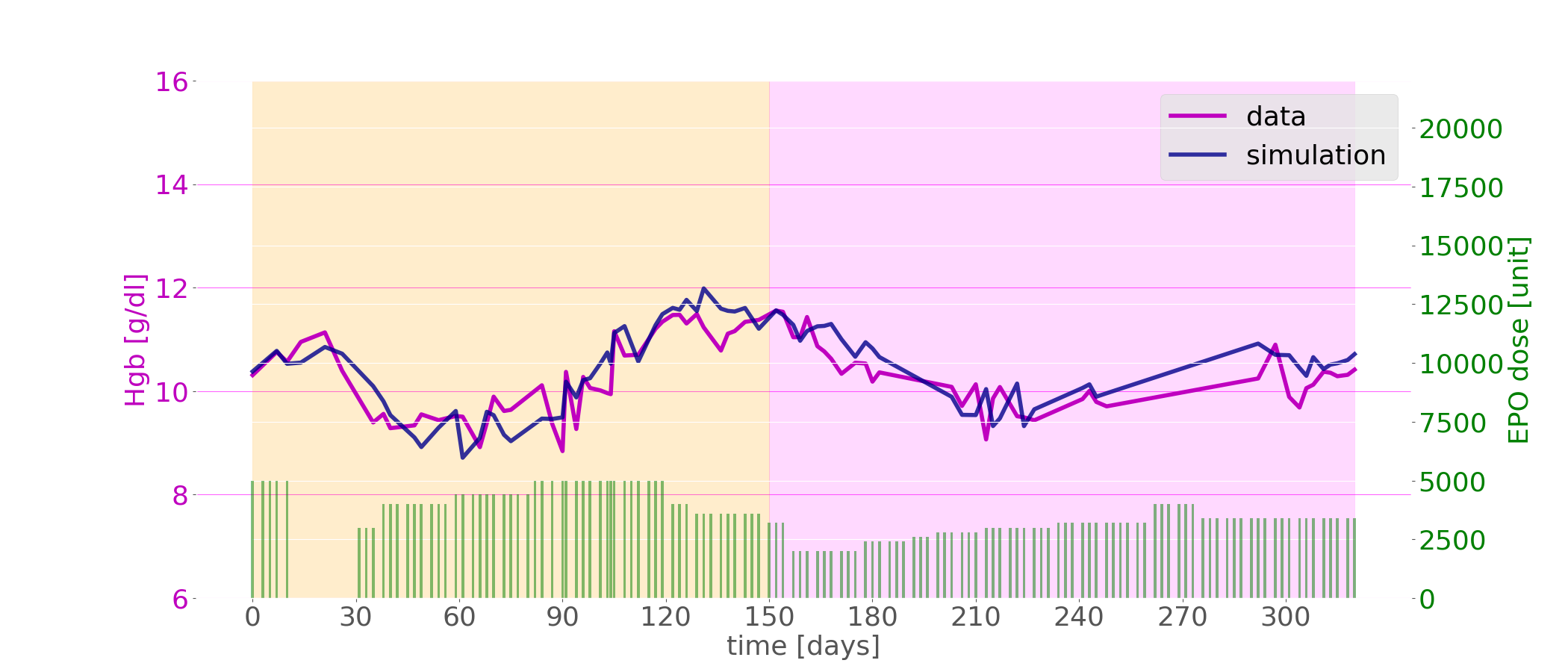

Supplement: S1 Figs — Pre-dialysis Hgb measurements (magenta) and model output (blue) during the model adaptation period (yellow area) and prediction period (purple area). Green bars represent the administered ESA doses. (ZIP) [file pone.0195918.s001.zip › patient_100034.png]

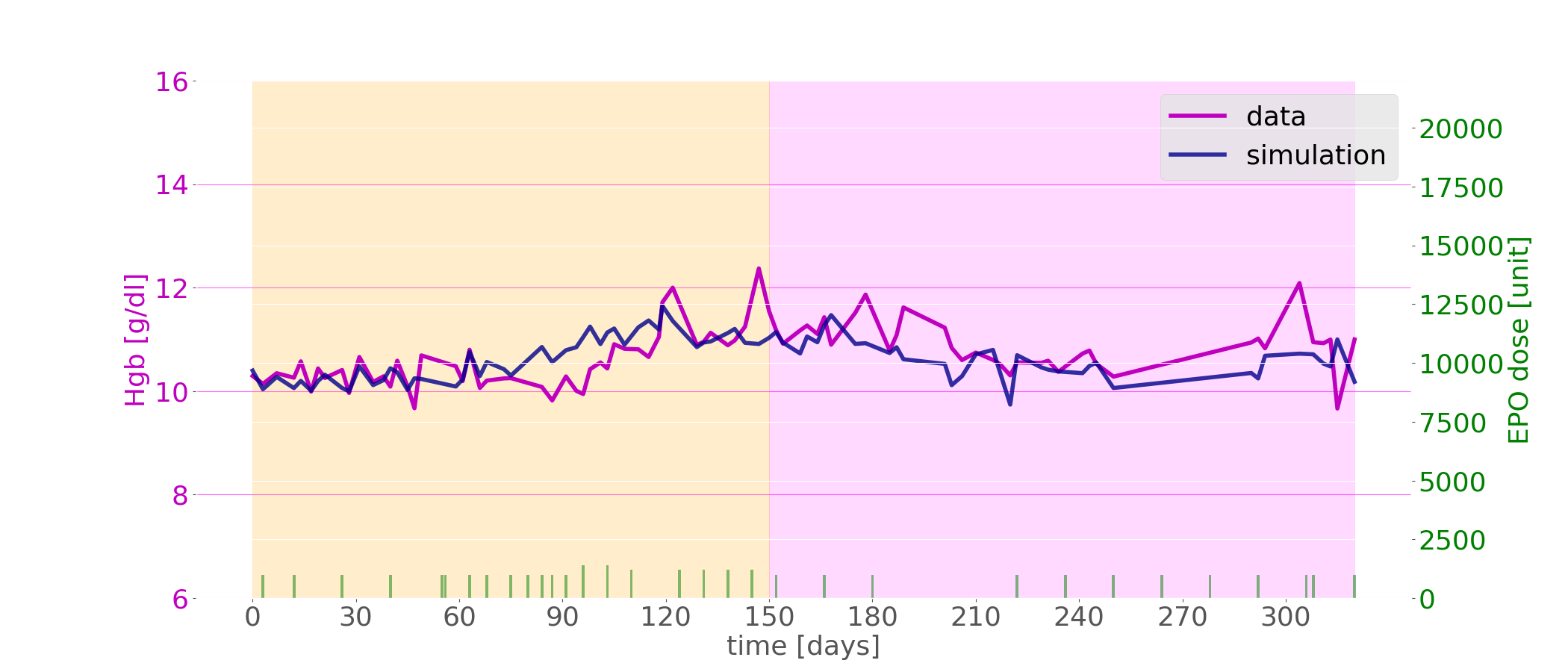

Supplement: S1 Figs — Pre-dialysis Hgb measurements (magenta) and model output (blue) during the model adaptation period (yellow area) and prediction period (purple area). Green bars represent the administered ESA doses. (ZIP) [file pone.0195918.s001.zip › patient_100035.png]

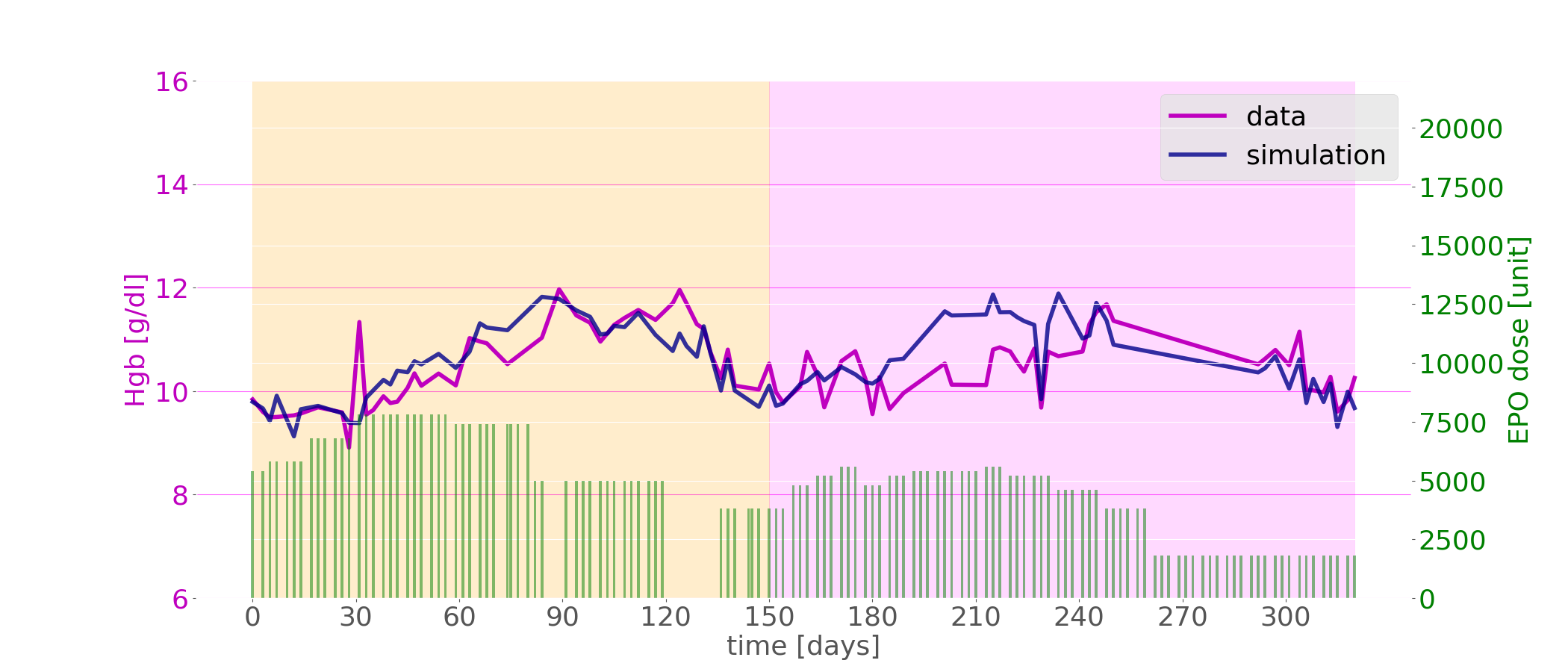

Supplement: S1 Figs — Pre-dialysis Hgb measurements (magenta) and model output (blue) during the model adaptation period (yellow area) and prediction period (purple area). Green bars represent the administered ESA doses. (ZIP) [file pone.0195918.s001.zip › patient_100036.png]

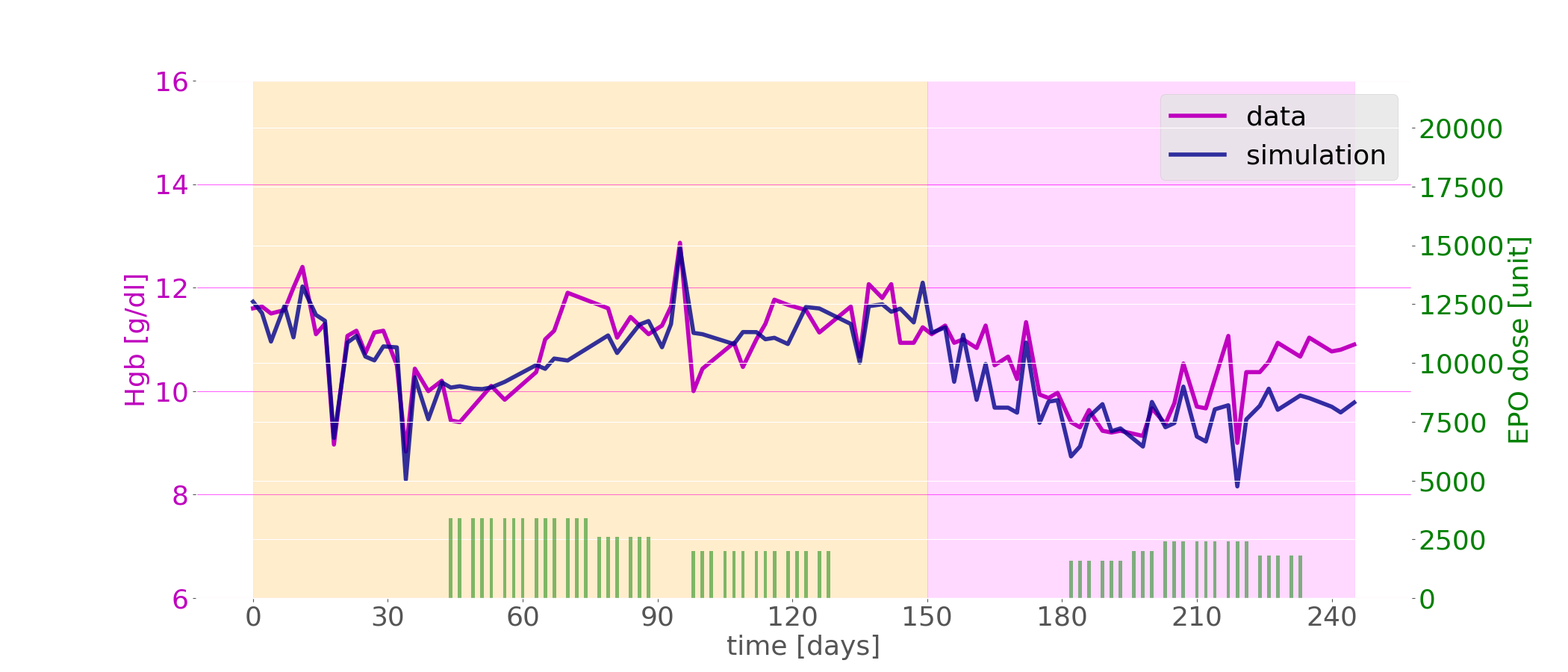

Supplement: S1 Figs — Pre-dialysis Hgb measurements (magenta) and model output (blue) during the model adaptation period (yellow area) and prediction period (purple area). Green bars represent the administered ESA doses. (ZIP) [file pone.0195918.s001.zip › patient_100037.png]

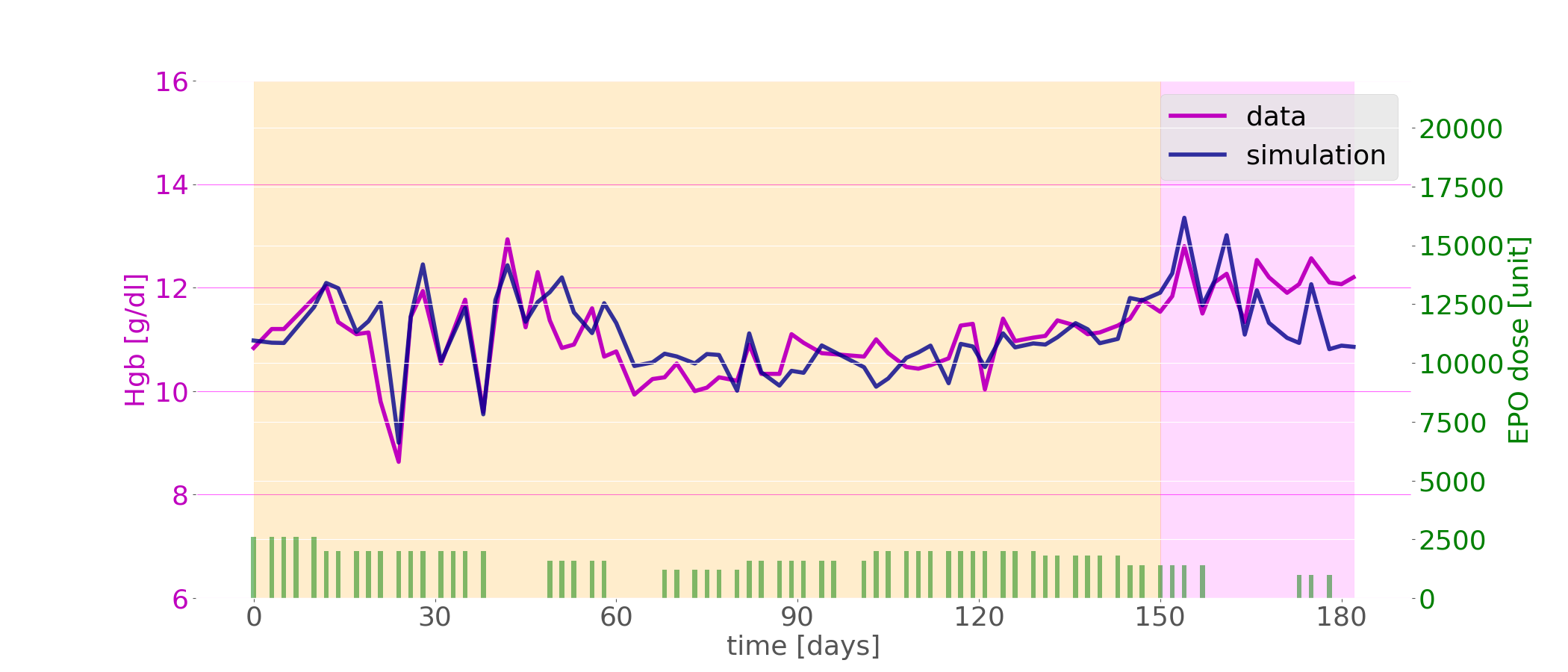

Supplement: S1 Figs — Pre-dialysis Hgb measurements (magenta) and model output (blue) during the model adaptation period (yellow area) and prediction period (purple area). Green bars represent the administered ESA doses. (ZIP) [file pone.0195918.s001.zip › patient_100038.png]

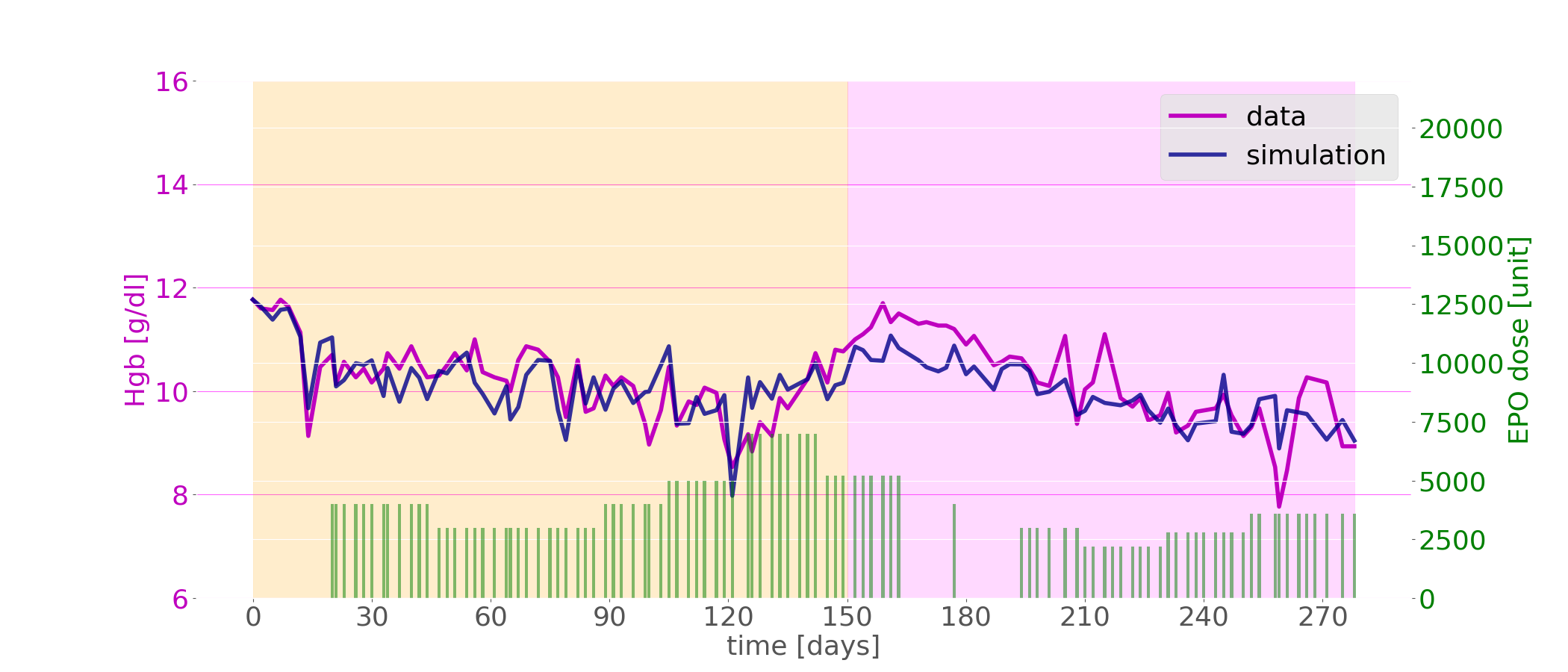

Supplement: S1 Figs — Pre-dialysis Hgb measurements (magenta) and model output (blue) during the model adaptation period (yellow area) and prediction period (purple area). Green bars represent the administered ESA doses. (ZIP) [file pone.0195918.s001.zip › patient_100039.png]

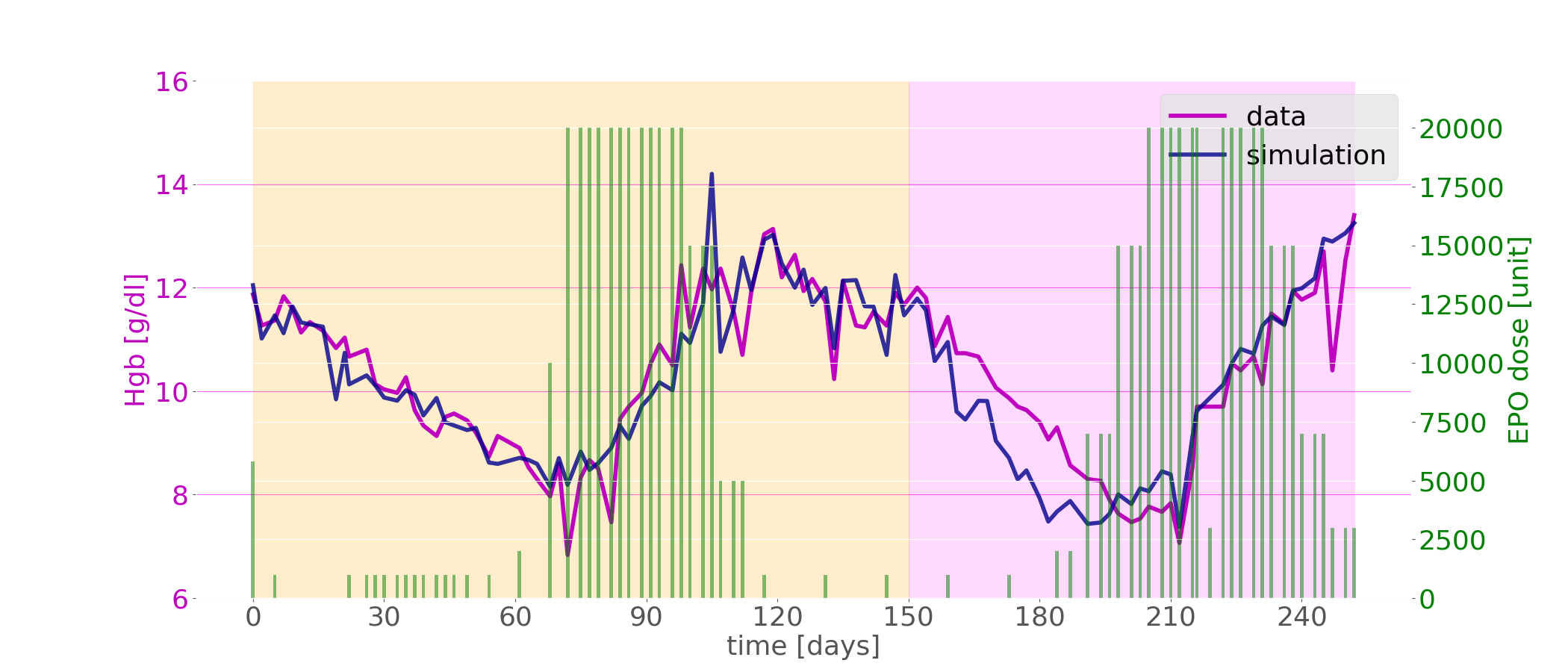

Supplement: S1 Figs — Pre-dialysis Hgb measurements (magenta) and model output (blue) during the model adaptation period (yellow area) and prediction period (purple area). Green bars represent the administered ESA doses. (ZIP) [file pone.0195918.s001.zip › patient_100040.png]

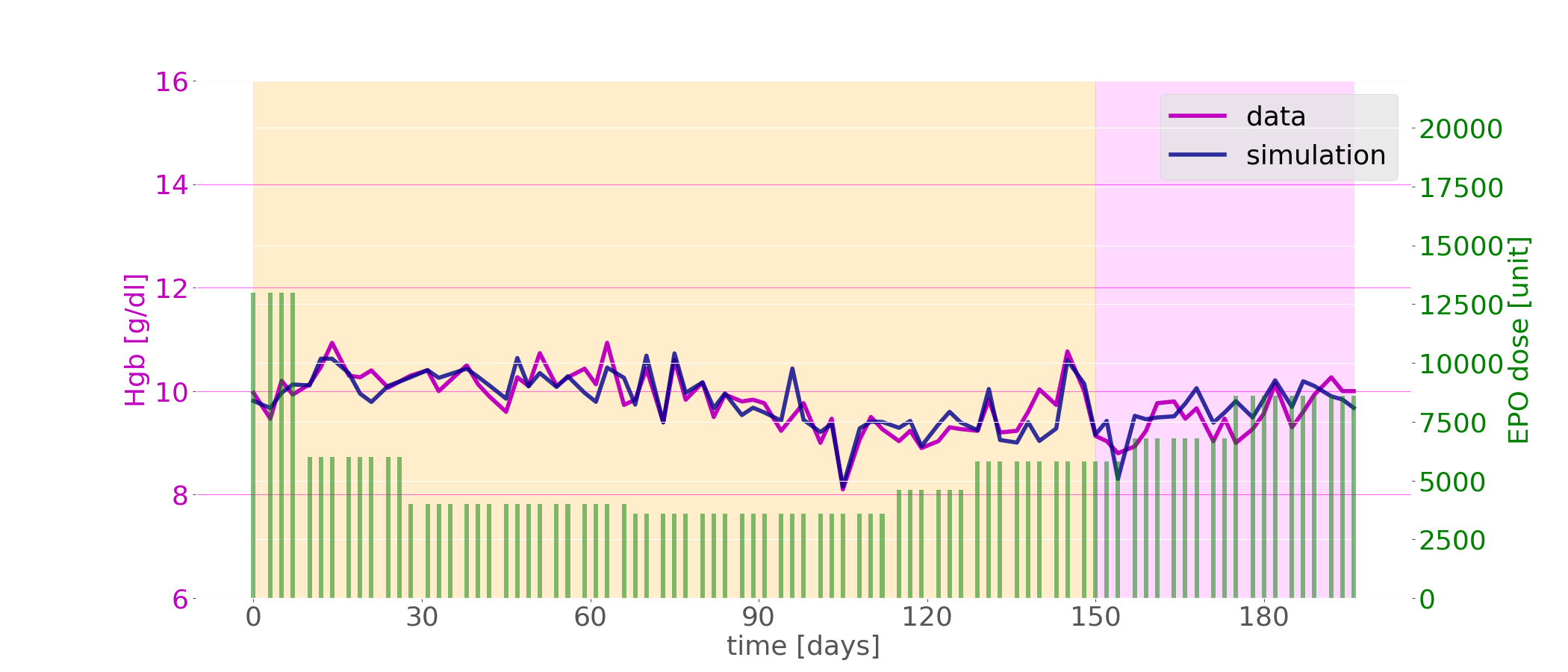

Supplement: S1 Figs — Pre-dialysis Hgb measurements (magenta) and model output (blue) during the model adaptation period (yellow area) and prediction period (purple area). Green bars represent the administered ESA doses. (ZIP) [file pone.0195918.s001.zip › patient_100041.png]

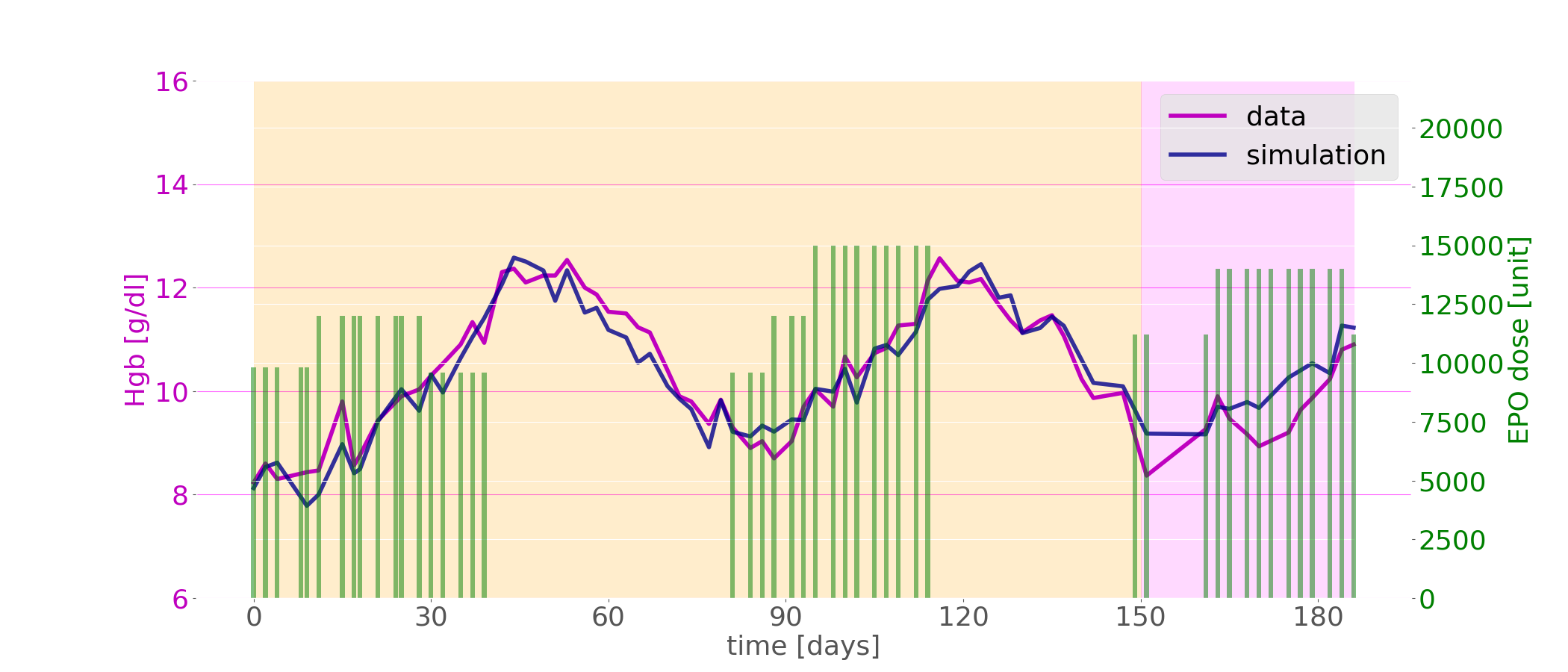

Supplement: S1 Figs — Pre-dialysis Hgb measurements (magenta) and model output (blue) during the model adaptation period (yellow area) and prediction period (purple area). Green bars represent the administered ESA doses. (ZIP) [file pone.0195918.s001.zip › patient_100042.png]

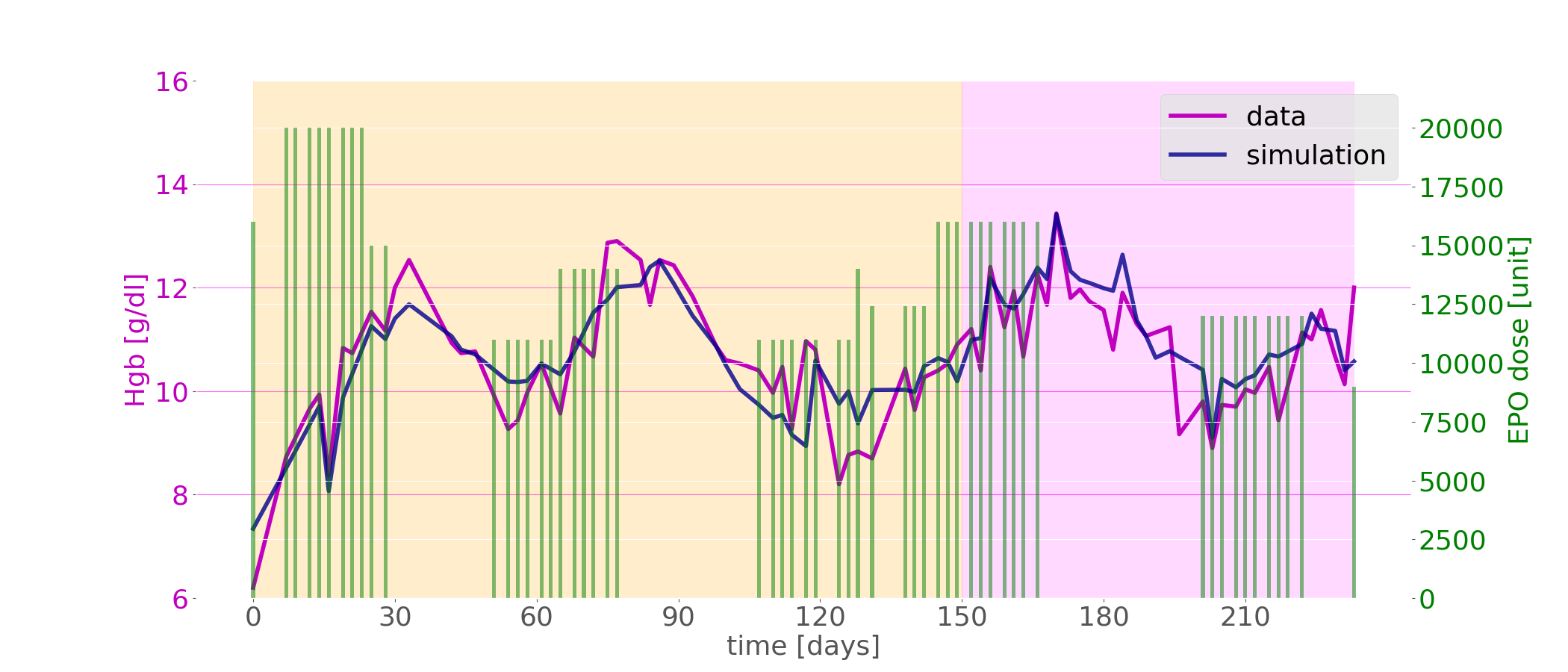

Supplement: S1 Figs — Pre-dialysis Hgb measurements (magenta) and model output (blue) during the model adaptation period (yellow area) and prediction period (purple area). Green bars represent the administered ESA doses. (ZIP) [file pone.0195918.s001.zip › patient_100043.png]

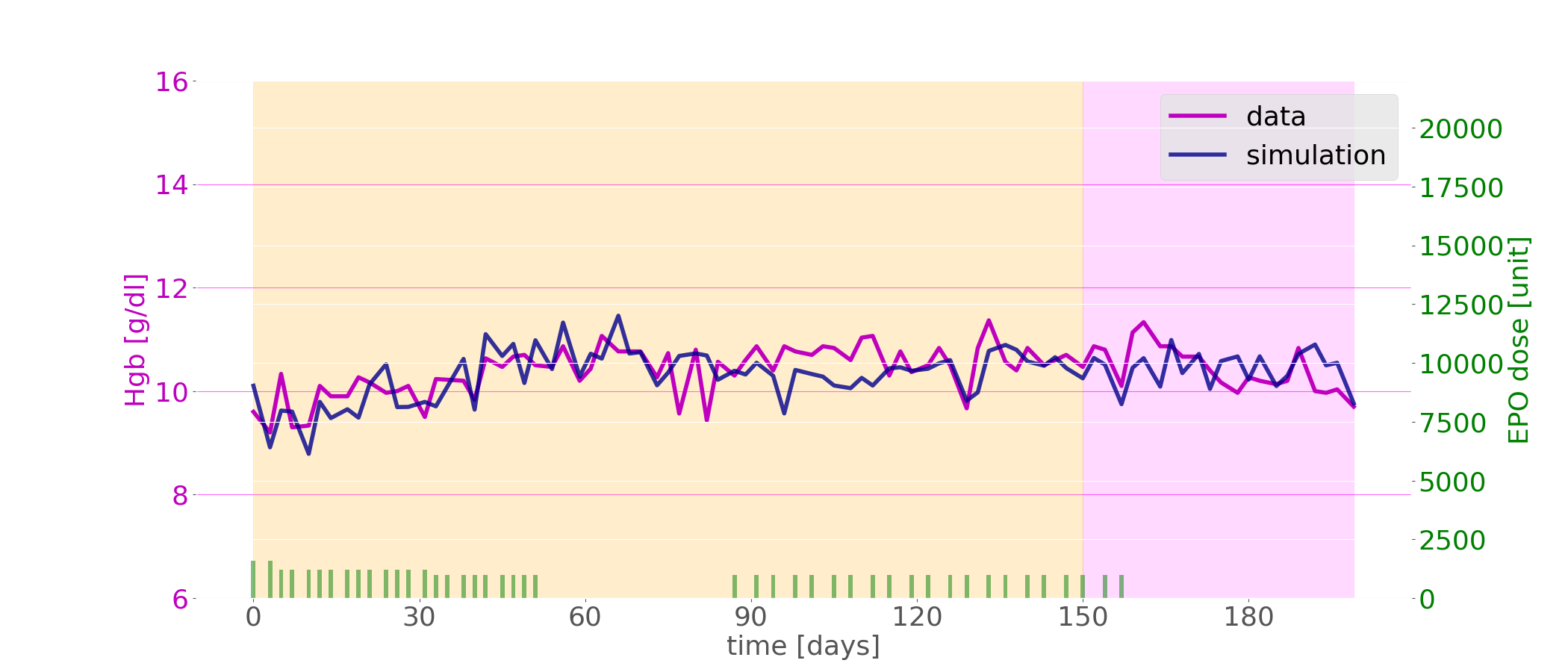

Supplement: S1 Figs — Pre-dialysis Hgb measurements (magenta) and model output (blue) during the model adaptation period (yellow area) and prediction period (purple area). Green bars represent the administered ESA doses. (ZIP) [file pone.0195918.s001.zip › patient_100044.png]

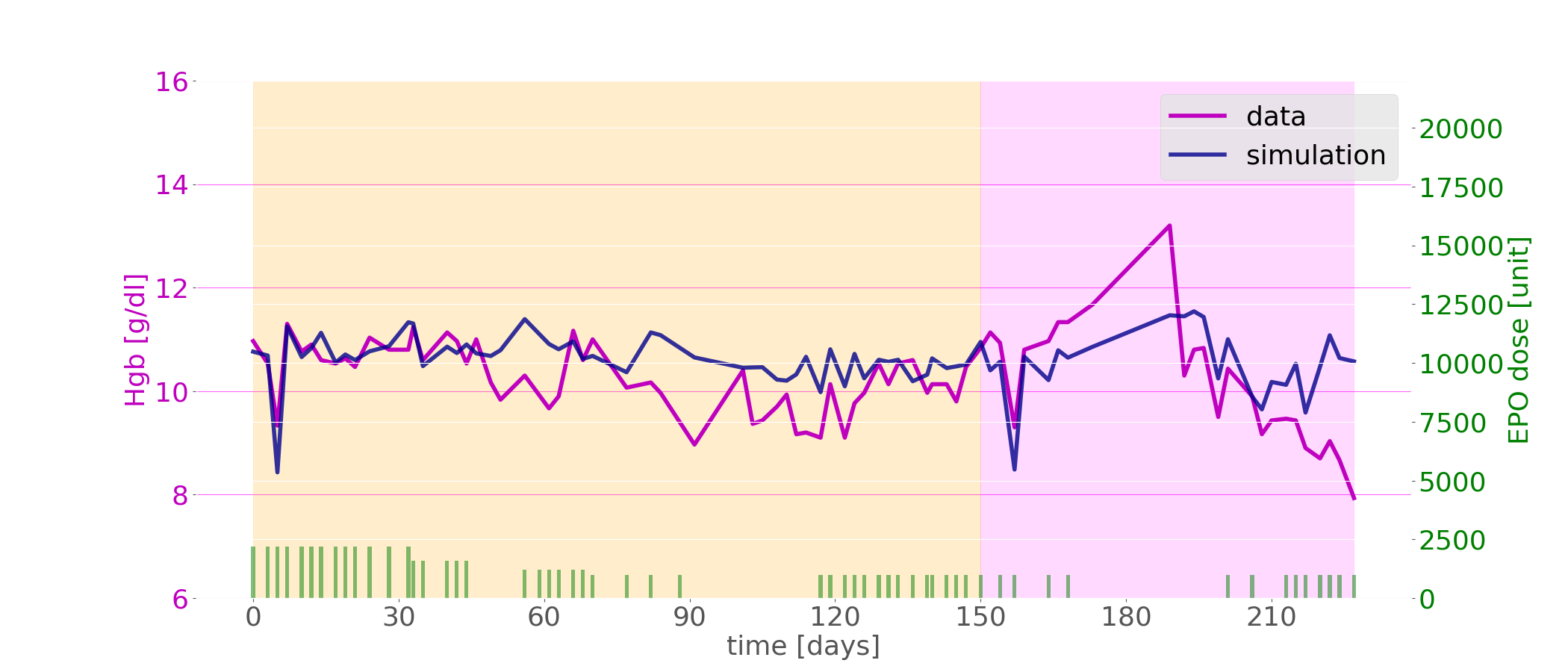

Supplement: S1 Figs — Pre-dialysis Hgb measurements (magenta) and model output (blue) during the model adaptation period (yellow area) and prediction period (purple area). Green bars represent the administered ESA doses. (ZIP) [file pone.0195918.s001.zip › patient_100045.png]

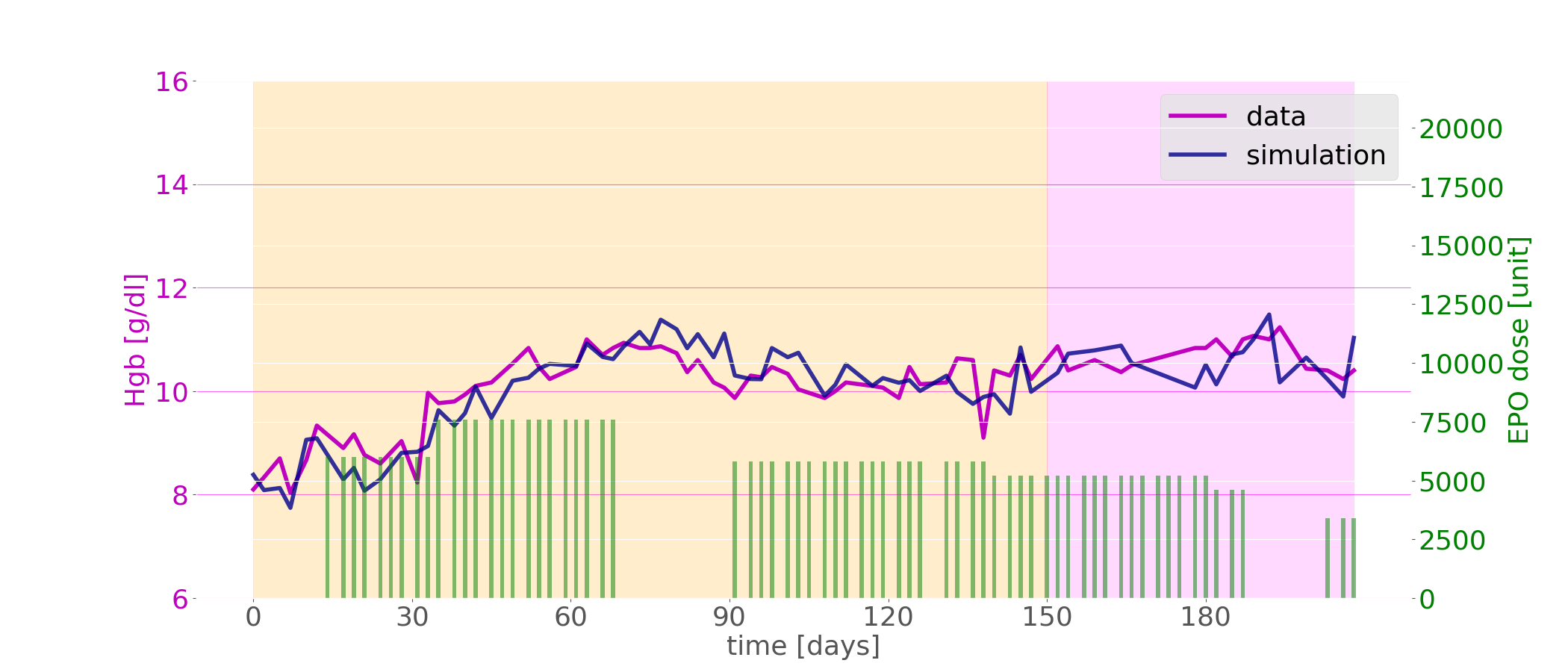

Supplement: S1 Figs — Pre-dialysis Hgb measurements (magenta) and model output (blue) during the model adaptation period (yellow area) and prediction period (purple area). Green bars represent the administered ESA doses. (ZIP) [file pone.0195918.s001.zip › patient_100046.png]

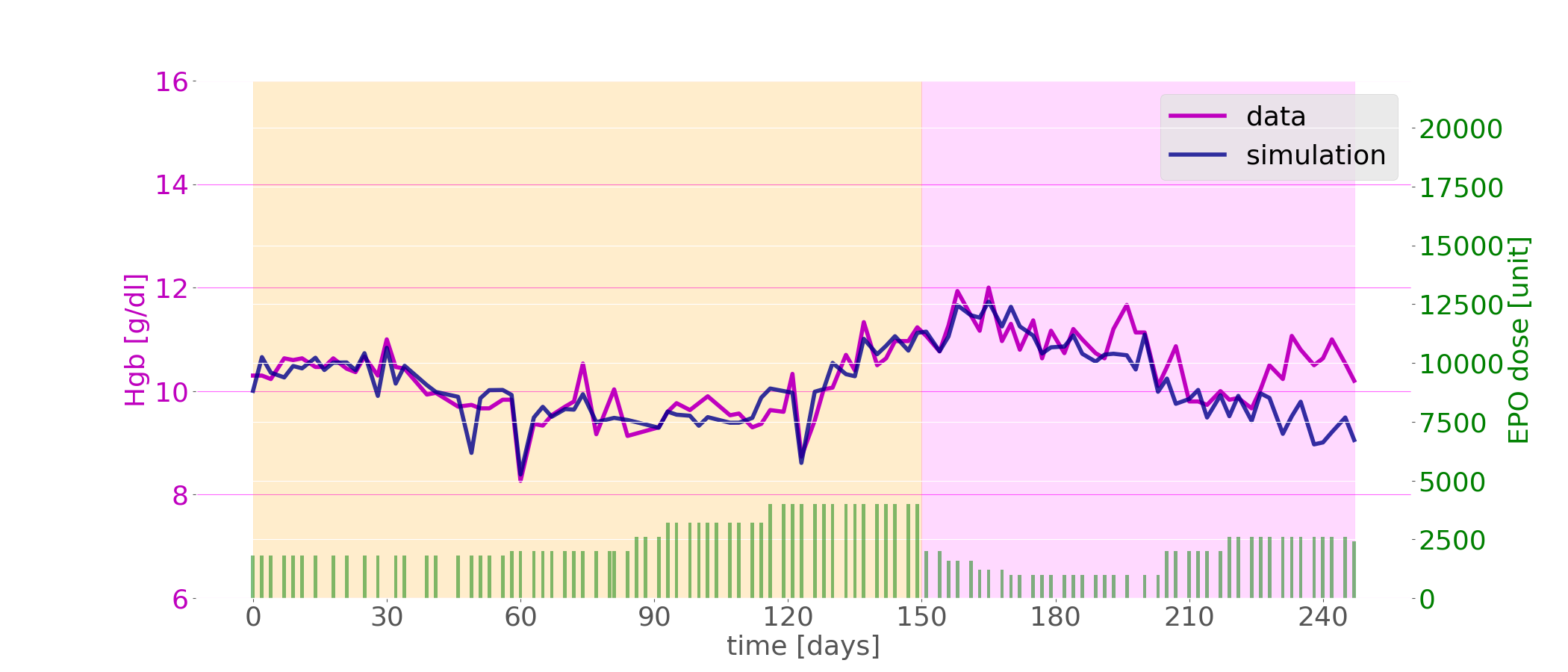

Supplement: S1 Figs — Pre-dialysis Hgb measurements (magenta) and model output (blue) during the model adaptation period (yellow area) and prediction period (purple area). Green bars represent the administered ESA doses. (ZIP) [file pone.0195918.s001.zip › patient_100047.png]

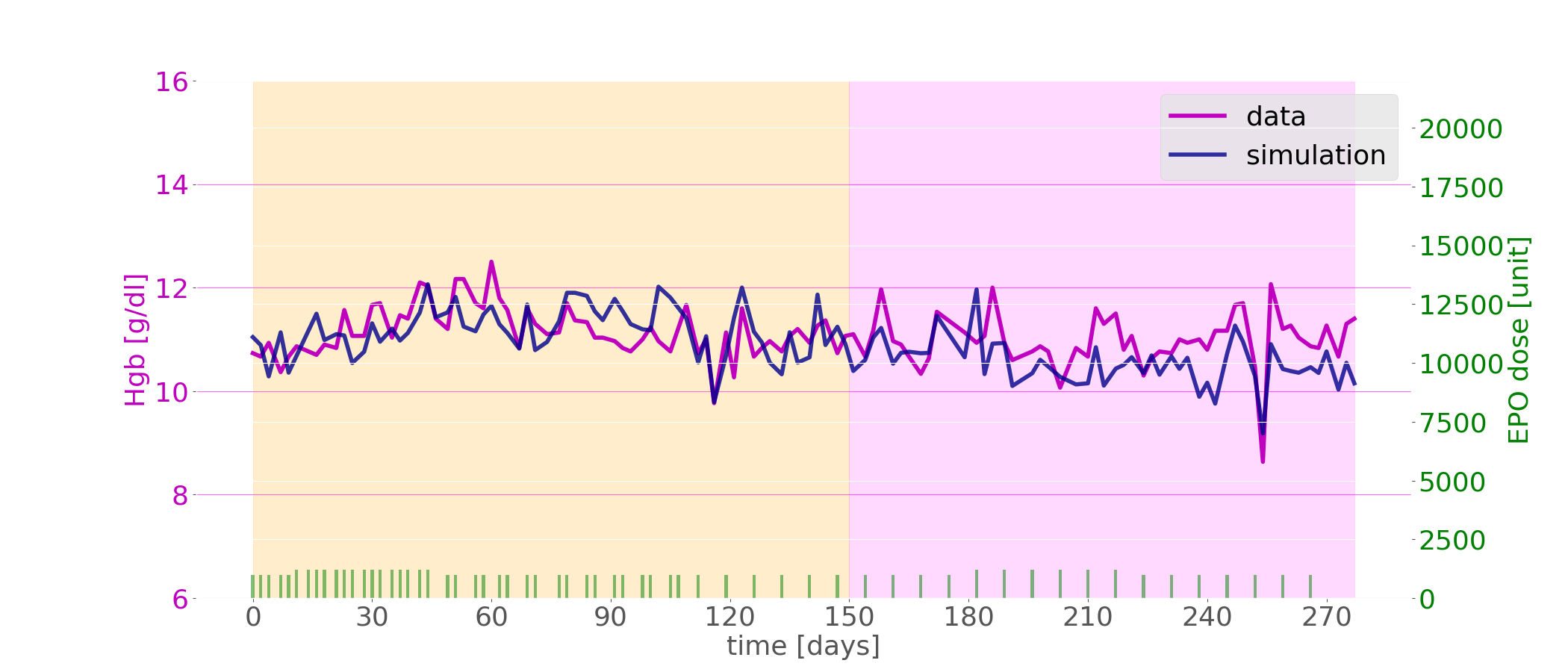

Supplement: S1 Figs — Pre-dialysis Hgb measurements (magenta) and model output (blue) during the model adaptation period (yellow area) and prediction period (purple area). Green bars represent the administered ESA doses. (ZIP) [file pone.0195918.s001.zip › patient_100048.png]

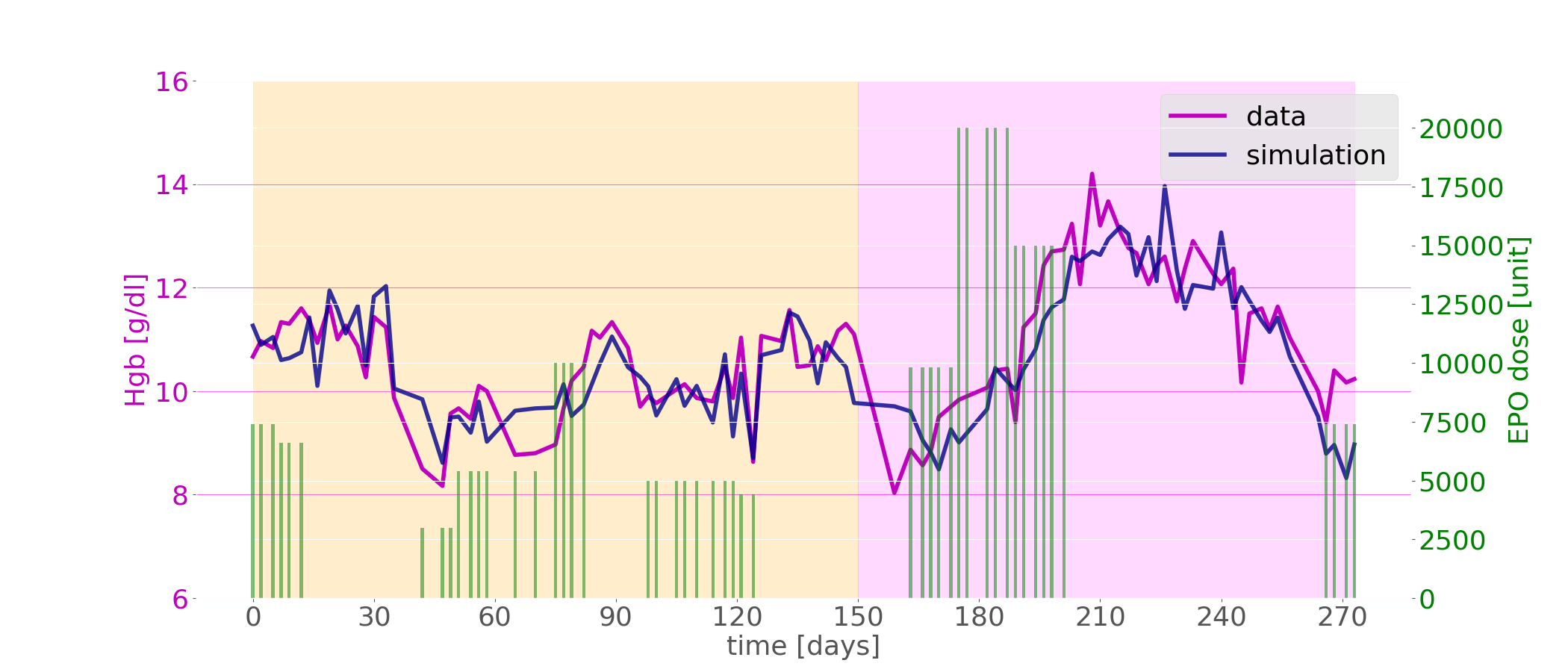

Supplement: S1 Figs — Pre-dialysis Hgb measurements (magenta) and model output (blue) during the model adaptation period (yellow area) and prediction period (purple area). Green bars represent the administered ESA doses. (ZIP) [file pone.0195918.s001.zip › patient_100049.png]

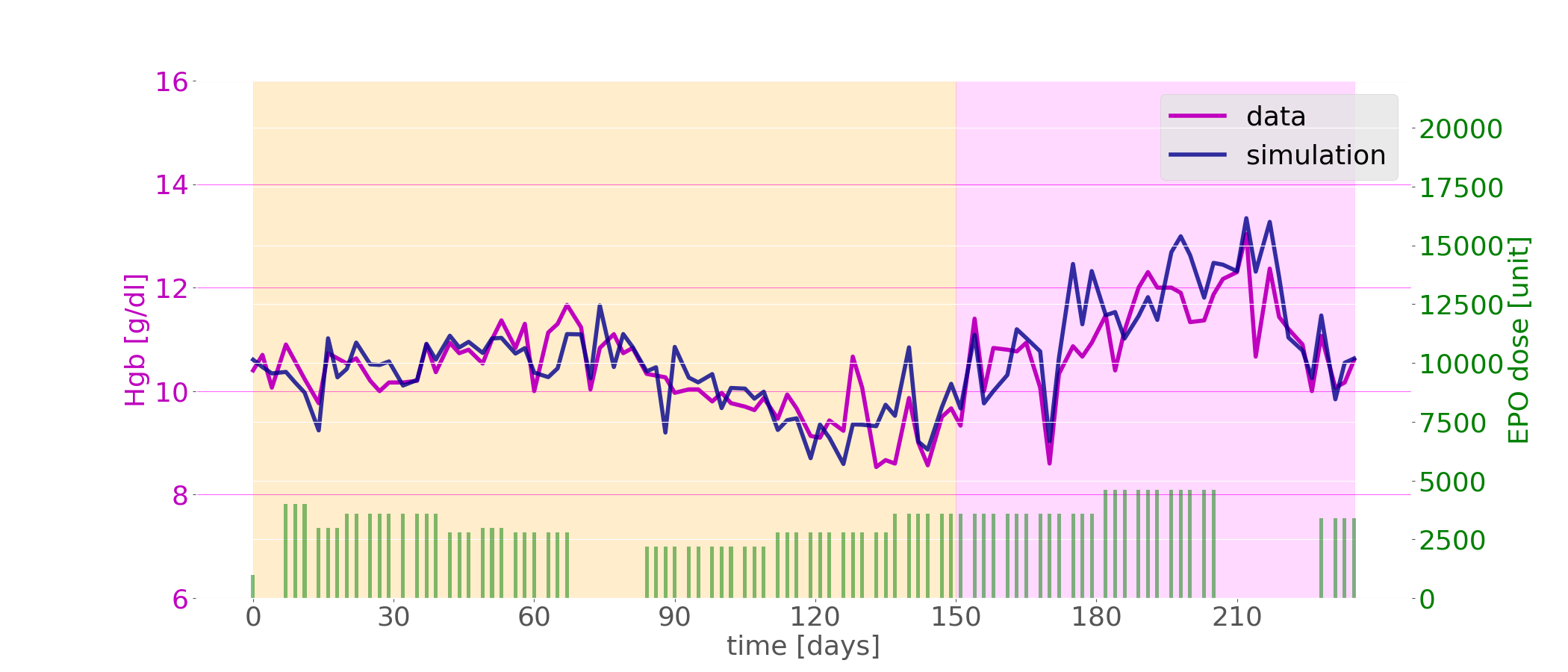

Supplement: S1 Figs — Pre-dialysis Hgb measurements (magenta) and model output (blue) during the model adaptation period (yellow area) and prediction period (purple area). Green bars represent the administered ESA doses. (ZIP) [file pone.0195918.s001.zip › patient_100050.png]

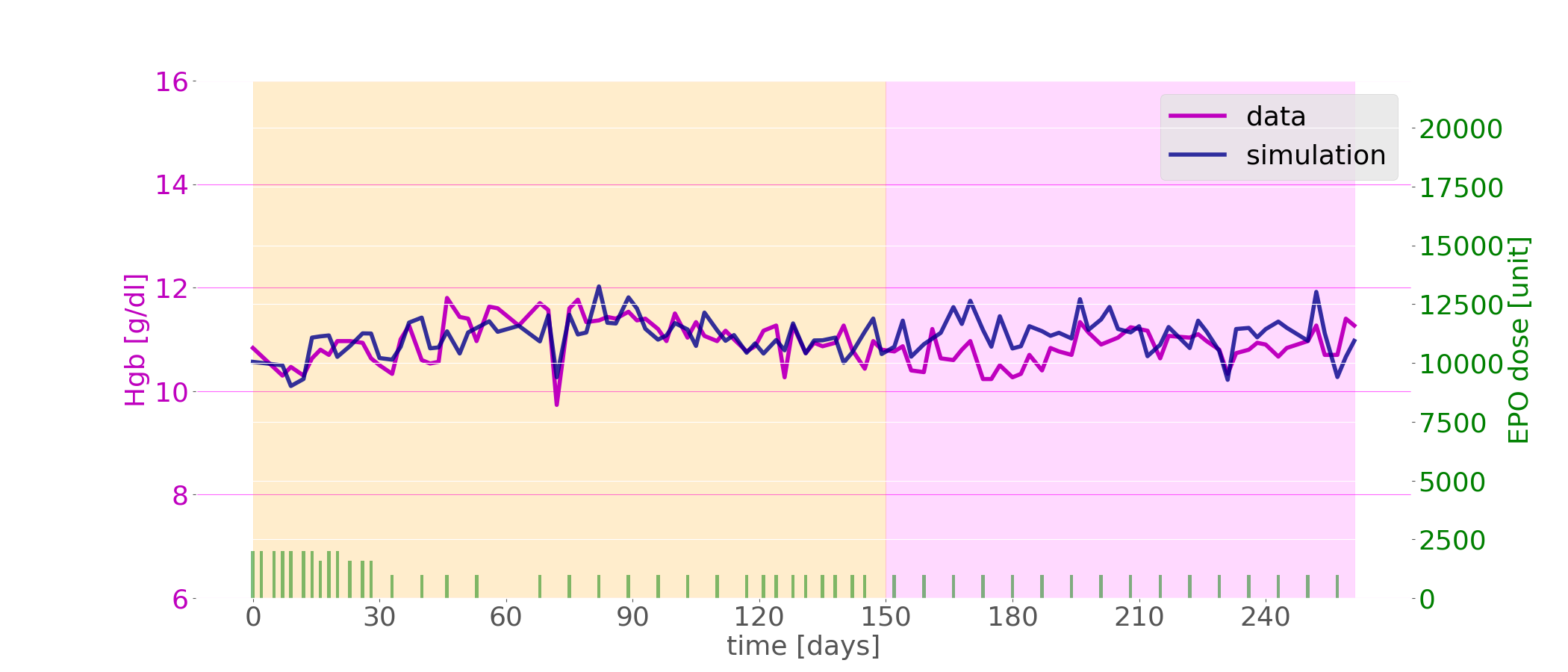

Supplement: S1 Figs — Pre-dialysis Hgb measurements (magenta) and model output (blue) during the model adaptation period (yellow area) and prediction period (purple area). Green bars represent the administered ESA doses. (ZIP) [file pone.0195918.s001.zip › patient_100051.png]

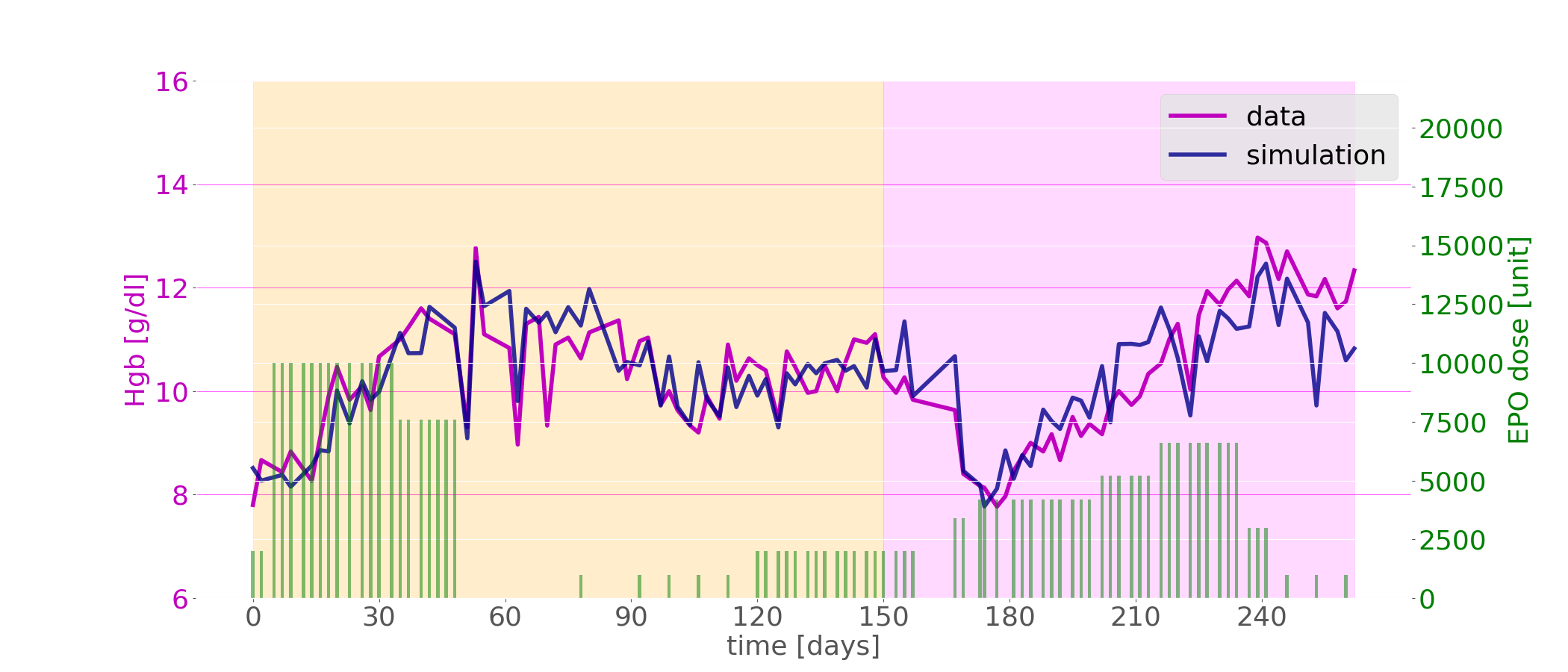

Supplement: S1 Figs — Pre-dialysis Hgb measurements (magenta) and model output (blue) during the model adaptation period (yellow area) and prediction period (purple area). Green bars represent the administered ESA doses. (ZIP) [file pone.0195918.s001.zip › patient_100052.png]

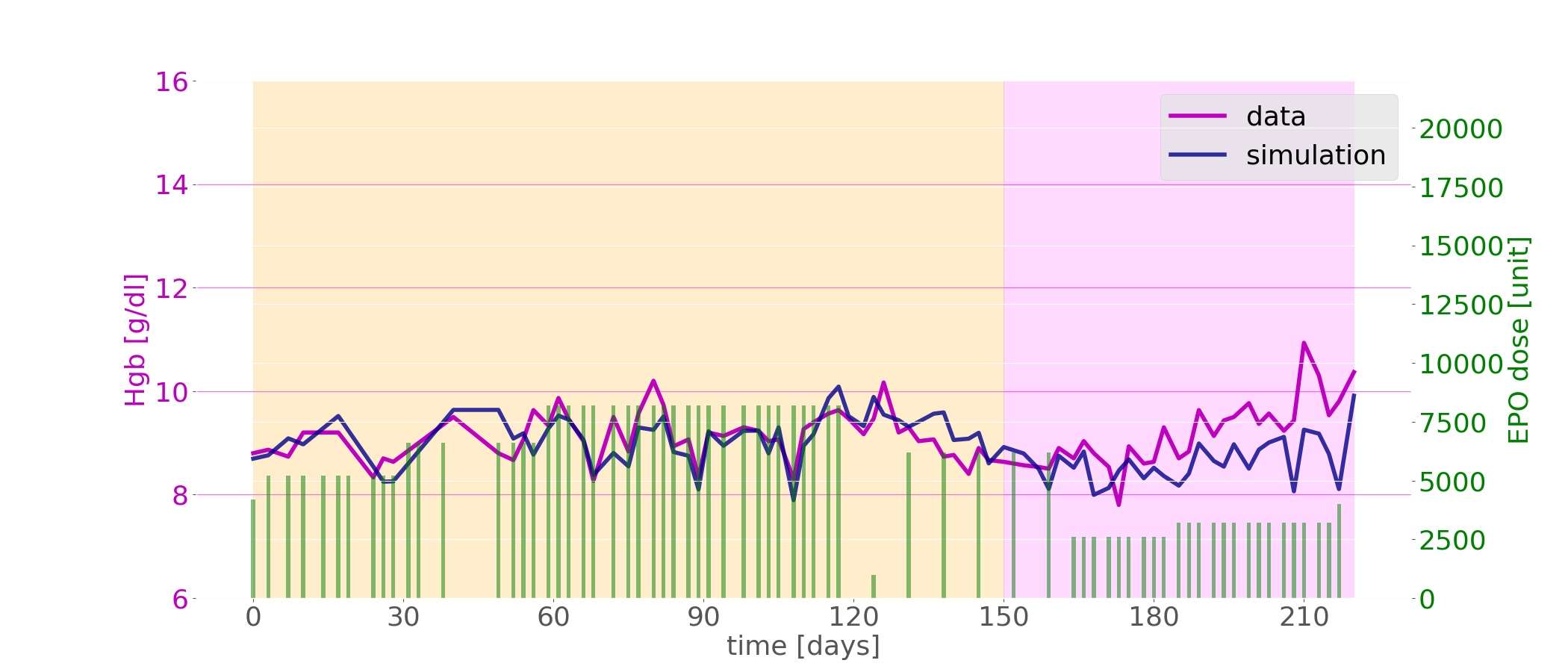

Supplement: S1 Figs — Pre-dialysis Hgb measurements (magenta) and model output (blue) during the model adaptation period (yellow area) and prediction period (purple area). Green bars represent the administered ESA doses. (ZIP) [file pone.0195918.s001.zip › patient_100053.png]

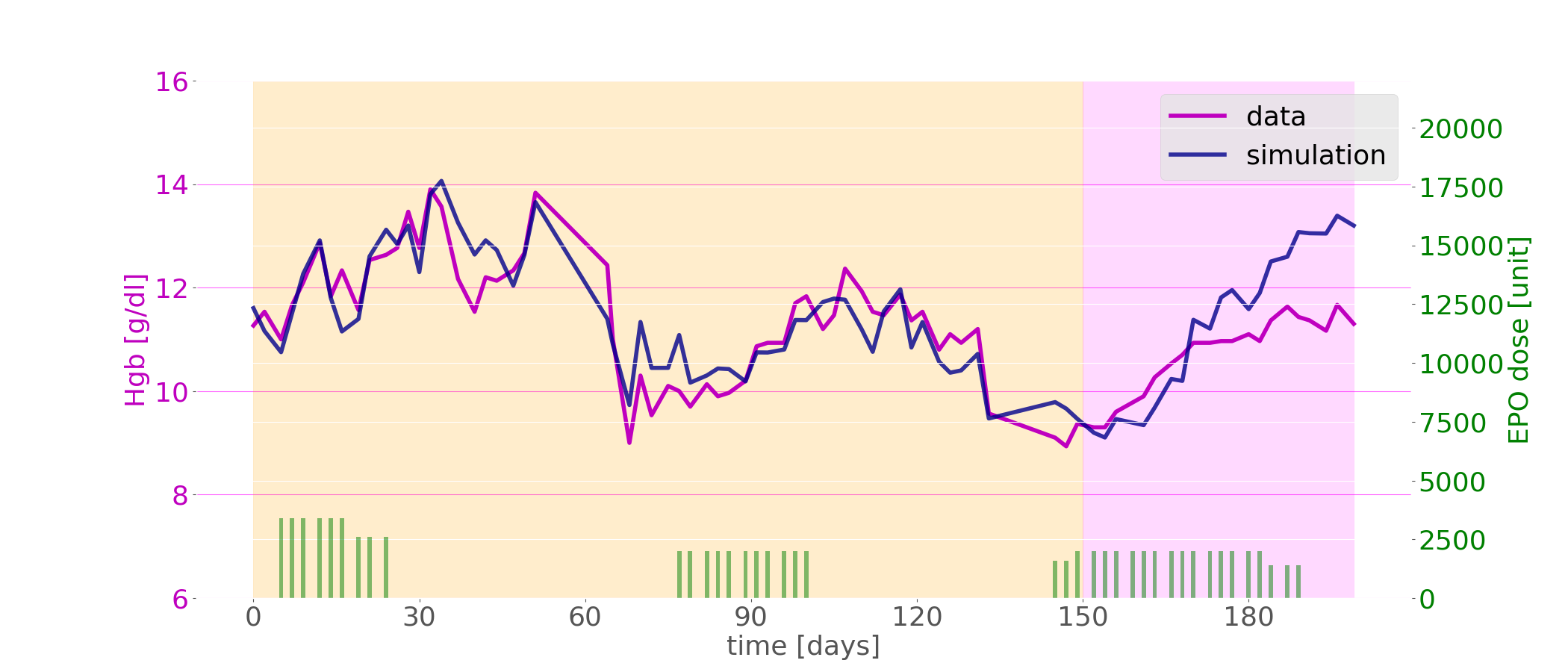

Supplement: S1 Figs — Pre-dialysis Hgb measurements (magenta) and model output (blue) during the model adaptation period (yellow area) and prediction period (purple area). Green bars represent the administered ESA doses. (ZIP) [file pone.0195918.s001.zip › patient_100054.png]

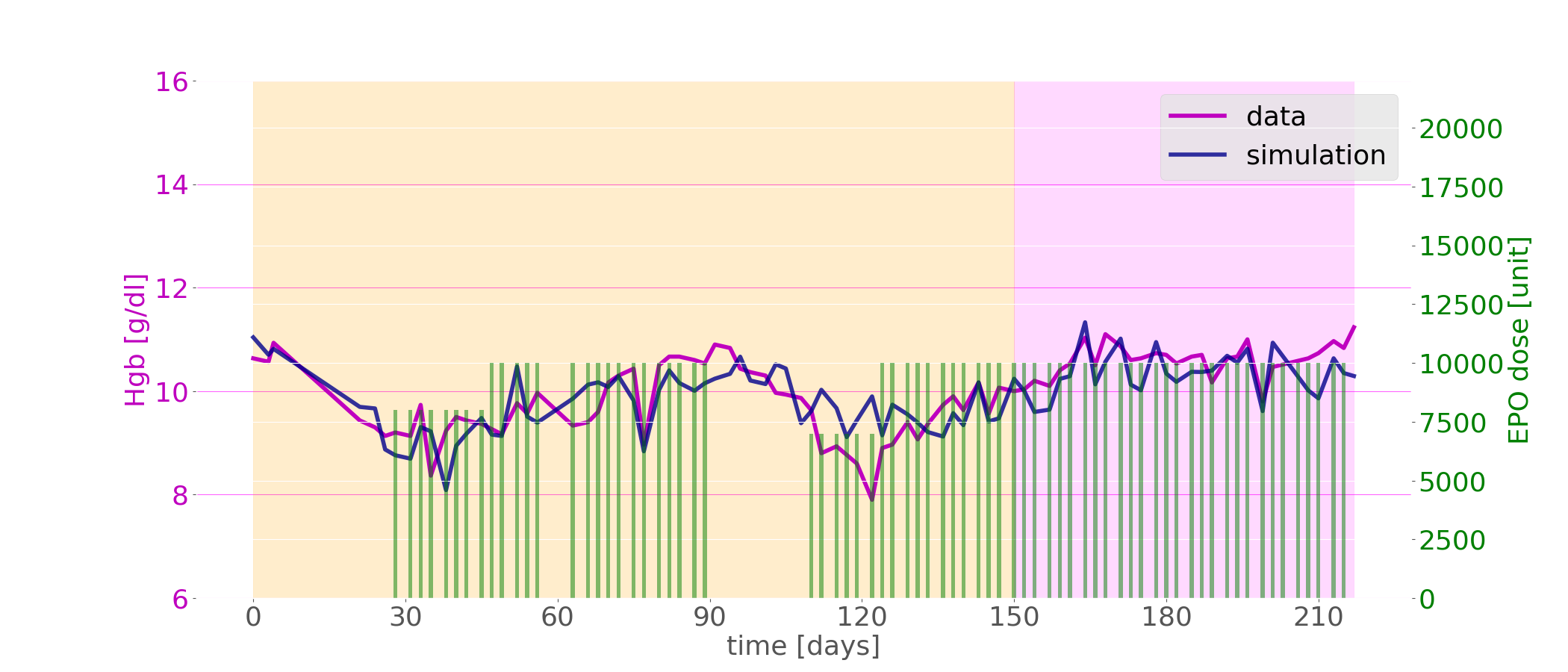

Supplement: S1 Figs — Pre-dialysis Hgb measurements (magenta) and model output (blue) during the model adaptation period (yellow area) and prediction period (purple area). Green bars represent the administered ESA doses. (ZIP) [file pone.0195918.s001.zip › patient_100055.png]

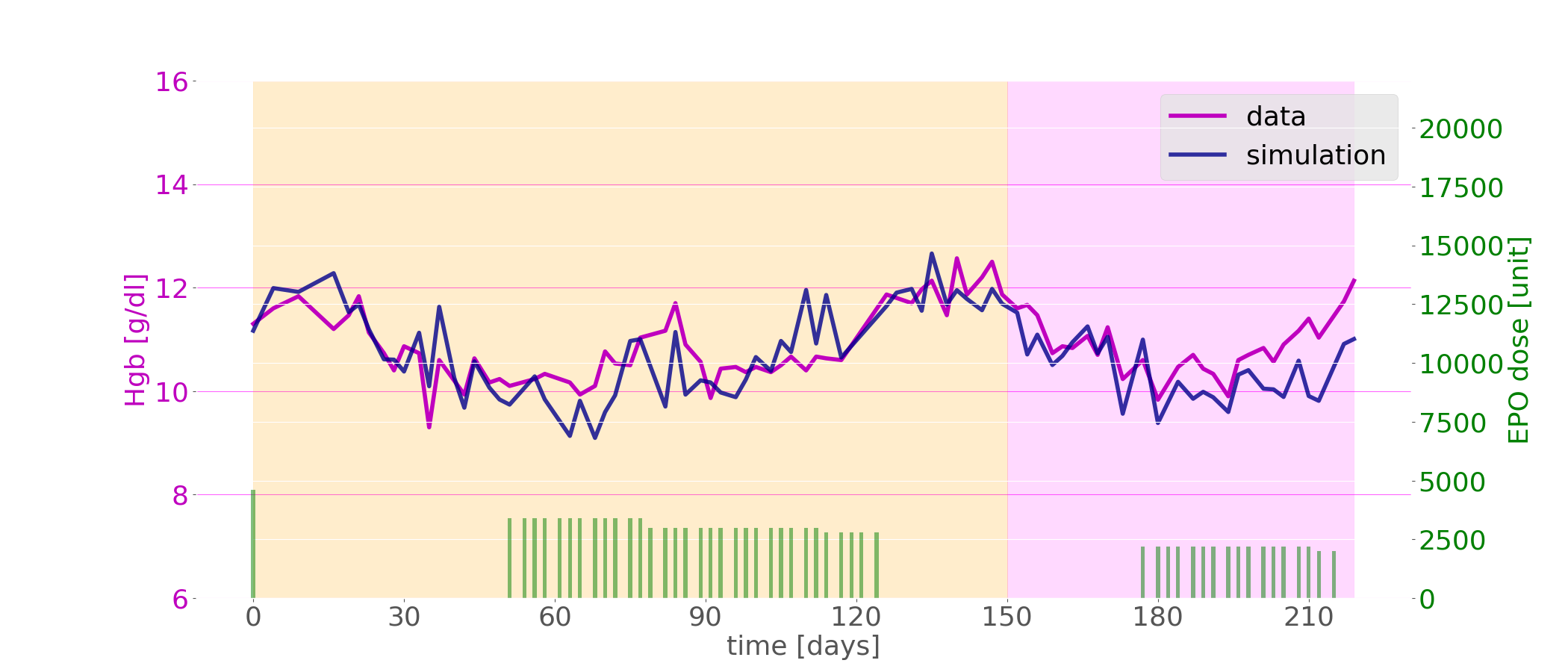

Supplement: S1 Figs — Pre-dialysis Hgb measurements (magenta) and model output (blue) during the model adaptation period (yellow area) and prediction period (purple area). Green bars represent the administered ESA doses. (ZIP) [file pone.0195918.s001.zip › patient_100056.png]

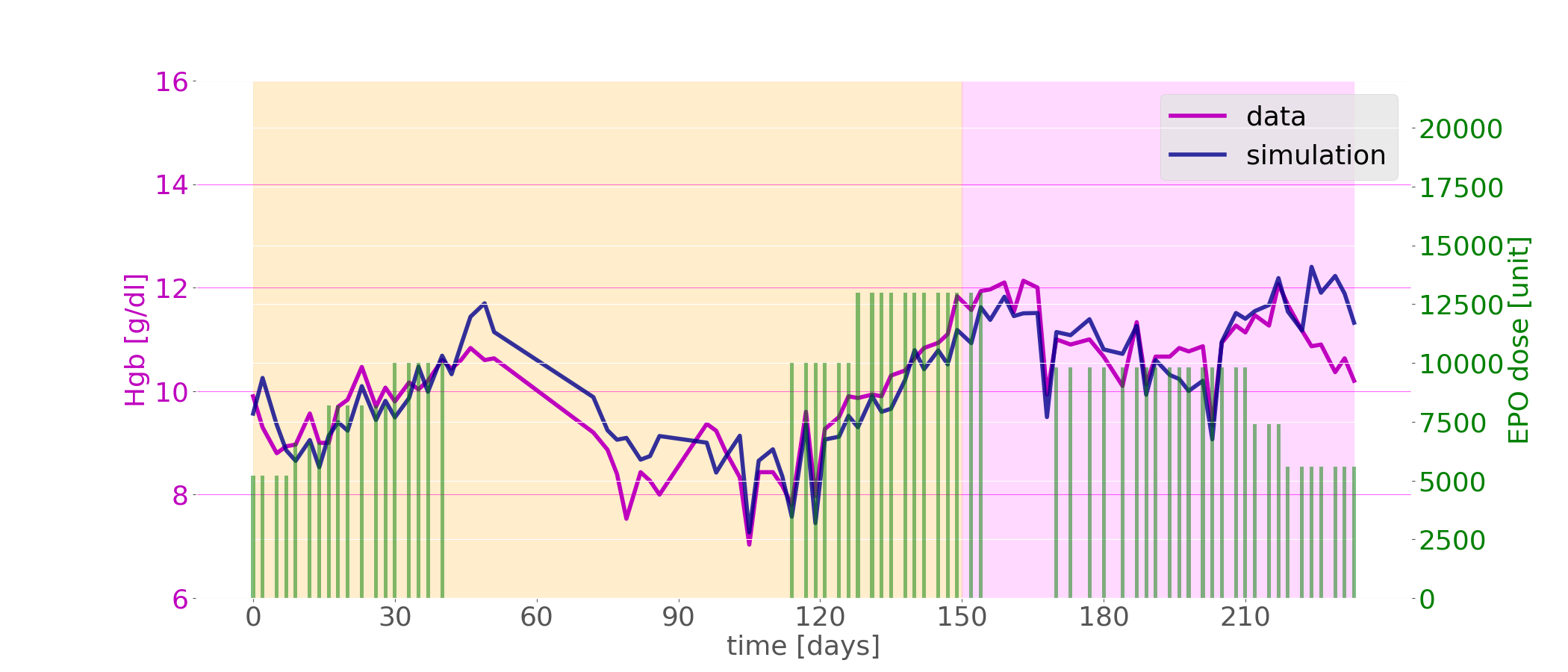

Supplement: S1 Figs — Pre-dialysis Hgb measurements (magenta) and model output (blue) during the model adaptation period (yellow area) and prediction period (purple area). Green bars represent the administered ESA doses. (ZIP) [file pone.0195918.s001.zip › patient_100057.png]

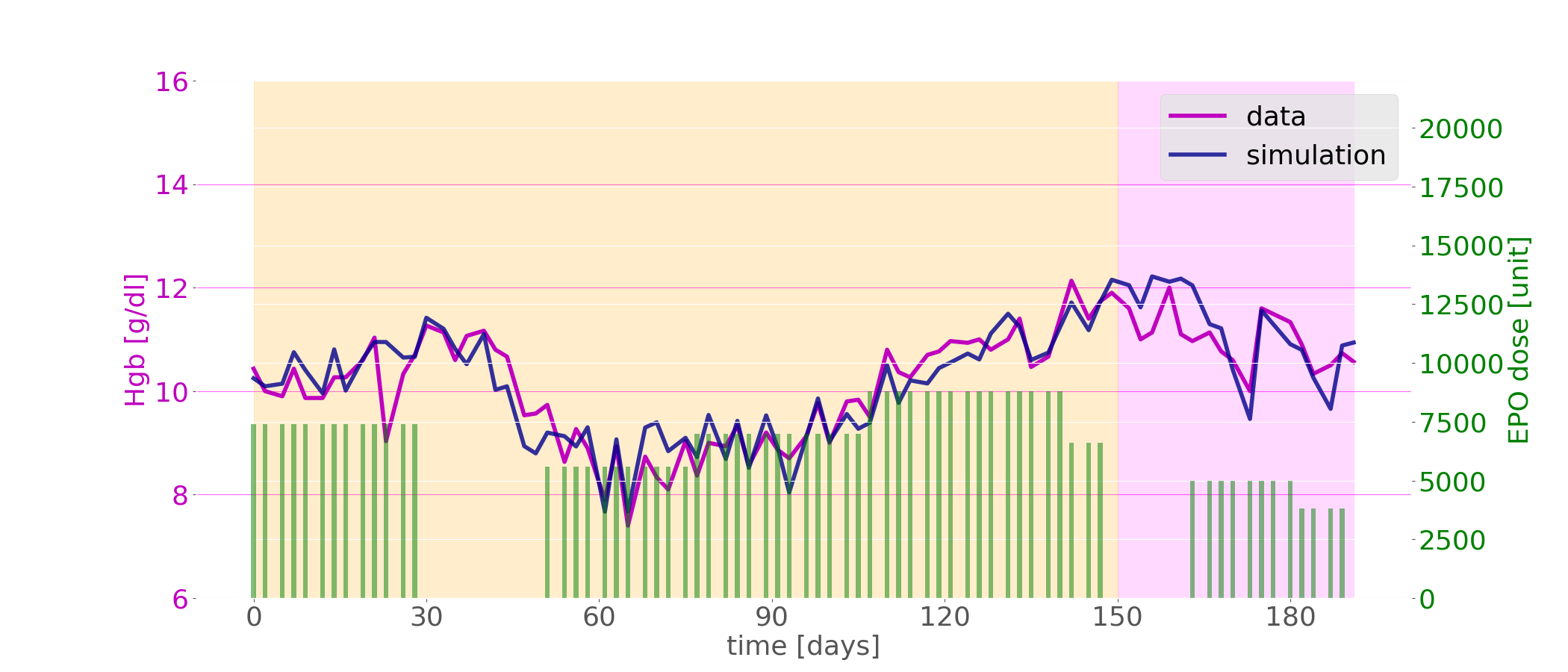

Supplement: S1 Figs — Pre-dialysis Hgb measurements (magenta) and model output (blue) during the model adaptation period (yellow area) and prediction period (purple area). Green bars represent the administered ESA doses. (ZIP) [file pone.0195918.s001.zip › patient_100058.png]

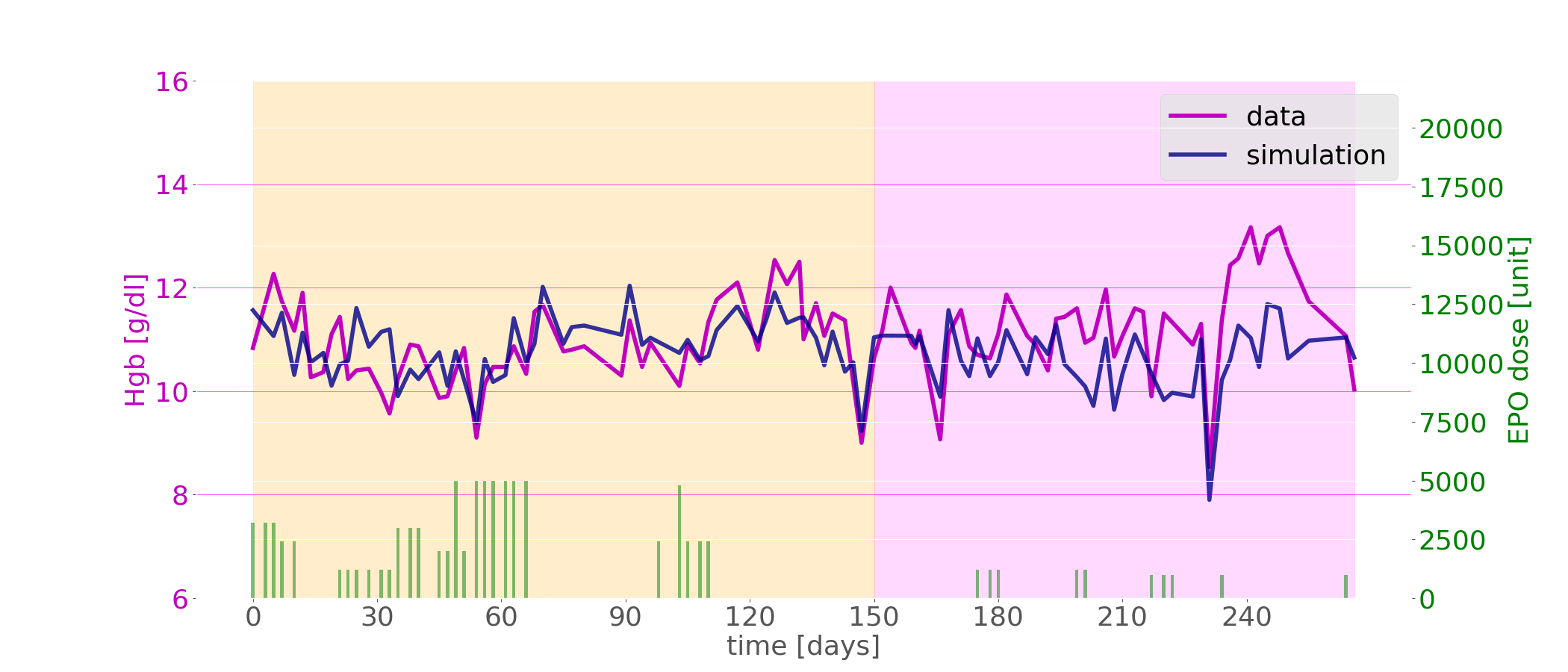

Supplement: S1 Figs — Pre-dialysis Hgb measurements (magenta) and model output (blue) during the model adaptation period (yellow area) and prediction period (purple area). Green bars represent the administered ESA doses. (ZIP) [file pone.0195918.s001.zip › patient_100059.png]

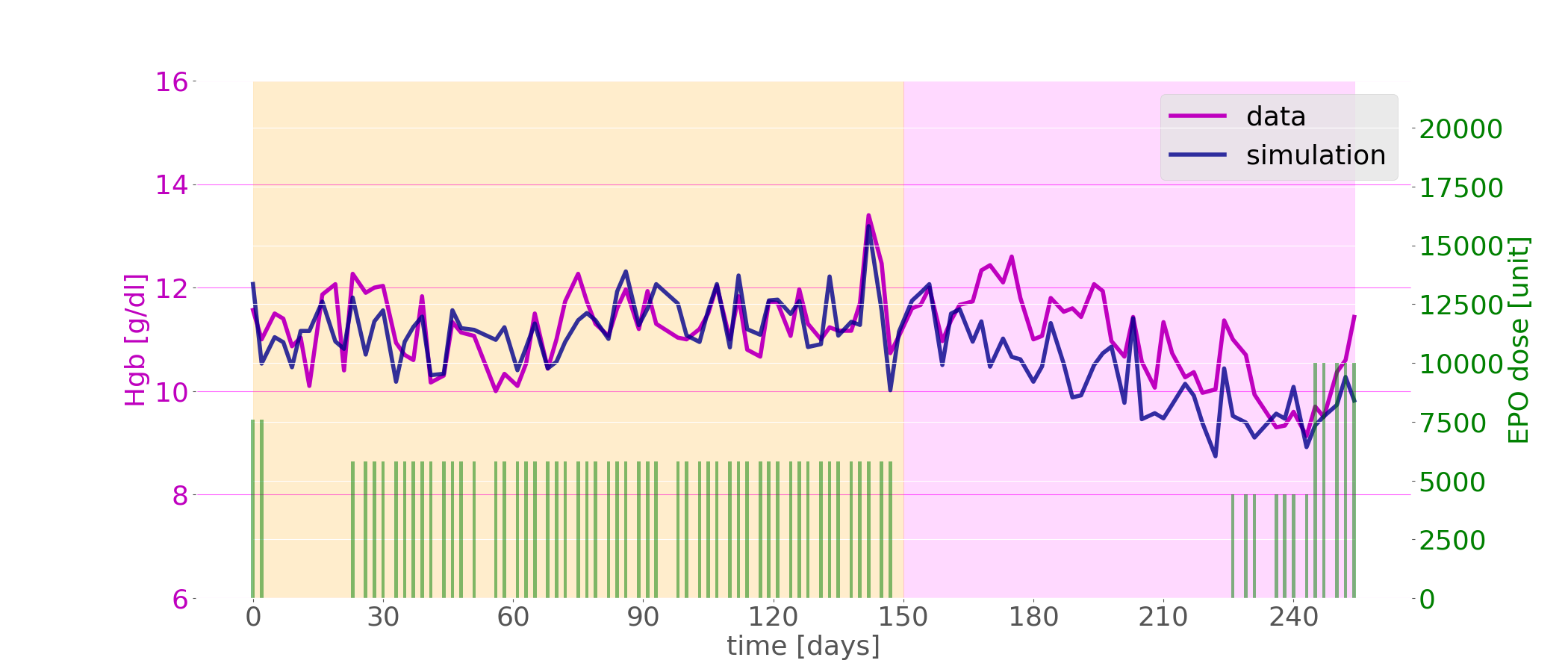

Supplement: S1 Figs — Pre-dialysis Hgb measurements (magenta) and model output (blue) during the model adaptation period (yellow area) and prediction period (purple area). Green bars represent the administered ESA doses. (ZIP) [file pone.0195918.s001.zip › patient_100060.png]
